# An *Emiliania huxleyi* pan-transcriptome reveals basal strain specificity in gene expression patterns

**DOI:** 10.1038/s41598-021-00072-5

**Published:** 2021-10-21

**Authors:** Ester Feldmesser, Shifra Ben-Dor, Assaf Vardi

**Affiliations:** 1 Bioinformatics Unit, Life Sciences Core Facilities, Weizmann Institute of Science, 7610001 Rehovot, Israel; 2 Department of Plant and Environmental Sciences, Weizmann Institute of Science, 7610001 Rehovot, Israel

**Keywords:** Marine biology, Data integration, Transcriptomics, Virus-host interactions

## Abstract

*Emiliania huxleyi* is a cosmopolitan coccolithophore widespread in temperate oceans. This unicellular photoautotroph forms massive recurring blooms that play an important role in large biogeochemical cycles of carbon and sulfur, which play a role in climate change. The mechanism of bloom formation and demise, controlled by giant viruses that routinely infect these blooms, is poorly understood. We generated a pan-transcriptome of *E. huxleyi,* derived from three strains with different susceptibility to viral infection. Expression profiling of *E. huxleyi* sensitive and resistant strains showed major basal differences, including many genes that are induced upon viral infection. This suggests that basal gene expression can affect the host metabolic state and the susceptibility of *E. huxleyi* to viruses. Due to its ecological importance, the pan-transcriptome and its protein translation, applicable to many *E. huxleyi* strains, is a powerful resource for investigation of eukaryotic microbial communities.

## Introduction

*Emiliania huxleyi* is a cosmopolitan unicellular photoautotroph that plays a prominent role in the marine carbon and sulfur cycles^1,2^. Its intricate calcite coccoliths account for a third of the total marine CaCO_3_ production, making it highly susceptible to future ocean acidification^3^. *E. huxleyi* grows in large-scale recurring blooms in the ocean, covering thousands of square kilometers, serving as the foundation of entire marine food webs. The blooms produce large amounts of dimethyl sulfide (DMS), emitted into the atmosphere, that can affect cloud formation^4^.

A unified and consistent transcriptome of *E. huxleyi* can be very useful to better elucidate the key metabolic and physiological traits shaping its unique life cycle. This includes interactions with its specific double stranded DNA virus *E. huxleyi* virus (EhV) that controls blooms in the marine environment. To this end we used two *E. huxleyi* assembled transcriptomes, the first derived from strain CCMP2090 which was infected by EhV for 1 and 24 hours^5,6^ and the second transcriptome derived from a virus-resistant *E. huxleyi* strain, CCMP373 and a virus-susceptible strain, CCMP374, in both exponential and stationary growth phases^7^.

This study, utilizing the unified transcriptome, provides new insights into the functional analysis of the second system, as in depth analyses of the viral infection system were published^5^, while the growth phase experiment was not fully analyzed^7^, and the functional changes were not presented. In addition, we performed a three-strain comparison of uninfected *E. huxleyi* during exponential growth. The gene expression of the strains is fundamentally different in basal growth, whether in exponential or stationary phase. This could have functional consequences in viral infection, as the basal gene expression patterns may prime the various lines for either sensitivity or resistance^8,9^.

This pan-transcriptome is a valuable resource for the community enabling analysis of gene expression in different conditions and different strains, as well as proteomics experiments, without the need for building the required databases.

## Results

### Unified transcriptome

Two de novo assembled transcriptomes for *E. huxleyi* were constructed based on different experimental frameworks using different strains. The initial transcriptome was defined as part of a viral infection study. *E. huxleyi* (strain CCMP2090) was exposed to its lytic (EhV201) and nonlytic (EhV163) viruses over a short time course of infection (1 and 24 h)^5^. In this time scale EhV163 does not produce virus and there is no cell death, and the expression profile at 24 h of infection is virtually identical to the non-infected control. The second transcriptomic set was constructed by co-assembling sequence reads from mRNA of exponential (2 days) and stationary (12 days) growth phases of CCMP373 and CCMP374 cells^7^. CCMP373 shows a high level of resistance while CCMP374 is sensitive to viral infection^10,11^.

**Figure 1:**
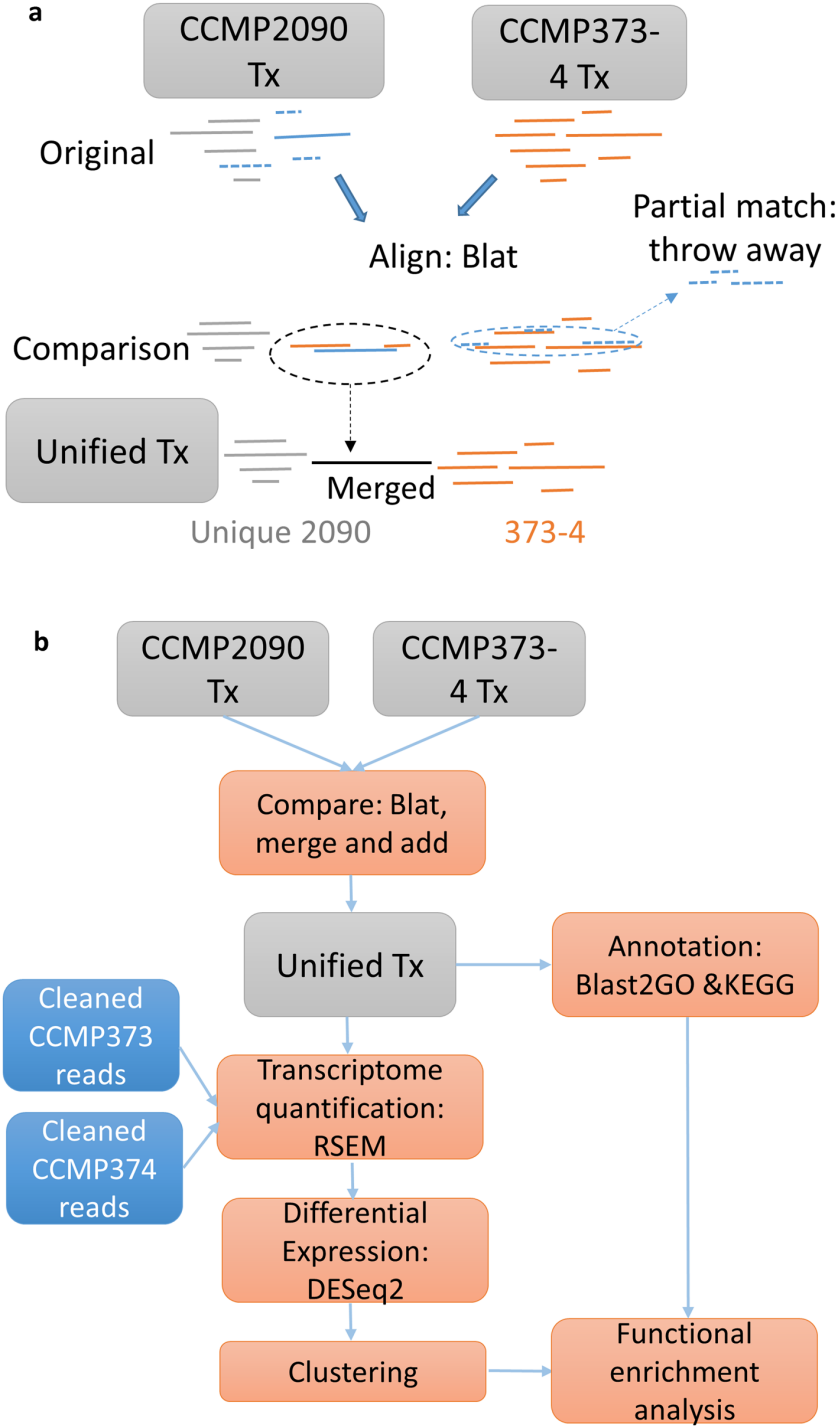
Automated pipeline for transcriptome unification. (**a**) Merging the genes defined in the CCMP2090 and in the CCMP373-374 transcriptomes when there is overlap between transcripts. Details in the text. (**b**) The full pipeline used to unify the two transcriptomes. First, genes were compared using Blat, with the CCMP373-374 transcriptome as the base. When matches were found, part of the transcripts were merged. Transcripts that were unique to the CCMP2090 transcriptome were added to the joint collection. Representative transcripts were cleaned from contamination, annotated, and quantified with the Illumina reads. Differential gene expression and clustering analysis were performed as well as functional enrichment analysis using GO annotations.

We combined the two sets to create one unified transcriptome (workflow in Fig. 1), to gain a more complete picture of the transcriptional landscape, since different growth conditions and viral infection enrich the number of genes being expressed and therefore detected. Initial tests were done manually to ensure mappability between strains, and full single nucleotide polymorphism (SNP) and short insertions or deletions (indel) analysis was performed subsequently (details below in the Genetic Variability section).

The two transcriptome sets were compared to each other, in order to identify transcripts that overlap and those that are unique to each database. Redundant transcripts were removed or merged, for details, see “Methods” and Fig. 1. Manually constructed and curated genes^5,6^ were added to the transcriptome database and their nomenclature adapted accordingly (Supplementary Table S1). The transcriptome was screened for contamination, and the contaminants removed. Partial chloroplast and mitochondrial genome sequences were removed, and replaced manually with the plastid protein coding genes as they appear in the GenBank database (accessions AY741371.1 and AY342361.1 respectively, both from strain CCMP373).

The final data set includes 156,898 transcripts belonging to 62,733 loci. The automatically built transcript sequence lengths span from 201 to 17,757 base pairs (bp) (Supplementary Fig. S1a). Analyzing the complete transcriptome, we found that 40% of the transcripts (62,327) are more than 1000 bp long, with an additional 27% (43,439) longer than 500 bp, and 24 longer than 10,000 bp. 13 of the longest transcripts were from the manually curated set, and match the length of their orthologs in other species.

The length of the representative transcripts is to some extent lower, 30% are longer than 1000 bp and an additional 22% are longer than 500 bp (Supplementary Fig. S1b). From this point on, the analyses concentrated on the representative transcript per locus, and the term ‘transcripts’ refers to these. 72% of the transcripts had open reading frames (ORFs) covering more than 70% of their length, and 53% had ORFs covering the entire length of the transcript (Supplementary Fig. S1c).

The full transcriptome as well as representative transcripts were mapped to the *E. huxleyi* CCMP1516 main genome assembly v1.0 draft^12^ using the software minimap2^13^ that allows for alternative splicing. 77% of representative transcripts (including 28% of the non-coding loci) and 85% of all transcripts mapped to the genome. Taking into account that the assembly was built de-novo and that there are gaps, mis-assemblies, and missing sequence in the draft genome, the results indicate a high quality transcriptome.

61,077 representative transcripts were translated into protein and 1,656 were non-coding RNA gene candidates. Additional statistics of the data set are shown in Table 1.

**Table 1:** Summary statistics of transcriptomes.

	CCMP2090	CCMP373-374	Manually constructed	Unified
Transcripts	75,092	186,712	240	156,898
Loci	na	105,811	240	62,733
Potentially ncRNA	na	4507	–	1656
GO annotated loci	29,868	37,147	240	24,792
KEGG annotated loci	5850	8022	240	6227
Proteins	na	101,304	240	61,077

Number of transcripts, loci, annotations per locus and proteins in the different transcriptomes. Na—not assessed.

### Transcriptome annotation

The representative transcripts were annotated using Gene Ontology (GO)^14^ terms by applying Blast2GO^15^ at the DNA level. 63% (39,314) of the transcripts had at least one BlastX hit, and GO terms were successfully assigned to 40% of the loci (24,792) (Supplementary Fig. S2a). The total number of annotations was 70,116 and the mean GO level for these annotations was 5.8. The most frequent organism in the Blast top hits was *E. huxelyi* CCMP1516 where most of the annotations are predictions, and not experimentally verified. The second most abundant species with 2336 hits was *Chrysochromulina* sp, also a haptophyte. The top GO terms in the categories biological process, molecular function, and cellular component were oxidation–reduction process, ATP binding, and integral component of membrane, respectively (Supplementary Fig. S2b).

Representative transcripts were also annotated with KEGG pathways. 6,227 genes were assigned to pathways. 92.6% of them were also annotated by GO. 463 (1.8%) were unique to KEGG, from a total of 25,254 annotated loci.

Validation of the manually constructed and curated gene sequences, performed either by PCR and/or Sanger sequencing and/or Western blot was already reported in two studies^5,16^ for 18 and 63 genes respectively. In addition, all of them have reads from RNA-seq data in at least one strain. 44 computationally defined genes were validated by real-time qPCR in^16^, and also had RNA-seq reads in at least one strain.

The BUSCO software^17^ was applied to check the completeness of the unified transcriptome. The search was performed against the Eukaryota database, including 303 conserved representative groups. When testing our representative transcripts, 81% were found (complete or fragmented) in the BUSCO groups searched. 174 were found complete, of them 168 were single-copy and 6 duplicated. 71 were fragmented and 58 missing. However, for the whole transcriptome the number of complete BUSCO was 194 and 88% were found including the fragmented ones (Table 2). These results are surprisingly good since *E. huxleyi* is a haptophyte, which are a distinct branch on the eukaryotic tree of life^12^ and therefore it is not expected that all of the BUSCO Eukaryota markers will be present.

**Table 2:** BUSCO search results in the representative transcripts (loci) and in the full transcriptome.

	Loci	Transcripts
Complete BUSCOs	174	194
Complete and single-copy BUSCOs	168	185
Complete and duplicated BUSCOs	6	9
Fragmented BUSCOs	71	74
Missing BUSCOs	58	35
Total BUSCO groups searched	303	303

All the quality control measures together are indicative of reliability and completeness of the transcriptome.

### Genetic variation between the strains

In order to validate the feasibility of utilizing the unified transcriptome for multiple strains, we performed SNP and indel calling. Reads from each of the three strains used to build the dataset, as well reads from an additional strain, CCMP379 from the Marine Microbial Eukaryote Transcriptome Sequencing Project (MMETSP)^18^ were assessed for genetic variation. This strain was chosen as it is also resistant to viral infection. The reads were mapped to the representative transcripts, and variants were called using GATK^19^. The results were hard filtered according to the recommendations for non-model organisms (https://gatk.broadinstitute.org/hc/en-us/articles/360035890471-Hard-filtering-germline-short-variants). Any position with a genotype differing from the unified transcriptome sequences was considered a SNP, whether homo- or heterozygous.

**Figure 2:**
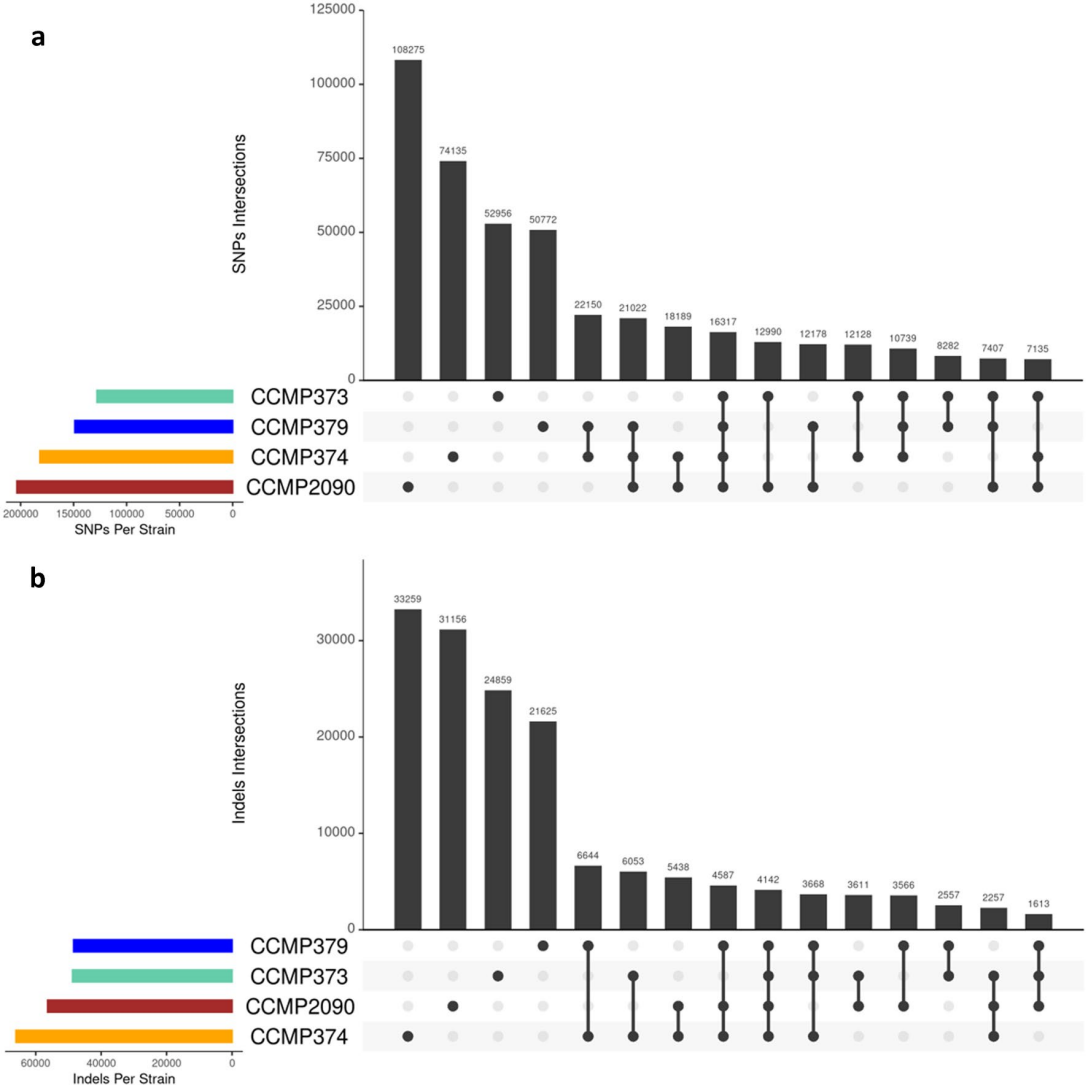
Upset plots showing the level of genetic variation in four *E. huxleyi* strains. The plots visualize intersections between the strains and the number of elements in each intersection for (**a**) SNPs and (**b**) indels.

Overall, 469,871 positions had SNPs in at least one of the strains. The number of SNPs per mapped base ranged between 0.003 and 0.005, per strain (Supplementary Table S2). On average, the number of SNPs per gene was 5.96 (CCMP2090), 5.58 (CCMP373), 6.07 (CCMP374) and 5.06 (CCMP379). As expected, CCMP373 had the least and CCMP2090 had the most SNPs to the reference, as a majority of the unified transcriptome sequences are from the CCMP373-374 dataset (Fig. 2a). Surprisingly, CCMP374 had more differences than expected, and is closer in number to CCMP2090 than CCMP373. There were 16,317 positions with SNPs that differ from the reference transcriptome, in all four strains assessed. It is interesting to note that strain CCMP379, which was not used in construction of the pan-transcriptome, has a similar SNP rate to the other strains.

In order to evaluate the distance between the strains, we inspected the SNPs in 18S ribosomal RNA. The only one of our studied strains with an annotated 18S sequence was CCMP374 (GenBank accession number: L04957.2). We mapped the reads from our four strains onto this sequence, and they were nearly identical (Supplementary Fig. S3). All strains, including CCMP374, have one common heterozygous SNP (T/C) at position 1168, although in CCMP373 and CCMP379 it is below the threshold for visualization in the Integrative Genomics Viewer (IGV). There is also a deletion of 2 bp in a small percentage of reads in all strains at the same point, del 1167–1168. CCMP373 and CCMP379 each have one additional unique heterozygous SNP (position 520 G/A and position 889 C/T respectively). We compared all of the *Emiliania huxleyi* 18S sequences from different strains that we could identify in GenBank to our starting sequence, a total of 30 sequences (Supplementary Fig. S4). There are only two positions in the entire alignment where there are SNPs that occur in more than one strain. In both cases CCMP374 is in the minority, as are our other three strains which match CCMP374 at those points.

We determined the number of SNPs in the BUSCO genes as a subset. There were 3112 positions with SNPs overall in the various strains. Both the number of SNPs per mapped base (range: 0.002–0.0034) and number of SNPs per gene (range: 3.4–5.8) went down in every strain (Supplementary Table S2) as compared to the numbers of SNPs in the full dataset.

Indels were also called and filtered, and limited to those 15 bp and shorter. We did not include longer ones, as there is pervasive intron retention in *Emiliania huxleyi*^6^ and we did not want alternate splicing to be called as indels. 155,035 indels were found in total, with the vast majority strain specific. CCMP374 and CCMP2090 had the most, and as with SNPs, CCMP373 and CCMP379 had the least (Fig. 2b).

Taken together, all evidence shows that reads from different strains can be mapped to the unified transcriptome (for some specific examples see Supplementary Fig. S5).

### Differential gene expression

As a proof of concept of utility of the pan-transcriptome, we analyzed the CCMP373-374 data obtained in^7^ for differential expression. This analysis gave us the opportunity to gain insight into the molecular differences between the strains CCMP373 and CCMP374 in two stages of growth, exponential (2 days) and stationary (12 days). The general flow of the analysis is shown in Fig. 1. The cleaned reads were mapped to the unified transcriptome and quantified per gene using the RSEM software^20^.

Genes that had at least 7 normalized reads in at least 3 samples were considered expressed. 42,719 genes from the 62,733 loci in the data set were expressed, of them 32,087 differentially expressed in at least one pairwise comparison (Supplementary Table S3).

**Figure 3:**
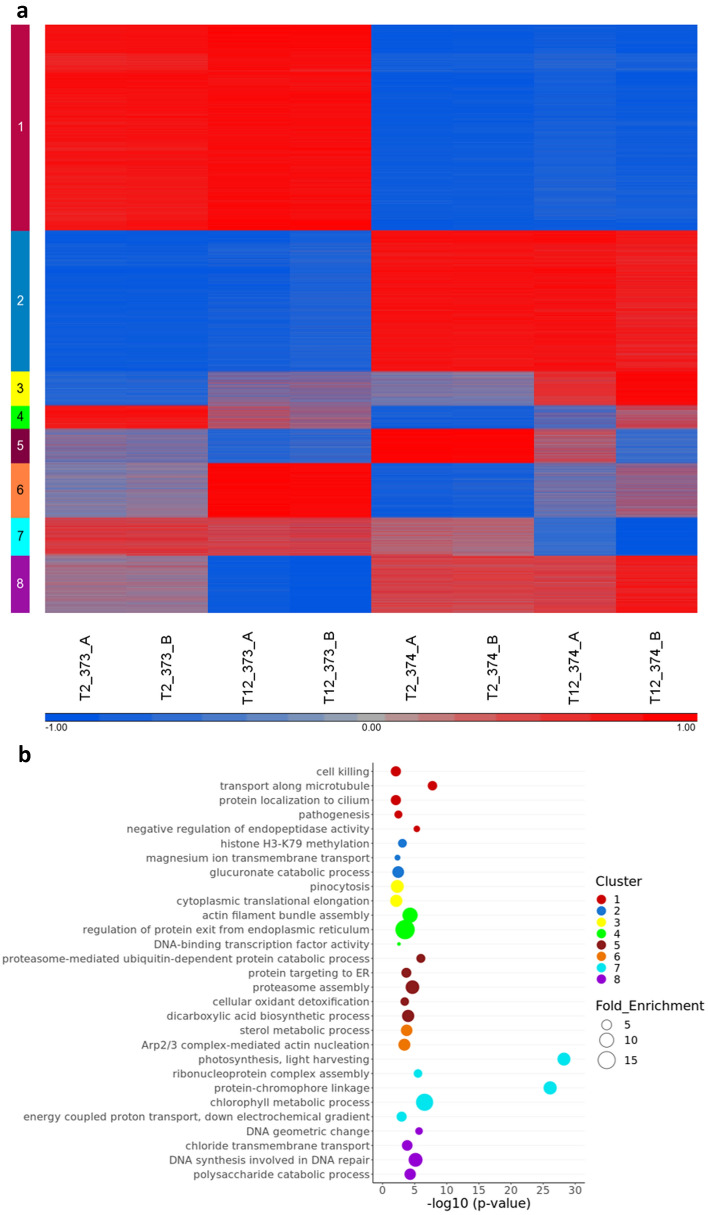
Global gene expression profiles of *E. huxleyi:* Differentially expressed loci in CCMP373-CCMP374. (**a**) K-means clustering of genes with altered expression levels between strains and growth states is presented. The expression levels are standardized by gene. T2-day 2 of growth, T12-day 12 of growth. A and B are biological replicates. Red, high expression level; blue, low expression level. (**b**) Top significantly enriched GO terms (hypergeometric test, *p* value < 0.05) related to gene clusters displayed in (**a**). Colors refer to clusters as indicated in (**a**). For a full list of enriched GO terms in each cluster, see Supplementary Table S4.

Clustering was performed on the differentially expressed genes, and eight clusters were defined. As can be seen in Fig. 3a, there is a clear separation between strains. The top two clusters that included almost 60% of the differentially expressed genes (18,912 genes) had no differences between growth states, but emphasize the strain differences, while the others have differences in expression between exponential and stationary growth. The global differences in expression patterns point to a priori gene expression that may be related to viral sensitivity or resistance.

The exponential phase (T2) gene expression pattern is much closer between replicates, regardless of strain, than those of the stationary phase (T12). As we are comparing two days to twelve days of growth, there is more opportunity for a divergence in the cultures, resulting in less concordance between the replicates.

In clusters 3, 5, and 6, there are differences between the growth states, and the trend, not while always strong, is similar in both strains. In cluster 4, the two strains show opposite trends of change over the growth states; strain CCMP373 genes are upregulated in exponential growth, while strain CCMP374 genes are slightly upregulated in stationary growth. In clusters 7 and 8, genes are changing only in one of the strains, showing differences in the ways each strain reacts to growth states.

### Functional analysis of the differentially expressed genes

Functional analysis of enriched terms in GO was performed for each cluster to get biological insight on exponential versus stationary growth, resistant versus sensitive strains in a combined comparison (Fig. 3b, full results in Supplementary Table S4). Each of the clusters was analyzed using the Ontologizer tool^21^ with the topology weighted method^22^.

Cluster 1, that showed gene upregulation in strain CCMP373 in comparison to strain CCMP374, is enriched in the following biological processes: cilium organization/transport, microtubule based movement, dynein complex, cell killing, and negative regulation of endopeptidase activity. The complementary cluster 2 with gene upregulation in strain CCMP374 displayed enrichment in histone H3-K79 methylation, magnesium ion transmembrane transport, and glucuronate catabolism. Enrichment of functions related to actin filament bundle assembly, regulation of protein exit from endoplasmic reticulum and plant-type cell wall organization were found in cluster 4, where the two strains show opposite reactions to growth saturation.

Genes involved in changes in translation were enriched mainly in clusters 3 and 5. Cluster 3 was also enriched in genes involved in negative regulation of cellular carbohydrate metabolism and cluster 5 in cellular oxidant detoxification. Sterol metabolism and Arp2/3 complex-mediated actin nucleation were enriched in cluster 6. In clusters 7 and 8, where only one of the strains genes are downregulated at growth saturation, the enriched biological processes are photosynthesis related (cluster 7); and DNA geometric change and regulation of membrane lipid distribution (cluster 8).

### Unique per strain: comparison of the three strains

**Figure 4:**
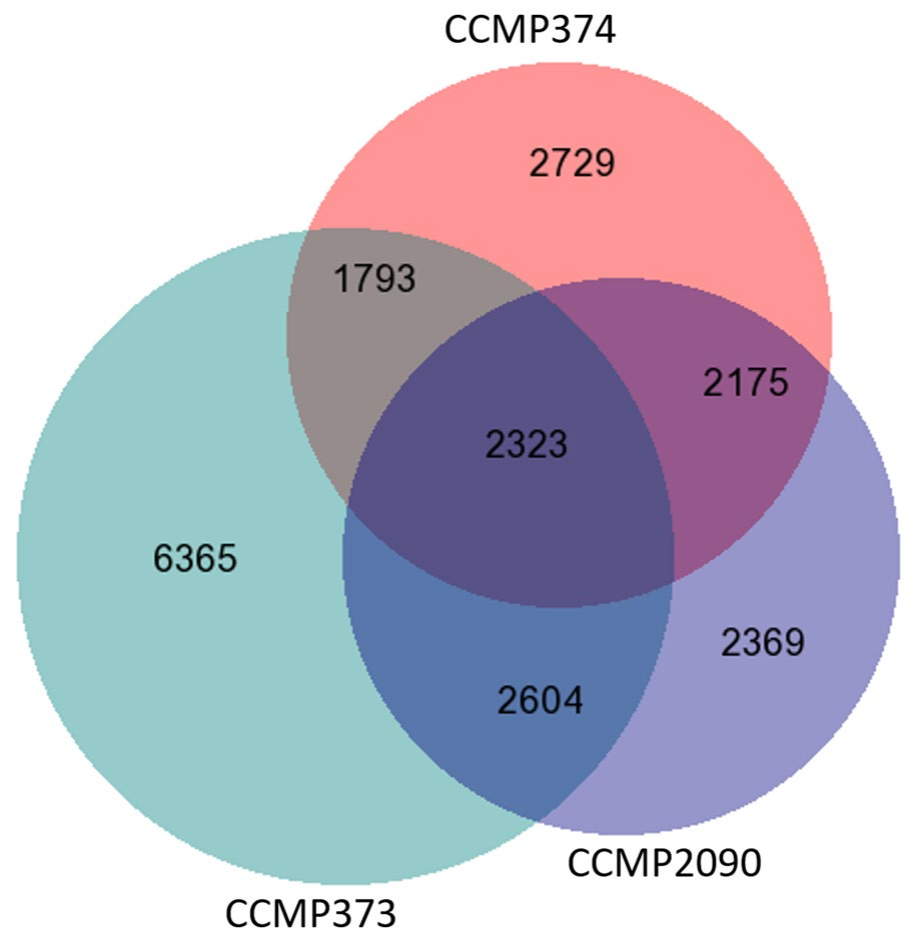
Digital unique expression. Proportional Venn diagram of the genes expressed in each of the strains in exponential growth, and their overlaps. The diagram was drawn using BioVenn (https://www.biovenn.nl/index.php).

To assess strain differences during exponential growth, we wanted to compare the three strains CCMP373, CCMP374 and CCMP2090. Since the CCMP2090 experiment was performed using a different version of the sequencing instrument and had strong batch effects when compared to the second experiment, we looked at genes that were expressed uniquely in each one of the three strains (Fig. 4) in each experiment separately. Genes that had at least 7 normalized reads in two replicates from day 2 for strains CCMP373 and CCMP374 or in the non-infected CCMP2090 sample from day 1, were considered expressed in exponential growth.

6365 genes were expressed exclusively in CCMP373. 2729 and 2369 uniquely expressed genes were found in strains CCMP374 and CCMP2090, respectively. In addition, 2175 genes were shared by both sensitive strains (CCMP2090 and CCMP374) in exponential growth.

**Figure 5:**
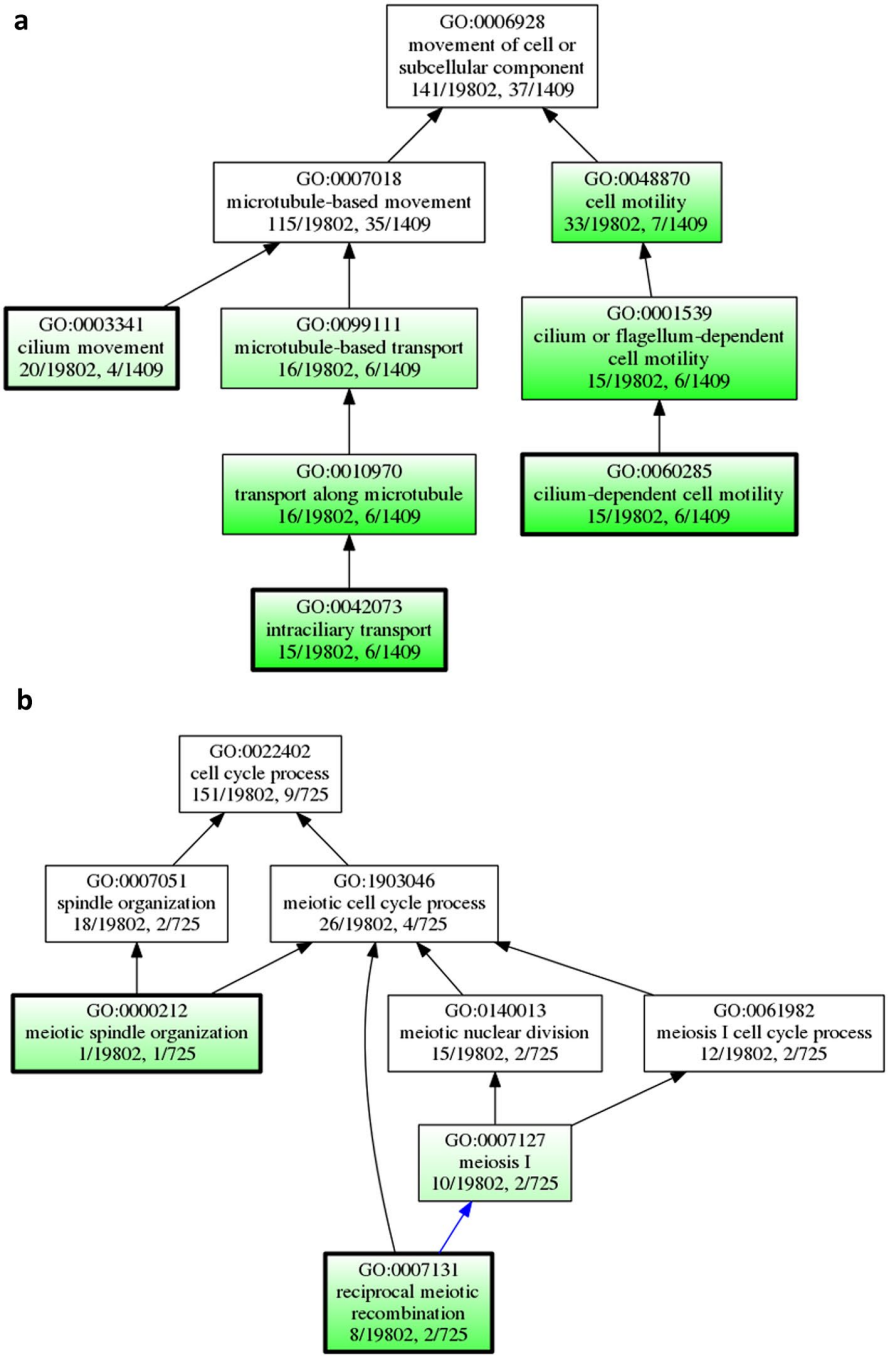
Functional GO enrichment analyses of uniquely expressed genes in resistant and sensitive strains. Subgraphs of some enriched terms are shown: (**a**) GO analyses of cell motility process and related cilium terms, enriched in CCMP373 uniquely expressed genes. (**b**) GO analyses of meiosis related terms, enriched in CCMP374 and CCMP2090 uniquely expressed genes. Terms in green are significantly enriched. The colored arrows signify special relationships: blue is a “part of” the next term down.

The biological processes enriched exclusively in CCMP373 at exponential growth were dynein, microtubule motor activity, sulfuric ester hydrolase, cilium, negative regulation of peptidase and transport along microtubule (Fig. 5a, Supplementary Table S5). These are in concordance with the enriched terms in cluster 1 (CCMP373-CCMP374 comparison) that includes genes upregulated specifically in strain CCMP373. For strain CCMP374 there is enrichment in actin-dependent ATPase, transmembrane transport, cell projection membrane (pseudopodium) and tubulin folding. The top enriched functions for the strain CCMP2090 were calcium ion, modified amino acid, lipid binding, G- protein-coupled receptor activity, porin activity, calcium channel activity, pore complex, cell outer membrane and bacterial-type flagellum.

Terms uniquely related to sensitive strains were galactosyltransferase activity, intrinsic component of membrane, sulfuric ester hydrolase activity, glycosylation, phosphatidylinositol kinase activity, mismatch repair and meiotic spindle organization (Fig. 5b).

Together, the different expression patterns and the genes uniquely expressed by strains indicate that the pan-transcriptome of *E. huxleyi* is highly dynamic, as it integrates two levels of plasticity, the regulatory and the genetic information levels^12^.

### Basal expression differences may influence response to viral infection

Specific gene sets (Supplementary Table S1) have been shown to change their expression patterns upon viral infection^9,23,24^, but little is known about the basal levels of expression in resistant (CCMP373) and sensitive strains (CCMP374). We assessed the expression level of gene sets related to: (a) 1N cell specific genes, among them flagellar markers^9,25^, phototropins and one MYB transcription factor (called herein S-cell genes according to^26^), (b) meiosis markers^9^, (c) redox related genes^23^ and (d) cell death related genes^27^.

**Figure 6:**
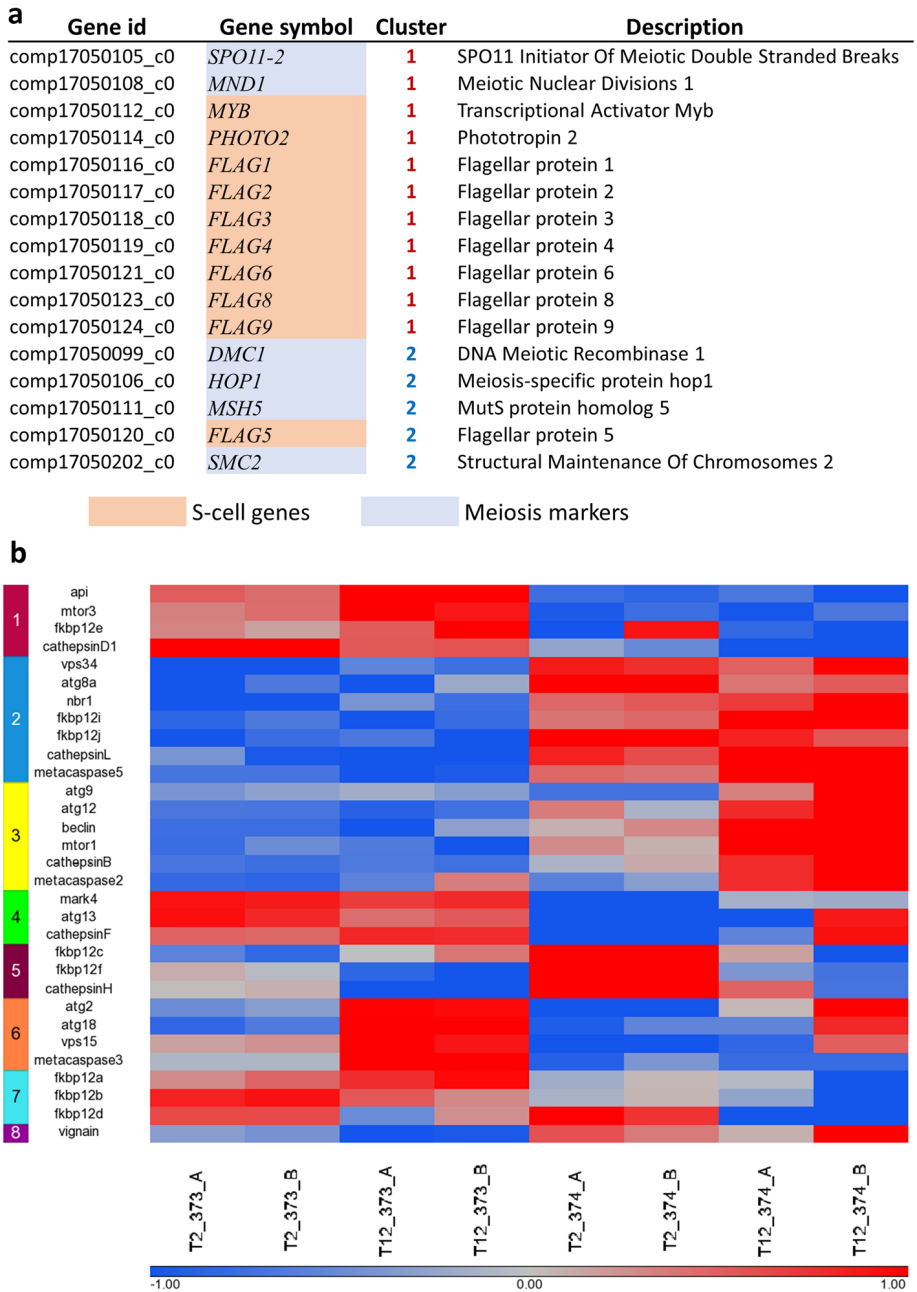
Examples of specific gene sets. (**a**) S-cell and meiosis genes that were identified in clusters 1 and 2, S-cell genes are enriched in cluster 1 and meiosis genes are enriched in cluster 2 (**b**) Genes related to autophagy and cell death, as they appeared in the K-mean clustering in Fig. 4a.

We found that the majority of the S-cell genes are upregulated in CCMP373 versus CCMP374. In contrast, meiosis markers are highly expressed in cluster 2, the opposite of cluster 1 (Fig. 6a). These results are in agreement with enriched GO terms in cluster 1 such as microtubule based movement (GO:0007018) and dynein complex (GO:0030286) (Fig. 2B), and in the three stain comparison, the related term microtubule motor activity (GO:0003777) is found in genes expressed exclusively in CCMP373 (Fig. 5a). Meiosis related genes were enriched in the two sensitive strains, CCMP2090 and CCMP374, in the three strain comparison as well (Fig. 5b).

ROS related genes are enriched in cluster 5 (4.5 fold enrichment, hypergeometric test *p* value 0.004, Supplementary Table S6), which shows a trend of higher expression in exponential growth, and lower expression in stationary phase, though overall expression is much higher (in both time points) in strain CCMP374. This also matches the GO enrichment of cellular oxidant detoxification genes (GO:0098869) found in this cluster.

We found that many autophagy and cell death genes are expressed at a lower level in CCMP373 as compared to CCMP374 (clusters 2, 3, 5 and 8) (Fig. 6b). In cluster 3, in particular, key genes in all the major autophagy and cell death pathways are downregulated (mtor1, atg6/beclin, atg9, atg12, metacaspase2, and cathepsinB).

## Discussion

### Unified transcriptome assembly

We present a pan-transcriptome for *Emiliania huxleyi*, based mainly on three different strains (CCMP2090, CCMP373, and CCMP374), and two separate transcriptome builds that were merged. The first transcriptome set was constructed in the early days of NGS, and was in a viral infection system. In that dataset, we decided to include E. huxleyi ESTs from GenBank as well (over 129,000), as they introduced longer sequences and allowed better gene construction. The ESTs were from four different strains, CCMP1516 (59%), RCC1216 (15%), RCC1217 (15%) and CCMP371 (11%). The transcriptome definition was partially genome-guided, and the genome is from strain CCMP1516^12^. The genome of *E. huxleyi* is complex, as it has 68% GC content and has approximately 60% genomic repeats, that are not well defined. The genome build, a first draft, consequently has many mis-assemblies, including false duplications, fragmented genes between contigs, and contigs that were left out of the assembly^6^. At the time there were no purpose built transcriptome assemblers, rather genome assemblers that were repurposed for transcriptomes. Trinity, which was used for the second transcriptome build, was developed expressly for this purpose, without requiring a genome, and is still considered one of the best assemblers^28^. The second transcriptome build was from a time course experiment, and performed on a newer instrument, with greater read depth and paired end reads. Therefore, both due to the greater read depth and the improved assembly technique, we used the second transcriptomic set as the base for the unified transcriptome.

Integration of the two individual datasets was performed on the basis of sequence similarity. Transcripts from CCMP2090 that had no hits in CCMP373-374 were added to the unified dataset. Transcripts that had matches, however, had to be evaluated for removal or inclusion. In the interests of both comprehensiveness and accuracy, while minimizing redundancy, we decided to prefer the CCMP373-374 transcripts and to remove the CCMP2090, even if there was a partial overlap. Only when there was extensive overlap of a CCMP2090 transcript with a transcript on both sides from CCMP373-374 was a merger performed (essentially using CCMP2090 to fill in gaps in fragmented transcripts). This was performed with stringent cut-offs, in order to minimize the chances of forming chimeric transcripts of non-related genes, while allowing longer transcripts to be formed.

### Genetic variation

*Emiliania huxleyi* strains are remarkably similar based on comparison of 18S rRNA sequences (1721 bp) of 33 strains, where only two SNPs appeared in more than two strains, and all strains but one had at most two or three SNPs.

Prior to construction of our first transcriptome set, we manually defined genes based on both genome sequence and ESTs, which were from four different strains. Therefore, we knew that there was very little variation in coding genes as well (data not shown). This was the basis for our decision to combine strains (via ESTs) in our CCMP2090 dataset, and later, the unification of the two transcriptome sets. To visualize this, a selection of manually constructed and curated genes, as well as some automatically defined genes were inspected to see if reads could be mapped correctly across strains. There were occurrences of 0–2 changes per 100 bp between strains, which falls within the allowable range of read alignment (for examples, Supplementary Fig. S5). This is consistent with a recent report comparing 10 related *Gephyrocapsa* strains, including four *E. huxleyi* strains, and 6 related genomes from other species. Bendif et al.successfully mapped genomic reads across all strains to one genome, and overall, a SNP rate of less than 3% was seen^29^. Performing global SNP calling on the unified transcriptome, using reads from four strains, we see an even greater similarity, with an average of 3–5 SNPs per thousand bases. This is expected, as the gene encoding portion of the genome is more conserved than the genome overall, and is further strengthened when evaluating the BUSCO genes, which are even more conserved.

### Functional analysis

To validate the utility of the pan-transcriptome, we performed global differential expression analysis between the strains CCMP373 and CCMP374 at two stages of growth. Most work to date comparing various *E. huxleyi* strains have concentrated on single physiological traits, many in the context of viral infection^7,9,30–32^.

In our dataset, the vast majority of differentially expressed genes were due to difference of strain, and not of time point. In order to pinpoint strain specific genes during exponential growth, we added data from an additional strain, CCMP2090. We further inspected the genes specific to each strain, and those common to CCMP374 and CCMP2090, as they are both sensitive to viral infection. We decided to concentrate on gene sets previously shown to have relevance to viral infection (S-cell, meiosis, ROS and cell death related genes) and see their basal expression patterns in the various strains.

*E. huxleyi* is known to have a biphasic life cycle including a calcified non-flagellated 2N cell stage and a flagellated non-calcified 1N cell stage^8,33^. Flagellated haploid cells are resistant to viral infection, and diploid cells can convert to haploid as a way to escape the virus^8,33^. This phenomenon, known as the “Cheshire Cat” strategy, has also been found to be decoupled from meiosis, resulting in morphologically changed 2N (or nearly 2N) cells expressing genes reported to be specific to 1N cells, the S-cell genes^9^. S-cells genes tend to be upregulated in CCMP373 as compared to CCMP374 (cluster 1). This is in agreement with the enriched GO terms for this cluster. Moreover, dynein chain genes have been found in EST libraries from 1N but not from 2N cells (from two additional strains, RCC1217 and RCC1216, respectively)^25^, which is in concordance with our results. These genes have been tested in two sensitive strains (RCC1216 and CCMP2090)^9^, and show upregulation in a long time course of infection, as detected by qPCR.

Redox related genes, particularly those involved in regulation of reactive oxygen species (ROS) metabolism, have been shown to participate in algal response to environmental stress during oxidative stress and senescence^34–36^. ROS-related genes have also been suggested to mediate responses related to viral infection progress and induction of cell death during the lytic phase of infection^23^. During a short term infection of the sensitive CCMP2090 strain, these genes change their expression patterns^23^. On the basal level, cluster 5 is enriched for ROS related genes, with higher expression in CCMP374 than in CCMP373.

Autophagy is known to play an important role during viral infection^24^, in recycling of lipids essential for viral assembly and for production of EhV membranes. The basal protein expression level of a metacaspase has been found to be higher in sensitive than in resistant *E. huxleyi* strains^37^. As expression of autophagy and cell death related genes enable viral infection, CCMP373, which has a very low or missing expression of these genes in the basal state, may be protected. This suggests a novel mechanism of resistance, where some strains are primed for infection, and others primed for protection.

It has been shown in other algae^34^ that death is induced in cells which previously had high levels of oxidative stress and antioxidant activity. This correlates well with the gene expression patterns seen in the CCMP374 strain, where both ROS and cell death related genes are highly expressed relative to CCMP373 on the basal level. We wanted to see if these observations were consistent in other sensitive and resistant strains. The only other high throughput dataset we could find was in the Moore collection with strains CCMP379 (resistant) and CCMP374 (sensitive) to viral strain EhV86^18^. The samples were collected at 2 and 24 h post infection, one each of control and virus per strain. We examined the genes where we had seen change at the basal level (S-genes, meiosis genes, ROS cluster 5 and cell death cluster 3; Supplementary Fig. S6). In general, the results were consistent, with the exception of the ROS related cluster 5 genes, especially taking into account the differences in experimental systems (laboratories, instruments, strains, growth conditions and time courses). The differences in basal levels of expression give intriguing hints as to which genes may be involved in tendencies to resistance and sensitivity and requires further study to be better understood.

In conclusion, the *E. huxleyi* pan-transcriptome can be used as a basis for omics studies from various strains^38,39^, and even from ocean blooms that have mixed and novel strains (as seen in current work in our lab). It has the potential to generate insights into the molecular basis for the ecological success of *E. huxleyi* as a bloom-forming algal species, and changes in gene expression in response to different environmental conditions.

## Methods

### Experimental systems

Full details of the experimental systems can be found in the following papers^5–7^. We briefly describe them here.

Transcriptomic Set 1: *Emiliania huxleyi* strain CCMP2090 was cultured in k/2 medium. All experiments were performed with exponential phase cultures (5 × 10^5^ to 10^6^ cells·mL^−1^). *E. huxleyi* was infected with EhV201 and EhV163 in a 1:50 volumetric ratio of viral lysate to culture (multiplicity of infection of ∼1:1 viral particles per cell). polyA mRNA was isolated, and libraries prepared according to standard methods. RNAseq was performed for six samples using the Illumina HiSeq2000 in one run, one lane per sample, single 100 bp read, as follows: control (no virus) and infected with EhV201 or EhV163, at two time points: 1 and 24 h post infection.

Transcriptomic Set 2: *Emiliania huxleyi* strains CCMP373 and CCMP374 were cultured in F/2 medium. RNA from duplicate cultures was harvested at 2 and 12 days. polyA mRNA was isolated and libraries were prepared according to standard methods. Paired end reads (100 bp each) were sequenced from each culture at each time point (total of 8 samples) on an Illumina HiSeq2000, in one run, with one sample per lane.

### Transcriptome construction

The CCMP2090 transcriptome was assembled by integrating ESTs and Illumina short read sequences. CLC (de novo), Cufflinks (genome based)^40^, Partek® Genomics Suite™ version 6.5, Partek Inc., St. Louis, MO, USA and Cap3 (for combination of ESTs and assembled transcripts) assembly software were applied. Manual gene definition of 100 genes served as a base for quality control of the CCMP2090 transcriptome^6^. The CCMP373-374 transcriptome was co-assembled using the Trinity software^41^ with a minimum transcript size of 201 bp and was used as the basis for the unified transcriptome.

Blat^42^ was applied to compare both transcriptomes, to identify transcripts that overlap and those that are unique to each database. CCMP2090 transcripts that did not overlap at all with the CCMP373-374 transcriptome were added to the unified database. Trinity automatically defines loci and isoforms, assigning isoforms to loci, while our original build method^6^ did not, therefore we performed clustering into loci on the CCMP2090 transcripts that were added. Clustering was performed using BLASTClust from the NCBI BLAST package (https://www.ncbi.nlm.nih.gov/Web/Newsltr/Spring04/blastlab.html), requiring a match to have at least 95% identity over an area covering 20% of the length on both members of the sequence pair (-a 4 -p F -S 95 -L 0.2 -b T). They were named from comp17010001 to comp17025170, for a total of 15,170 loci that included 15,384 transcripts.

2090 transcripts that overlapped with two transcripts of the CCMP373-374 assembly, one at the 5’ end and one at the 3’ end, were merged in a longer transcript that included the three (Fig. 1a) according to the following rules: 1. A minimum blat score of 100; 2. The gaps in the alignment for each pair should not be more than 200 bases; 3. A non-overlapping sequence of up to 100 bases was allowed at the ends of the merged transcripts. The unmatched bases were allowed in order to take into account the fact that more errors occur at the ends of de novo transcript assemblies.

To keep the nomenclature of all transcripts consistent, the Trinity naming was applied to the newly added and the newly merged transcripts. The merged transcripts, 7548 in number, were named from comp17000000_c0_seq1 to comp 17007547_c0_seq1. If there were additional splice variants in the same locus (according to the Trinity assembly), they were given the same comp number and a new seq number.

For further research on specific genes, 240 genes were manually defined (detailed methods in^5,6^). To avoid redundancy with the automatically built transcripts, they were compared to the unified transcriptome with Blat^42^ with default parameters except for a minimum score of 200. All transcripts overlapping with manually defined sequences were discarded from the final transcriptome. The manually defined genes were named starting from comp17050001_c0_seq1.

The full process of unifying the transcriptomes was performed partly manually using Excel functions and partly using in-house perl scripts (scripts deposited in Figshare, see Code Availibilty).

The longest transcript for each locus was chosen as the representative transcript.

### Genetic variant calling

Sequence reads from each strain were merged into a fastq file and the fastq files were aligned to the representative transcripts using the BWA-MEM algorithm (v 0.7.17)^43^ with the read groups option (-R) to keep the strain identity. Following mapping, duplicates were detected and marked using the Picard MarkDuplicates tool in the Picard package (v 2.22.8) (https://broadinstitute.github.io/picard/). The resulting bam files were sorted according to coordinates and indexed using Samtools (v 1.9). Variant calling was performed using the GATK HaplotypeCaller (v 3.7) (https://gatk.broadinstitute.org/hc/en-us)^19^ to create GVCF files. Joint calling was performed using the GATK GenotypeGVCFs tool. The variants were divided into two files, one for SNPs and one for indels using the GATK SelectVariants. Both were hard-filtered according the GATK recommendations for non-model organisms (https://gatk.broadinstitute.org/hc/en-us/articles/360035890471-Hard-filtering-germline-short-variants) with the VariantFiltration tool. The options used for the SNPs were QUAL < 40.0, FS > 60.0, SOR > 3.0 and MQ < 40.0 and for the indels QUAL < 40.0 and FS > 200.0. In addition, only indels shorter than 15 bp were kept because longer ones could be due to intron retention, a known and extensive phenomenon in *E. huxelyi*^6^. The number of bases covered by reads in each strain was calculated using GenomeCoverageBed tool from BedTools (v 2.26.0)^44^. Upset plots were drawn using the R package ‘UpSetR’ (v 1.4.0)^45^.

For 18S analysis, reads were mapped to L04957.2, and manually inspected using IGV (v 2.8.12) (https://software.broadinstitute.org/software/igv/) after mapping and removing duplicates as described before. 18S sequences were downloaded from GenBank, see legend of Supplementary Fig. S4. Multiple alignment was performed with ClustalW (v 2.1)^46^.

### Quality control

The representative transcripts were blasted (BlastX)^47^ against the NCBI non-redundant protein database (nr), with the parameters -max_target_seqs 20 -evalue 0.001. In addition, BlastN against NCBI non-redundant nucleotide database (nt) was performed (-evalue 0.001) for all the transcripts to identify contamination. Transcripts whose best hit was from *Emiliania huxleyi* or the closely related species: *Seriola lalandi dorsalis, Chrysochromulina, Aureococcus* and G*uillardia theta* were left in the dataset. Transcripts matching any other species were processed with criteria for removal, either: (1) BlastX similarity of 95% or (2) BlastN identity of 95% with at least 50% of the length of our transcript in the match. In addition, (3) sequences below 350 bp that did not match any of the close species were removed. All representative transcripts that matched any one criterion were removed along with the putative transcript isoforms of the representative transcript.

The full transcriptome and representative transcripts were mapped to the Emiliana_huxleyi_CCMP1516_main_genome_assembly_v1.0 draft genome (ftp://ftp.ensemblgenomes.org/pub/protists/release-48/fasta/emiliania_huxleyi/dna/) using the minimap2 software^13^ with the option -ax splice. Minimap2 allows up to 15% mismatches by default.

The BUSCO software (v 3) was applied to test transcriptome completeness, comparing the unified transcriptome to 303 BUSCO groups defined as essential to eukaryotes (Eukaryota database)^17^.

### Transcriptome translation

Translation of the representative transcript per locus was performed using in-house Perl scripts and formulas in Excel files. Each transcript was translated in six frames to determine potential ORFs. For each transcript, the first three BlastX hits (same search as above, in Quality Control) were used to determine the frame and coordinates of the ORF to be translated. If there was a majority among the three top hits, the reading frame and coordinates of the matching query was used to designate the ORF from that transcript. When there was no majority, all three options were checked and the longest ORF that matched was chosen. If there were no BlastX hits, the longest ORF was chosen. A methionine was not required, unless there was a stop codon upstream of the potential ORF. When the potential protein was shorter than 50 amino acids, the transcript was not translated and it was defined as a potential non-coding gene, unless these were plastid proteins.

In addition, representative transcripts longer than 2000 bp, with the longest ORF spanning less than 80% of the length, were screened for potential chimeras. The three best potential ORFs per transcript were analyzed. The original transcripts were split into up to 3 new transcripts, if their ORFs did not overlap among themselves and the newly defined proteins had at least 300 amino acids.

### Quantification and differential expression

Mapping of the reads to the transcripts and transcript abundance was calculated using RSEM^20^ with the parameter—bowtie2. Differentially expressed transcripts were identified by the Bioconductor package DESeq2^48^. Expressed genes were required to have at least 7 normalized reads in at least 3 samples. Differentially expressed genes (fold change > 2 and FDR < 0.05 between any two conditions) were clustered into 8 clusters using the K-means algorithm. K-means clustering was performed using the Partek Genomics Suite® software, version 7.0, Partek Inc., St. Louis, MO, USA.

### Functional annotation

The representative transcripts were annotated using Gene Ontology (GO)^14,49^ terms by applying Blast2GO^15^. BlastX was run against nr (-max_target_seqs 20 -evalue 0.001) and the results imported into Blast2GO. The Annotation Cut-Off score in Blast2GO was set to 45.

KEGG annotation^50^ was performed at the DNA level through the KAAS web server (https://www.genome.jp/kegg/kaas/) and then translating between the KEGG orthologue numbers into pathways using the KEGG Mapper—Reconstruct Pathway server (https://www.genome.jp/kegg/tool/map_pathway.html).

### Functional enrichment

GO enrichment analysis was performed using Ontologizer 2.0^21^ (http://ontologizer.de) with weighted topology method^22^. Enrichment *p* values in other analyses were calculated by applying the hypergeometric test in R. Terms were summarized using Revigo^51^.

## Supplementary Information

Supplementary Figures.

Supplementary Table S1.

Supplementary Table S2.

Supplementary Table S3.

Supplementary Table S4.

Supplementary Table S5.

Supplementary Table S6.

## Data Availability

The raw sequence reads for the two previous transcriptomes unified in this paper are available from NCBI via accession numbers PRJNA184929 and PRJNA283462. The transcript and protein fasta files, vcf files of SNPs and indels, and GO and KEGG functional annotation files are available via the Figshare repository: 10.6084/m9.figshare.13794008.

## Code availability

In house scripts are available via the Figshare repository: 10.6084/m9.figshare.13795109.
