# Supplementary material for: An Emiliania huxleyi pan-transcriptome reveals basal strain specificity in gene expression patterns
Source: Sci Rep. 2021 Oct 21;11:20795. doi: 10.1038/s41598-021-00072-5 (PMC8531018; doi:10.1038/s41598-021-00072-5)
Supplement: Supplementary file 1 — Supplementary Figures. [file 41598_2021_72_MOESM1_ESM.docx]

# **An *Emiliania huxleyi* pan-transcriptome reveals basal strain specificity in gene expression patterns**

# Ester Feldmesser^1^, Shifra Ben-Dor^2^, Assaf Vardi^3^

# **SUPPLEMENTARY INFORMATION**

Table of Contents

**Supplementary Figure S1 2**

**Supplementary Figure S23**

**Supplementary Figure S34**

**Supplementary Figure S45**

**Supplementary Figure S522**

**Supplementary Figure S624**


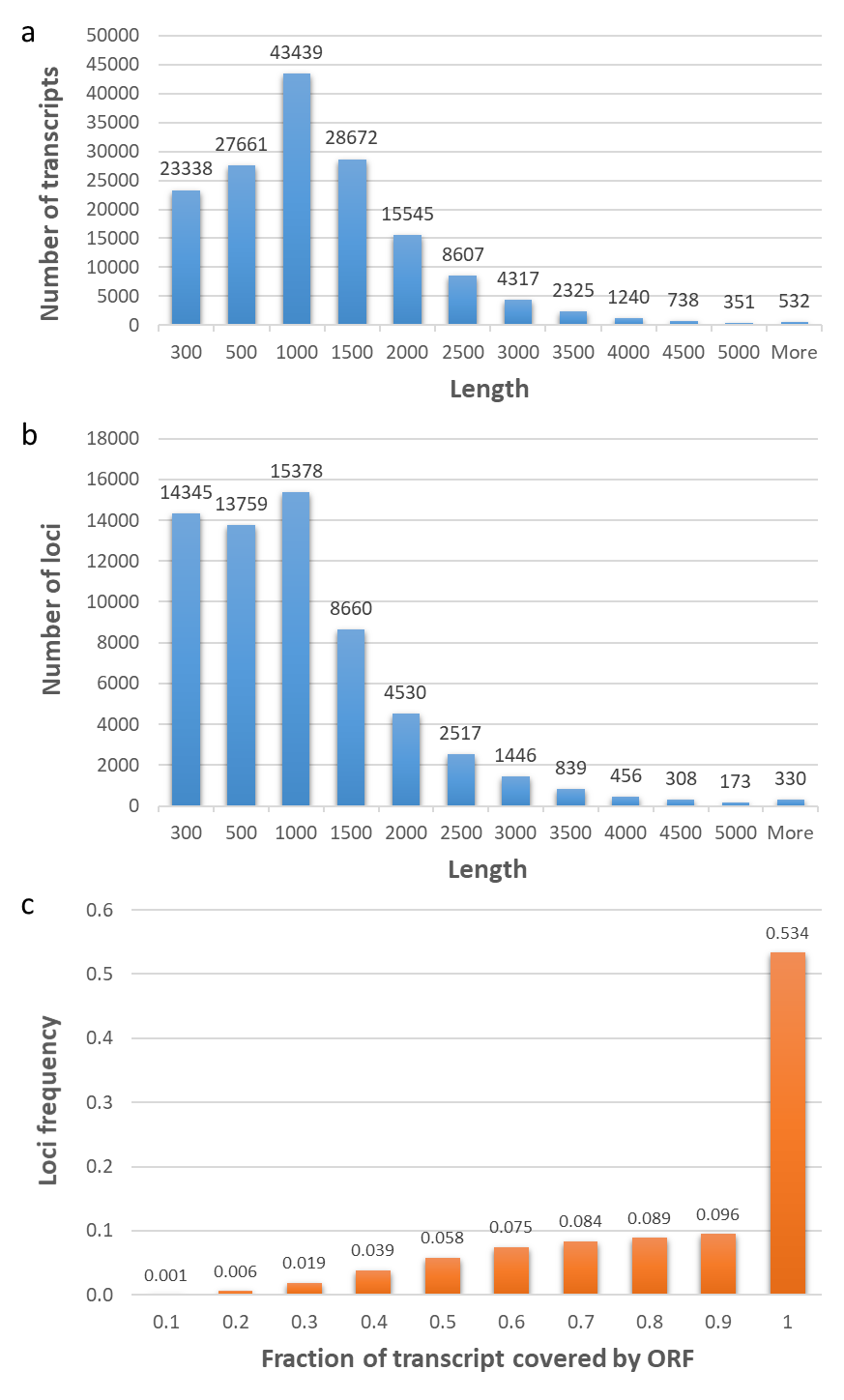


Supplementary Fig S1. Transcriptome characteristics. (a) Transcript length frequency. (b) Representative transcript (one per locus) length frequency. (c) Fraction of transcript covered by ORF. Numbers at the top of the bars represent the number of transcripts in each length bin in a and b, or fraction of transcript covered by ORF in c.


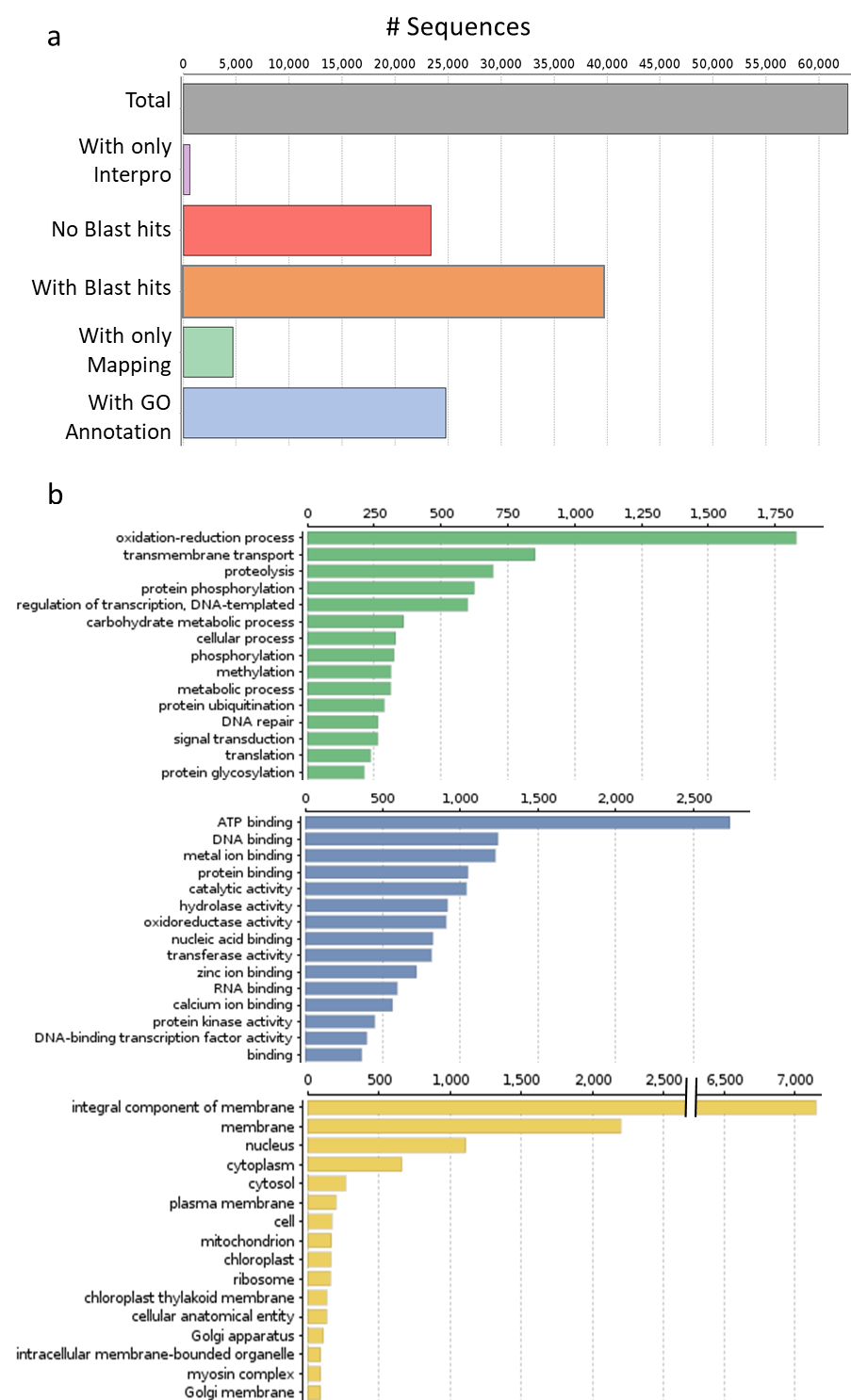


Supplementary Fig S2. Blast2GO annotation results (a) Blast2GO annotation summary (b) Blast2GO Gene Ontology categories. The scale bar represents the number of representative transcripts per 15 top annotations. Green indicates Biological Process, blue Molecular Function and yellow Cellular Component


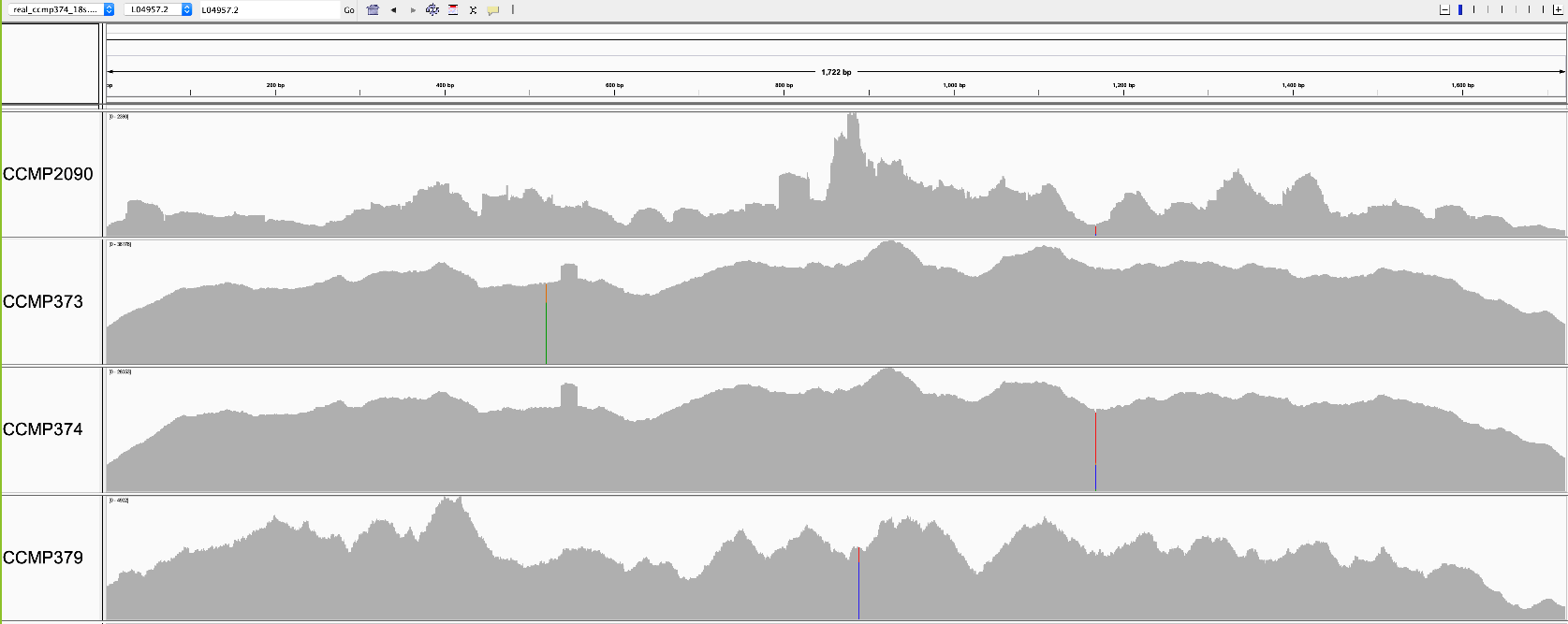


Supplementary Fig S3. IGV plot (coverage track) of reads from the four strains mapped to the CCMP374 18S RNA sequence (L04957.2)

CCMP374 --------------AGTCATATGCTTGTCTCAAAGATTAAGCCATGCATGTCTAAGTATA

920PML --------------AGTCATATGCTTGTCTCAAAGATTAAGCCATGCATGTCTAAGTATA

Ch25_90 --------------AGTCATATGCTTGTCTCAAAGATTAAGCCATGCATGTCTAAGTATA

CCAP920_9 TTGATCCTGCCAGTAGTCATATGCTTGTCTCAAAGATTAAGCCATGCATGTCTAAGTATA

UNC1419 --------------AGTCATATGCTTGTCTCAAAGATTAAGCCATGCATGTCTAAGTATA

RCC6856 ----------------------------------GATTAAGCCATGCATGTCTAAGTATA

RCC909 ------------------------------------------------------------

KMMCC_H-18 ------------------------------------------------------------

RCC1208 -------------TAGTCATATGCTTGTCTCAAAGAT-AAGCCATGCATGTCTAAGTATA

RCC1210 -------------TAGTCATATGCTTGTCTCAAAGAT-AAGCCATGCATGTCTAAGTATA

RCC1213 -------------TAGTCATATGCTTGTCTCAAAGAT-AAGCCATGCATGTCTAAGTATA

RCC1214 -------------TAGTCATATGCTTGTCTCAAAGAT-AAGCCATGCATGTCTAAGTATA

RCC1218 -------------TAGTCATATGCTTGTCTCAAAGAT-AAGCCATGCATGTCTAAGTATA

RCC1219 -------------TAGTCATATGCTTGTCTCAAAGAT-AAGCCATGCATGTCTAAGTATA

RCC1221 -------------TAGTCATATGCTTGTCTCAAAGAT-AAGCCATGCATGTCTAAGTATA

RCC1225 -------------TAGTCATATGCTTGTCTCAAAGAT-AAGCCATGCATGTCTAAGTATA

RCC1227 -------------TAGTCATATGCTTGTCTCAAAGAT-AAGCCATGCATGTCTAAGTATA

RCC1228 -------------TAGTCATATGCTTGTCTCAAAGAT-AAGCCATGCATGTCTAAGTATA

RCC1229 -------------TAGTCATATGCTTGTCTCAAAGAT-AAGCCATGCATGTCTAAGTATA

RCC1245 -------------TAGTCATATGCTTGTCTCAAAGAT-AAGCCATGCATGTCTAAGTATA

RCC1246 -------------TAGTCATATGCTTGTCTCAAAGAT-AAGCCATGCATGTCTAAGTATA

RCC1247 -------------TAGTCATATGCTTGTCTCAAAGAT-AAGCCATGCATGTCTAAGTATA

RCC1249 -------------TAGTCATATGCTTGTCTCAAAGAT-AAGCCATGCATGTCTAAGTATA

RCC1250 -------------TAGTCATATGCTTGTCTCAAAGAT-AAGCCATGCATGTCTAAGTATA

RCC1251 -------------TAGTCATATGCTTGTCTCAAAGAT-AAGCCATGCATGTCTAAGTATA

RCC1254 -------------TAGTCATATGCTTGTCTCAAAGAT-AAGCCATGCATGTCTAAGTATA

RCC1257 -------------TAGTCATATGCTTGTCTCAAAGAT-AAGCCATGCATGTCTAAGTATA

RCC1258 -------------TAGTCATATGCTTGTCTCAAAGAT-AAGCCATGCATGTCTAAGTATA

RCC1322 -------------TAGTCATATGCTTGTCTCAAAGAT-AAGCCATGCATGTCTAAGTATA

ESP7414 -------------TAGTCATATGCTTGTCTCAAAGAT-AAGCCATGCATGTCTAAGTATA

CCMP374 AGCGACTATACTGTGAAACTGCGAATGGCTCATTAAATCAGTTATGGTTTATTTGATGGT

920PML AGCGACTATACTGTGAAACTGCGAATGGCTCATTAAATCAGTTATGGTTTATTTGATGGT

Ch25_90 AGCGACTATACTGTGAAACTGCGAATGGCTCATTAAATCAGTTATGGTTTATTTGATGGT

CCAP920_9 AGCGACTATACTGTGAAACTGCGAATGGCTCATTAAATCAGTTATGGTTTATTTGATGGT

UNC1419 AGCGACTATACTGTGAAACTGCGAATGGCTCATTAAATCAGTTATGGTTTATTTGATGGT

RCC6856 AGCGACTATACTGTGAAACTGCGAATGGCTCATTAAATCAGTTATGGTTTATTTGATGGT

RCC909 ----------------AACTGCGAATGGCTCATTAAATCAGTTATGGTTTATTTGATGGT

KMMCC_H-18 --------------------------GGCTCATTAAATCAGTTATAGTTTATTTGATGGT

RCC1208 AGCGACTATACTGTGAAACTGCGAATGGCTCATTAAATCAGTTATGGTTTATTTGATGGT

RCC1210 AGCGACTATACTGTGAAACTGCGAATGGCTCATTAAATCAGTTATGGTTTATTTGATGGT

RCC1213 AGCGACTATACTGTGAAACTGCGAATGGCTCATTAAATCAGTTATGGTTTATTTGATGGT

RCC1214 AGCGACTATACTGTGAAACTGCGAATGGCTCATTAAATCAGTTATGGTTTATTTGATGGT

RCC1218 AGCGACTATACTGTGAAACTGCGAATGGCTCATTAAATCAGTTATGGTTTATTTGATGGT

RCC1219 AGCGACTATACTGTGAAACTGCGAATGGCTCATTAAATCAGTTATGGTTTATTTGATGGT

RCC1221 AGCGACTATACTGTGAAACTGCGAATGGCTCATTAAATCAGTTATGGTTTATTTGATGGT

RCC1225 AGCGACTATACTGTGAAACTGCGAATGGCTCATTAAATCAGTTATGGTTTATTTGATGGT

RCC1227 AGCGACTATACTGTGAAACTGCGAATGGCTCATTAAATCAGTTATGGTTTATTTGATGGT

RCC1228 AGCGACTATACTGTGAAACTGCGAATGGCTCATTAAATCAGTTATGGTTTATTTGATGGT

RCC1229 AGCGACTATACTGTGAAACTGCGAATGGCTCATTAAATCAGTTATGGTTTATTTGATGGT

RCC1245 AGCGACTATACTGTGAAACTGCGAATGGCTCATTAAATCAGTTATGGTTTATTTGATGGT

RCC1246 AGCGACTATACTGTGAAACTGCGAATGGCTCATTAAATCAGTTATGGTTTATTTGATGGT

RCC1247 AGCGACTATACTGTGAAACTGCGAATGGCTCATTAAATCAGTTATGGTTTATTTGATGGT

RCC1249 AGCGACTATACTGTGAAACTGCGAATGGCTCATTAAATCAGTTATGGTTTATTTGATGGT

RCC1250 AGCGACTATACTGTGAAACTGCGAATGGCTCATTAAATCAGTTATGGTTTATTTGATGGT

RCC1251 AGCGACTATACTGTGAAACTGCGAATGGCTCATTAAATCAGTTATGGTTTATTTGATGGT

RCC1254 AGCGACTATACTGTGAAACTGCGAATGGCTCATTAAATCAGTTATGGTTTATTTGATGGT

RCC1257 AGCGACTATACTGTGAAACTGCGAATGGCTCATTAAATCAGTTATGGTTTATTTGATGGT

RCC1258 AGCGACTATACTGTGAAACTGCGAATGGCTCATTAAATCAGTTATGGTTTATTTGATGGT

RCC1322 AGCGACTATACTGTGAAACTGCGAATGGCTCATTAAATCAGTTATGGTTTATTTGATGGT

ESP7414 AGCGACTATACTGTGAAACTGCGAATGGCTCATTAAATCAGTTATGGTTTATTTGATGGT

******************* **************

CCMP374 ACCTTGCTACTTGGATAACCGTAGTAATTCTAGAGCTAATACATGCAGGAGTTCCCGACT

920PML ACCTTGCTACTTGGATAACCGTAGTAATTCTAGAGCTAATACATGCAGGAGTTCCCGACT

Ch25_90 ACCTTGCTACTTGGATAACCGTAGTAATTCTAGAGCTAATACATGCAGGAGTTCCCGACT

CCAP920_9 ACCTTGCTACTTGGATAACCGTAGTAATTCTAGAGCTAATACATGCAGGAGTTCCCGACT

UNC1419 ACCTTGCTACTTGGATAACCGTAGTAATTCTAGAGCTAATACATGCAGGAGTTCCCGACT

RCC6856 ACCTTGCTACTTGGATAACCGTAGTAATTCTAGAGCTAATACATGCAGGAGTTCCCGACT

RCC909 ACCTTGCTACTTGGATAACCGTAGTAATTCTAGAGCTAATACATGCAGGAGTTCCCGACT

KMMCC_H-18 ACCTTGCTACTTGGATAACCGTAGTAATTCTAGAGCTAATACATGCAGGAGTTCCCGACT

RCC1208 ACCTTGCTACTTGGATAACCGTAGTAATTCTAGAGCTAATACATGCAGGAGTTCCCGACT

RCC1210 ACCTTGCTACTTGGATAACCGTAGTAATTCTAGAGCTAATACATGCAGGAGTTCCCGACT

RCC1213 ACCTTGCTACTTGGATAACCGTAGTAATTCTAGAGCTAATACATGCAGGAGTTCCCGACT

RCC1214 ACCTTGCTACTTGGATAACCGTAGTAATTCTAGAGCTAATACATGCAGGAGTTCCCGACT

RCC1218 ACCTTGCTACTTGGATAACCGTAGTAATTCTAGAGCTAATACATGCAGGAGTTCCCGACT

RCC1219 ACCTTGCTACTTGGATAACCGTAGTAATTCTAGAGCTAATACATGCAGGAGTTCCCGACT

RCC1221 ACCTTGCTACTTGGATAACCGTAGTAATTCTAGAGCTAATACATGCAGGAGTTCCCGACT

RCC1225 ACCTTGCTACTTGGATAACCGTAGTAATTCTAGAGCTAATACATGCAGGAGTTCCCGACT

RCC1227 ACCTTGCTACTTGGATAACCGTAGTAATTCTAGAGCTAATACATGCAGGAGTTCCCGACT

RCC1228 ACCTTGCTACTTGGATAACCGTAGTAATTCTAGAGCTAATACATGCAGGAGTTCCCGACT

RCC1229 ACCTTGCTACTTGGATAACCGTAGTAATTCTAGAGCTAATACATGCAGGAGTTCCCGACT

RCC1245 ACCTTGCTACTTGGATAACCGTAGTAATTCTAGAGCTAATACATGCAGGAGTTCCCGACT

RCC1246 ACCTTGCTACTTGGATAACCGTAGTAATTCTAGAGCTAATACATGCAGGAGTTCCCGACT

RCC1247 ACCTTGCTACTTGGATAACCGTAGTAATTCTAGAGCTAATACATGCAGGAGTTCCCGACT

RCC1249 ACCTTGCTACTTGGATAACCGTAGTAATTCTAGAGCTAATACATGCAGGAGTTCCCGACT

RCC1250 ACCTTGCTACTTGGATAACCGTAGTAATTCTAGAGCTAATACATGCAGGAGTTCCCGACT

RCC1251 ACCTTGCTACTTGGATAACCGTAGTAATTCTAGAGCTAATACATGCAGGAGTTCCCGACT

RCC1254 ACCTTGCTACTTGGATAACCGTAGTAATTCTAGAGCTAATACATGCAGGAGTTCCCGACT

RCC1257 ACCTTGCTACTTGGATAACCGTAGTAATTCTAGAGCTAATACATGCAGGAGTTCCCGACT

RCC1258 ACCTTGCTACTTGGATAACCGTAGTAATTCTAGAGCTAATACATGCAGGAGTTCCCGACT

RCC1322 ACCTTGCTACTTGGATAACCGTAGTAATTCTAGAGCTAATACATGCAGGAGTTCCCGACT

ESP7414 ACCTTGCTACTTGGATAACCGTAGTAATTCTAGAGCTAATACATGCAGGAGTTCCCGACT

************************************************************

CCMP374 CACGGAGGGATGTATTTATTAGATAAGAAACCAAACCGGTCTCCGGTTGCGTGCTGAGTC

920PML CACGGAGGGATGTATTTATTAGATAAGAAACCAAACCGGTCTCCGGTTGCGTGCTGAGTC

Ch25_90 CACGGAGGGATGTATTTATTAGATAAGAAACCAAACCGGTCTCCGGTTGCGTGCTGAGTC

CCAP920_9 CACGGAGGGATGTATTTATTAGATAAGAAACCAAACCGGTCTCCGGTTGCGTGCTGAGTC

UNC1419 CACGGAGGGATGTATTTATTAGATAAGAAACCAAACCGGTCTCCGGTTGCGTGCTGAGTC

RCC6856 CACGGAGGGATGTATTTATTAGATAAGAAACCAAACCGGTCTCCGGTTGCGTGCTGAGTC

RCC909 CACGGAGGGATGTATTTATTAGATAAGAAACCAAACCGGTCTCCGGTTGCGTGCTGAGTC

KMMCC_H-18 CACGGAGGGATGTATTTATTAGATAAGAAACCAAACCGGTCTCCGGTTGCGTGCTGAGTC

RCC1208 CACGGAGGGATGTATTTATTAGATAAGAAACCAAACCGGTCTCCGGTTGCGTGCTGAGTC

RCC1210 CACGGAGGGATGTATTTATTAGATAAGAAACCAAACCGGTCTCCGGTTGCGTGCTGAGTC

RCC1213 CACGGAGGGATGTATTTATTAGATAAGAAACCAAACCGGTCTCCGGTTGCGTGCTGAGTC

RCC1214 CACGGAGGGATGTATTTATTAGATAAGAAACCAAACCGGTCTCCGGTTGCGTGCTGAGTC

RCC1218 CACGGAGGGATGTATTTATTAGATAAGAAACCAAACCGGTCTCCGGTTGCGTGCTGAGTC

RCC1219 CACGGAGGGATGTATTTATTAGATAAGAAACCAAACCGGTCTCCGGTTGCGTGCTGAGTC

RCC1221 CACGGAGGGATGTATTTATTAGATAAGAAACCAAACCGGTCTCCGGTTGCGTGCTGAGTC

RCC1225 CACGGAGGGATGTATTTATTAGATAAGAAACCAAACCGGTCTCCGGTTGCGTGCTGAGTC

RCC1227 CACGGAGGGATGTATTTATTAGATAAGAAACCAAACCGGTCTCCGGTTGCGTGCTGAGTC

RCC1228 CACGGAGGGATGTATTTATTAGATAAGAAACCAAACCGGTCTCCGGTTGCGTGCTGAGTC

RCC1229 CACGGAGGGATGTATTTATTAGATAAGAAACCAAACCGGTCTCCGGTTGCGTGCTGAGTC

RCC1245 CACGGAGGGATGTATTTATTAGATAAGAAACCAAACCGGTCTCCGGTTGCGTGCTGAGTC

RCC1246 CACGGAGGGATGTATTTATTAGATAAGAAACCAAACCGGTCTCCGGTTGCGTGCTGAGTC

RCC1247 CACGGAGGGATGTATTTATTAGATAAGAAACCAAACCGGTCTCCGGTTGCGTGCTGAGTC

RCC1249 CACGGAGGGATGTATTTATTAGATAAGAAACCAAACCGGTCTCCGGTTGCGTGCTGAGTC

RCC1250 CACGGAGGGATGTATTTATTAGATAAGAAACCAAACCGGTCTCCGGTTGCGTGCTGAGTC

RCC1251 CACGGAGGGATGTATTTATTAGATAAGAAACCAAACCGGTCTCCGGTTGCGTGCTGAGTC

RCC1254 CACGGAGGGATGTATTTATTAGATAAGAAACCAAACCGGTCTCCGGTTGCGTGCTGAGTC

RCC1257 CACGGAGGGATGTATTTATTAGATAAGAAACCAAACCGGTCTCCGGTTGCGTGCTGAGTC

RCC1258 CACGGAGGGATGTATTTATTAGATAAGAAACCAAACCGGTCTCCGGTTGCGTGCTGAGTC

RCC1322 CACGGAGGGATGTATTTATTAGATAAGAAACCAAACCGGTCTCCGGTTGCGTGCTGAGTC

ESP7414 CACGGAGGGATGTATTTATTAGATAAGAAACCAAACCGGTCTCCGGTTGCGTGCTGAGTC

************************************************************

CCMP374 ATAATAACTGCTCGAATCGCACGGCTCTACGCCGGCGATGGTTCATTCAAATTTCTGCCC

920PML ATAATAACTGCTCGAATCGCACGGCTCTACGCCGGCGATGGTTCATTCAAATTTCTGCCC

Ch25_90 ATAATAACTGCTCGAATCGCACGGCTCTACGCCGGCGATGGTTCATTCAAATTTCTGCCC

CCAP920_9 ATAATAACTGCTCGAATCGCACGGCTCTACGCCGGCGATGGTTCATTCAAATTTCTGCCC

UNC1419 ATAATAACTGCTCGAATCGCACGGCTCTACGCCGGCGATGGTTCATTCAAATTTCTGCCC

RCC6856 ATAATAACTGCTCGAATCGCACGGCTCTACGCCGGCGATGGTTCATTCAAATTTCTGCCC

RCC909 ATAATAACTGCTCGAATCGCACGGCTCTACGCCGGCGATGGTTCATTCAAATTTCTGCCC

KMMCC_H-18 ATAATAACTGCTCGAATCGCACGGCTCTACGCCGGCGATGGTTCATTCAAATTTCTGCCC

RCC1208 ATAATAACTGCTCGAATCGCACGGCTCTACGCCGGCGATGGTTCATTCAAATTTCTGCCC

RCC1210 ATAATAACTGCTCGAATCGCACGGCTCTACGCCGGCGATGGTTCATTCAAATTTCTGCCC

RCC1213 ATAATAACTGCTCGAATCGCACGGCTCTACGCCGGCGATGGTTCATTCAAATTTCTGCCC

RCC1214 ATAATAACTGCTCGAATCGCACGGCTCTACGCCGGCGATGGTTCATTCAAATTTCTGCCC

RCC1218 ATAATAACTGCTCGAATCGCACGGCTCTACGCCGGCGATGGTTCATTCAAATTTCTGCCC

RCC1219 ATAATAACTGCTCGAATCGCACGGCTCTACGCCGGCGATGGTTCATTCAAATTTCTGCCC

RCC1221 ATAATAACTGCTCGAATCGCACGGCTCTACGCCGGCGATGGTTCATTCAAATTTCTGCCC

RCC1225 ATAATAACTGCTCGAATCGCACGGCTCTACGCCGGCGATGGTTCATTCAAATTTCTGCCC

RCC1227 ATAATAACTGCTCGAATCGCACGGCTCTACGCCGGCGATGGTTCATTCAAATTTCTGCCC

RCC1228 ATAATAACTGCTCGAATCGCACGGCTCTACGCCGGCGATGGTTCATTCAAATTTCTGCCC

RCC1229 ATAATAACTGCTCGAATCGCACGGCTCTACGCCGGCGATGGTTCATTCAAATTTCTGCCC

RCC1245 ATAATAACTGCTCGAATCGCACGGCTCTACGCCGGCGATGGTTCATTCAAATTTCTGCCC

RCC1246 ATAATAACTGCTCGAATCGCACGGCTCTACGCCGGCGATGGTTCATTCAAATTTCTGCCC

RCC1247 ATAATAACTGCTCGAATCGCACGGCTCTACGCCGGCGATGGTTCATTCAAATTTCTGCCC

RCC1249 ATAATAACTGCTCGAATCGCACGGCTCTACGCCGGCGATGGTTCATTCAAATTTCTGCCC

RCC1250 ATAATAACTGCTCGAATCGCACGGCTCTACGCCGGCGATGGTTCATTCAAATTTCTGCCC

RCC1251 ATAATAACTGCTCGAATCGCACGGCTCTACGCCGGCGATGGTTCATTCAAATTTCTGCCC

RCC1254 ATAATAACTGCTCGAATCGCACGGCTCTACGCCGGCGATGGTTCATTCAAATTTCTGCCC

RCC1257 ATAATAACTGCTCGAATCGCACGGCTCTACGCCGGCGATGGTTCATTCAAATTTCTGCCC

RCC1258 ATAATAACTGCTCGAATCGCACGGCTCTACGCCGGCGATGGTTCATTCAAATTTCTGCCC

RCC1322 ATAATAACTGCTCGAATCGCACGGCTCTACGCCGGCGATGGTTCATTCAAATTTCTGCCC

ESP7414 ATAATAACTGCTCGAATCGCACGGCTCTACGCCGGCGATGGTTCATTCAAATTTCTGCCC

************************************************************

CCMP374 TATCAGCTTTCGATGGTAGGATAGAGGCCTACCATGGCGTTAACGGGTAACGGAGAATTA

920PML TATCAGCTTTCGATGGTAGGATAGAGGCCTACCATGGCGTTAACGGGTAACGGAGAATTA

Ch25_90 TATCAGCTTTCGATGGTAGGATAGAGGCCTACCATGGCGTTAACGGGTAACGGAGAATTA

CCAP920_9 TATCAGCTTTCGATGGTAGGATAGAGGCCTACCATGGCGTTAACGGGTAACGGAGAATTA

UNC1419 TATCAGCTTTCGATGGTAGGATAGAGGCCTACCATGGCGTTAACGGGTAACGGAGAATTA

RCC6856 TATCAGCTTTCGATGGTAGGATAGAGGCCTACCATGGCGTTAACGGGTAACGGAGAATTA

RCC909 TATCAGCTTTCGATGGTAGGATAGAGGCCTACCATGGCGTTAACGGGTAACGGAGAATTA

KMMCC_H-18 TATCAGCTTTCGATGGTAGGATAGAGGCCTACCATGGCGTTAACGGGTAACGGAGAATTA

RCC1208 TATCAGCTTTCGATGGTAGGATAGAGGCCTACCATGGCGTTAACGGGTAACGGAGAATTA

RCC1210 TATCAGCTTTCGATGGTAGGATAGAGGCCTACCATGGCGTTAACGGGTAACGGAGAATTA

RCC1213 TATCAGCTTTCGATGGTAGGATAGAGGCCTACCATGGCGTTAACGGGTAACGGAGAATTA

RCC1214 TATCAGCTTTCGATGGTAGGATAGAGGCCTACCATGGCGTTAACGGGTAACGGAGAATTA

RCC1218 TATCAGCTTTCGATGGTAGGATAGAGGCCTACCATGGCGTTAACGGGTAACGGAGAATTA

RCC1219 TATCAGCTTTCGATGGTAGGATAGAGGCCTACCATGGCGTTAACGGGTAACGGAGAATTA

RCC1221 TATCAGCTTTCGATGGTAGGATAGAGGCCTACCATGGCGTTAACGGGTAACGGAGAATTA

RCC1225 TATCAGCTTTCGATGGTAGGATAGAGGCCTACCATGGCGTTAACGGGTAACGGAGAATTA

RCC1227 TATCAGCTTTCGATGGTAGGATAGAGGCCTACCATGGCGTTAACGGGTAACGGAGAATTA

RCC1228 TATCAGCTTTCGATGGTAGGATAGAGGCCTACCATGGCGTTAACGGGTAACGGAGAATTA

RCC1229 TATCAGCTTTCGATGGTAGGATAGAGGCCTACCATGGCGTTAACGGGTAACGGAGAATTA

RCC1245 TATCAGCTTTCGATGGTAGGATAGAGGCCTACCATGGCGTTAACGGGTAACGGAGAATTA

RCC1246 TATCAGCTTTCGATGGTAGGATAGAGGCCTACCATGGCGTTAACGGGTAACGGAGAATTA

RCC1247 TATCAGCTTTCGATGGTAGGATAGAGGCCTACCATGGCGTTAACGGGTAACGGAGAATTA

RCC1249 TATCAGCTTTCGATGGTAGGATAGAGGCCTACCATGGCGTTAACGGGTAACGGAGAATTA

RCC1250 TATCAGCTTTCGATGGTAGGATAGAGGCCTACCATGGCGTTAACGGGTAACGGAGAATTA

RCC1251 TATCAGCTTTCGATGGTAGGATAGAGGCCTACCATGGCGTTAACGGGTAACGGAGAATTA

RCC1254 TATCAGCTTTCGATGGTAGGATAGAGGCCTACCATGGCGTTAACGGGTAACGGAGAATTA

RCC1257 TATCAGCTTTCGATGGTAGGATAGAGGCCTACCATGGCGTTAACGGGTAACGGAGAATTA

RCC1258 TATCAGCTTTCGATGGTAGGATAGAGGCCTACCATGGCGTTAACGGGTAACGGAGAATTA

RCC1322 TATCAGCTTTCGATGGTAGGATAGAGGCCTACCATGGCGTTAACGGGTAACGGAGAATTA

ESP7414 TATCAGCTTTCGATGGTAGGATAGAGGCCTACCATGGCGTTAACGGGTAACGGAGAATTA

************************************************************

CCMP374 GGGTTCGATTCCGGAGAGGGAGCCTGAGAAATGGCTACCACATCCAAGGAAGGCAGCAGG

920PML GGGTTCGATTCCGGAGAGGGAGCCTGAGAAATGGCTACCACATCCAAGGAAGGCAGCAGG

Ch25_90 GGGTTCGATTCCGGAGAGGGAGCCTGAGAAATGGCTACCACATCCAAGGAAGGCAGCAGG

CCAP920_9 GGGTTCGATTCCGGAGAGGGAGCCTGAGAAATGGCTACCACATCCAAGGAAGGCAGCAGG

UNC1419 GGGTTCGATTCCGGAGAGGGAGCCTGAGAAATGGCTACCACATCCAAGGAAGGCAGCAGG

RCC6856 GGGTTCGATTCCGGAGAGGGAGCCTGAGAAATGGCTACCACATCCAAGGAAGGCAGCAGG

RCC909 GGGTTCGATTCCGGAGAGGGAGCCTGAGAAATGGCTACCACATCCAAGGAAGGCAGCAGG

KMMCC_H-18 GGGTTCGATTCCGGAGAGGGAGCCTGAGAAATGGCTACCACATCCAAGGAAGGCAGCAGG

RCC1208 GGGTTCGATTCCGGAGAGGGAGCCTGAGAAATGGCTACCACATCCAAGGAAGGCAGCAGG

RCC1210 GGGTTCGATTCCGGAGAGGGAGCCTGAGAAATGGCTACCACATCCAAGGAAGGCAGCAGG

RCC1213 GGGTTCGATTCCGGAGAGGGAGCCTGAGAAATGGCTACCACATCCAAGGAAGGCAGCAGG

RCC1214 GGGTTCGATTCCGGAGAGGGAGCCTGAGAAATGGCTACCACATCCAAGGAAGGCAGCAGG

RCC1218 GGGTTCGATTCCGGAGAGGGAGCCTGAGAAATGGCTACCACATCCAAGGAAGGCAGCAGG

RCC1219 GGGTTCGATTCCGGAGAGGGAGCCTGAGAAATGGCTACCACATCCAAGGAAGGCAGCAGG

RCC1221 GGGTTCGATTCCGGAGAGGGAGCCTGAGAAATGGCTACCACATCCAAGGAAGGCAGCAGG

RCC1225 GGGTTCGATTCCGGAGAGGGAGCCTGAGAAATGGCTACCACATCCAAGGAAGGCAGCAGG

RCC1227 GGGTTCGATTCCGGAGAGGGAGCCTGAGAAATGGCTACCACATCCAAGGAAGGCAGCAGG

RCC1228 GGGTTCGATTCCGGAGAGGGAGCCTGAGAAATGGCTACCACATCCAAGGAAGGCAGCAGG

RCC1229 GGGTTCGATTCCGGAGAGGGAGCCTGAGAAATGGCTACCACATCCAAGGAAGGCAGCAGG

RCC1245 GGGTTCGATTCCGGAGAGGGAGCCTGAGAAATGGCTACCACATCCAAGGAAGGCAGCAGG

RCC1246 GGGTTCGATTCCGGAGAGGGAGCCTGAGAAATGGCTACCACATCCAAGGAAGGCAGCAGG

RCC1247 GGGTTCGATTCCGGAGAGGGAGCCTGAGAAATGGCTACCACATCCAAGGAAGGCAGCAGG

RCC1249 GGGTTCGATTCCGGAGAGGGAGCCTGAGAAATGGCTACCACATCCAAGGAAGGCAGCAGG

RCC1250 GGGTTCGATTCCGGAGAGGGAGCCTGAGAAATGGCTACCACATCCAAGGAAGGCAGCAGG

RCC1251 GGGTTCGATTCCGGAGAGGGAGCCTGAGAAATGGCTACCACATCCAAGGAAGGCAGCAGG

RCC1254 GGGTTCGATTCCGGAGAGGGAGCCTGAGAAATGGCTACCACATCCAAGGAAGGCAGCAGG

RCC1257 GGGTTCGATTCCGGAGAGGGAGCCTGAGAAATGGCTACCACATCCAAGGAAGGCAGCAGG

RCC1258 GGGTTCGATTCCGGAGAGGGAGCCTGAGAAATGGCTACCACATCCAAGGAAGGCAGCAGG

RCC1322 GGGTTCGATTCCGGAGAGGGAGCCTGAGAAATGGCTACCACATCCAAGGAAGGCAGCAGG

ESP7414 GGGTTCGATTCCGGAGAGGGAGCCTGAGAAATGGCTACCACATCCAAGGAAGGCAGCAGG

************************************************************

CCMP374 CGCGTAAATTGCCCGAATCCTGACACAGGGAGGTAGTGACAAGAAATAACAATACAGGGC

920PML CGCGTAAATTGCCCGAATCCTGACACAGGGAGGTAGTGACAAGAAATAACAATACAGGGC

Ch25_90 CGCGTAAATTGCCCGAATCCTGACACAGGGAGGTAGTGACAAGAAATAACAATACAGGGC

CCAP920_9 CGCGTAAATTGCCCGAATCCTGACACAGGGAGGTAGTGACAAGAAATAACAATACAGGGC

UNC1419 CGCGTAAATTGCCCGAATCCTGACACAGGGAGGTAGTGACAAGAAATAACAATACAGGGC

RCC6856 CGCGTAAATTGCCCGAATCCTGACACAGGGAGGTAGTGACAAGAAATAACAATACAGGGC

RCC909 CGCGTAAATTGCCCGAATCCTGACACAGGGAGGTAGTGACAAGAAATAACAATACAGGGC

KMMCC_H-18 CGCGTAAATTGCCCGAATCCTGACACAGGGAGGTAGTGACAAGAAATAACAATACAGGGC

RCC1208 CGCGTAAATTGCCCGAATCCTGACACAGGGAGGTAGTGACAAGAAATAACAATACAGGGC

RCC1210 CGCGTAAATTGCCCGAATCCTGACACAGGGAGGTAGTGACAAGAAATAACAATACAGGGC

RCC1213 CGCGTAAATTGCCCGAATCCTGACACAGGGAGGTAGTGACAAGAAATAACAATACAGGGC

RCC1214 CGCGTAAATTGCCCGAATCCTGACACAGGGAGGTAGTGACAAGAAATAACAATACAGGGC

RCC1218 CGCGTAAATTGCCCGAATCCTGACACAGGGAGGTAGTGACAAGAAATAACAATACAGGGC

RCC1219 CGCGTAAATTGCCCGAATCCTGACACAGGGAGGTAGTGACAAGAAATAACAATACAGGGC

RCC1221 CGCGTAAATTGCCCGAATCCTGACACAGGGAGGTAGTGACAAGAAATAACAATACAGGGC

RCC1225 CGCGTAAATTGCCCGAATCCTGACACAGGGAGGTAGTGACAAGAAATAACAATACAGGGC

RCC1227 CGCGTAAATTGCCCGAATCCTGACACAGGGAGGTAGTGACAAGAAATAACAATACAGGGC

RCC1228 CGCGTAAATTGCCCGAATCCTGACACAGGGAGGTAGTGACAAGAAATAACAATACAGGGC

RCC1229 CGCGTAAATTGCCCGAATCCTGACACAGGGAGGTAGTGACAAGAAATAACAATACAGGGC

RCC1245 CGCGTAAATTGCCCGAATCCTGACACAGGGAGGTAGTGACAAGAAATAACAATACAGGGC

RCC1246 CGCGTAAATTGCCCGAATCCTGACACAGGGAGGTAGTGACAAGAAATAACAATACAGGGC

RCC1247 CGCGTAAATTGCCCGAATCCTGACACAGGGAGGTAGTGACAAGAAATAACAATACAGGGC

RCC1249 CGCGTAAATTGCCCGAATCCTGACACAGGGAGGTAGTGACAAGAAATAACAATACAGGGC

RCC1250 CGCGTAAATTGCCCGAATCCTGACACAGGGAGGTAGTGACAAGAAATAACAATACAGGGC

RCC1251 CGCGTAAATTGCCCGAATCCTGACACAGGGAGGTAGTGACAAGAAATAACAATACAGGGC

RCC1254 CGCGTAAATTGCCCGAATCCTGACACAGGGAGGTAGTGACAAGAAATAACAATACAGGGC

RCC1257 CGCGTAAATTGCCCGAATCCTGACACAGGGAGGTAGTGACAAGAAATAACAATACAGGGC

RCC1258 CGCGTAAATTGCCCGAATCCTGACACAGGGAGGTAGTGACAAGAAATAACAATACAGGGC

RCC1322 CGCGTAAATTGCCCGAATCCTGACACAGGGAGGTAGTGACAAGAAATAACAATACAGGGC

ESP7414 CGCGTAAATTGCCCGAATCCTGACACAGGGAGGTAGTGACAAGAAATAACAATACAGGGC

************************************************************

CCMP374 TATTTTAGTCTTGTAATTGGAATGAGTACAATTTACATCTCTTCACGAGGATCAATTGGA

920PML TATTTTAGTCTTGTAATTGGAATGAGTACAATTTACATCTCTTCACGAGGATCAATTGGA

Ch25_90 TATTTTAGTCTTGTAATTGGAATGAGTACAATTTACATCTCTTCACGAGGATCAATTGGA

CCAP920_9 TATTTTAGTCTTGTAATTGGAATGAGTACAATTTACATCTCTTCACGAGGATCAATTGGA

UNC1419 TATTTTAGTCTTGTAATTGGAATGAGTACAATTTACATCTCTTCACGAGGATCAATTGGA

RCC6856 TATTTTAGTCTTGTAATTGGAATGAGTACAATTTACATCTCTTCACGAGGATCAATTGGA

RCC909 TATTTTAGTCTTGTAATTGGAATGAGTACAATTTACATCTCTTCACGAGGATCAATTGGA

KMMCC_H-18 TATTTTAGTCTTGTAATTGGAATGAGTACAATTTACATCTCTTCACGAGGATCAATTGGA

RCC1208 TATTTTAGTCTTGTAATTGGAATGAGTACAATTTACATCTCTTCACGAGGATCAATTGGA

RCC1210 TATTTTAGTCTTGTAATTGGAATGAGTACAATTTACATCTCTTCACGAGGATCAATTGGA

RCC1213 TATTTTAGTCTTGTAATTGGAATGAGTACAATTTACATCTCTTCACGAGGATCAATTGGA

RCC1214 TATTTTAGTCTTGTAATTGGAATGAGTACAATTTACATCTCTTCACGAGGATCAATTGGA

RCC1218 TATTTTAGTCTTGTAATTGGAATGAGTACAATTTACATCTCTTCACGAGGATCAATTGGA

RCC1219 TATTTTAGTCTTGTAATTGGAATGAGTACAATTTACATCTCTTCACGAGGATCAATTGGA

RCC1221 TATTTTAGTCTTGTAATTGGAATGAGTACAATTTACATCTCTTCACGAGGATCAATTGGA

RCC1225 TATTTTAGTCTTGTAATTGGAATGAGTACAATTTACATCTCTTCACGAGGATCAATTGGA

RCC1227 TATTTTAGTCTTGTAATTGGAATGAGTACAATTTACATCTCTTCACGAGGATCAATTGGA

RCC1228 TATTTTAGTCTTGTAATTGGAATGAGTACAATTTACATCTCTTCACGAGGATCAATTGGA

RCC1229 TATTTTAGTCTTGTAATTGGAATGAGTACAATTTACATCTCTTCACGAGGATCAATTGGA

RCC1245 TATTTTAGTCTTGTAATTGGAATGAGTACAATTTACATCTCTTCACGAGGATCAATTGGA

RCC1246 TATTTTAGTCTTGTAATTGGAATGAGTACAATTTACATCTCTTCACGAGGATCAATTGGA

RCC1247 TATTTTAGTCTTGTAATTGGAATGAGTACAATTTACATCTCTTCACGAGGATCAATTGGA

RCC1249 TATTTTAGTCTTGTAATTGGAATGAGTACAATTTACATCTCTTCACGAGGATCAATTGGA

RCC1250 TATTTTAGTCTTGTAATTGGAATGAGTACAATTTACATCTCTTCACGAGGATCAATTGGA

RCC1251 TATTTTAGTCTTGTAATTGGAATGAGTACAATTTACATCTCTTCACGAGGATCAATTGGA

RCC1254 TATTTTAGTCTTGTAATTGGAATGAGTACAATTTACATCTCTTCACGAGGATCAATTGGA

RCC1257 TATTTTAGTCTTGTAATTGGAATGAGTACAATTTACATCTCTTCACGAGGATCAATTGGA

RCC1258 TATTTTAGTCTTGTAATTGGAATGAGTACAATTTACATCTCTTCACGAGGATCAATTGGA

RCC1322 TATTTTAGTCTTGTAATTGGAATGAGTACAATTTACATCTCTTCACGAGGATCAATTGGA

ESP7414 TATTTTAGTCTTGTAATTGGAATGAGTACAATTTACATCTCTTCACGAGGATCAATTGGA

************************************************************

CCMP374 GGGCAAGTCTGGTGCCAGCAGCCGCGGTAATTCCAGCTCCAATAGCGTATATTAAAGTTG

920PML GGGCAAGTCTGGTGCCAGCAGCCGCGGTAATTCCAGCTCCAATAGCGTATATTAAAGTTG

Ch25_90 GGGCAAGTCTGGTGCCAGCAGCCGCGGTAATTCCAGCTCCAATAGCGTATATTAAAGTTG

CCAP920_9 GGGCAAGTCTGGTGCCAGCAGCCGCGGTAATTCCAGCTCCAATAGCGTATATTAAAGTTG

UNC1419 GGGCAAGTCTGGTGCCAGCAGCCGCGGTAATTCCAGCTCCAATAGCGTATATTAAAGTTG

RCC6856 GGGCAAGTCTGGTGCCAGCAGCCGCGGTAATTCCAGCTCCAATAGCGTATATTAAAGTTG

RCC909 GGGCAAGTCTGGTGCCAGCAGCCGCGGTAATTCCAGCTCCAATAGCGTATATTAAAGTTG

KMMCC_H-18 GGGCAAGTCTGGTGCCAGCAGCCGCGGTAATTCCAGCTCCAATAGCGTATATTAAAGTTG

RCC1208 GGGCAAGTCTGGTGCCAGCAGCCGCGGTAATTCCAGCTCCAATAGCGTATATTAAAGTTG

RCC1210 GGGCAAGTCTGGTGCCAGCAGCCGCGGTAATTCCAGCTCCAATAGCGTATATTAAAGTTG

RCC1213 GGGCAAGTCTGGTGCCAGCAGCCGCGGTAATTCCAGCTCCAATAGCGTATATTAAAGTTG

RCC1214 GGGCAAGTCTGGTGCCAGCAGCCGCGGTAATTCCAGCTCCAATAGCGTATATTAAAGTTG

RCC1218 GGGCAAGTCTGGTGCCAGCAGCCGCGGTAATTCCAGCTCCAATAGCGTATATTAAAGTTG

RCC1219 GGGCAAGTCTGGTGCCAGCAGCCGCGGTAATTCCAGCTCCAATAGCGTATATTAAAGTTG

RCC1221 GGGCAAGTCTGGTGCCAGCAGCCGCGGTAATTCCAGCTCCAATAGCGTATATTAAAGTTG

RCC1225 GGGCAAGTCTGGTGCCAGCAGCCGCGGTAATTCCAGCTCCAATAGCGTATATTAAAGTTG

RCC1227 GGGCAAGTCTGGTGCCAGCAGCCGCGGTAATTCCAGCTCCAATAGCGTATATTAAAGTTG

RCC1228 GGGCAAGTCTGGTGCCAGCAGCCGCGGTAATTCCAGCTCCAATAGCGTATATTAAAGTTG

RCC1229 GGGCAAGTCTGGTGCCAGCAGCCGCGGTAATTCCAGCTCCAATAGCGTATATTAAAGTTG

RCC1245 GGGCAAGTCTGGTGCCAGCAGCCGCGGTAATTCCAGCTCCAATAGCGTATATTAAAGTTG

RCC1246 GGGCAAGTCTGGTGCCAGCAGCCGCGGTAATTCCAGCTCCAATAGCGTATATTAAAGTTG

RCC1247 GGGCAAGTCTGGTGCCAGCAGCCGCGGTAATTCCAGCTCCAATAGCGTATATTAAAGTTG

RCC1249 GGGCAAGTCTGGTGCCAGCAGCCGCGGTAATTCCAGCTCCAATAGCGTATATTAAAGTTG

RCC1250 GGGCAAGTCTGGTGCCAGCAGCCGCGGTAATTCCAGCTCCAATAGCGTATATTAAAGTTG

RCC1251 GGGCAAGTCTGGTGCCAGCAGCCGCGGTAATTCCAGCTCCAATAGCGTATATTAAAGTTG

RCC1254 GGGCAAGTCTGGTGCCAGCAGCCGCGGTAATTCCAGCTCCAATAGCGTATATTAAAGTTG

RCC1257 GGGCAAGTCTGGTGCCAGCAGCCGCGGTAATTCCAGCTCCAATAGCGTATATTAAAGTTG

RCC1258 GGGCAAGTCTGGTGCCAGCAGCCGCGGTAATTCCAGCTCCAATAGCGTATATTAAAGTTG

RCC1322 GGGCAAGTCTGGTGCCAGCAGCCGCGGTAATTCCAGCTCCAATAGCGTATATTAAAGTTG

ESP7414 GGGCAAGTCTGGTGCCAGCAGCCGCGGTAATTCCAGCTCCAATAGCGTATATTAAAGTTG

************************************************************

CCMP374 TTGCAGTTAAAACGCTCGTAGTCGGATTTCGGGGCGGGCCGACCGGTCTGCCGATGGGTA

920PML TTGCAGTTAAAACGCTCGTAGTCGGATTTCGGGGCGGGCCGACCGGTCTGCCGATGGGTA

Ch25_90 TTGCAGTTAAAACGCTCGTAGTCGGATTTCGGGGCGGGCCGACCGGTCTGCCGATGGGTA

CCAP920_9 TTGCAGTTAAAACGCTCGTAGTCGGATTTCGGGGCGGGCCGACCGGTCTGCCGATGGGTA

UNC1419 TTGCAGTTAAAACGCTCGTAGTCGGATTTCGGGGCGGGCCGACCGGTCTGCCGATGGGTA

RCC6856 TTGCAGTTAAAACGCTCGTAGTCGGATTTCGGGGCGGGCCGACCGGTCTGCCGATGGGTA

RCC909 TTGCAGTTAAAACGCTCGTAGTCGGATTTCGGGGCGGGCCGACCGGTCTGCCGATGGGTA

KMMCC_H-18 TTGCAGTTAAAACGCTCGTAGTCGGATTTCGGGGCGGGCCGACCGGTCTGCCGATGGGTA

RCC1208 TTGCAGTTAAAACGCTCGTAGTCGGATTTCGGGGCGGGCCGACCGGTCTGCCGATGGGTA

RCC1210 TTGCAGTTAAAACGCTCGTAGTCGGATTTCGGGGCGGGCCGACCGGTCTGCCGATGGGTA

RCC1213 TTGCAGTTAAAACGCTCGTAGTCGGATTTCGGGGCGGGCCGACCGGTCTGCCGATGGGTA

RCC1214 TTGCAGTTAAAACGCTCGTAGTCGGATTTCGGGGCGGGCCGACCGGTCTGCCGATGGGTA

RCC1218 TTGCAGTTAAAACGCTCGTAGTCGGATTTCGGGGCGGGCCGACCGGTCTGCCGATGGGTA

RCC1219 TTGCAGTTAAAACGCTCGTAGTCGGATTTCGGGGCGGGCCGACCGGTCTGCCGATGGGTA

RCC1221 TTGCAGTTAAAACGCTCGTAGTCGGATTTCGGGGCGGGCCGACCGGTCTGCCGATGGGTA

RCC1225 TTGCAGTTAAAACGCTCGTAGTCGGATTTCGGGGCGGGCCGACCGGTCTGCCGATGGGTA

RCC1227 TTGCAGTTAAAACGCTCGTAGTCGGATTTCGGGGCGGGCCGACCGGTCTGCCGATGGGTA

RCC1228 TTGCAGTTAAAACGCTCGTAGTCGGATTTCGGGGCGGGCCGACCGGTCTGCCGATGGGTA

RCC1229 TTGCAGTTAAAACGCTCGTAGTCGGATTTCGGGGCGGGCCGACCGGTCTGCCGATGGGTA

RCC1245 TTGCAGTTAAAACGCTCGTAGTCGGATTTCGGGGCGGGCCGACCGGTCTGCCGATGGGTA

RCC1246 TTGCAGTTAAAACGCTCGTAGTCGGATTTCGGGGCGGGCCGACCGGTCTGCCGATGGGTA

RCC1247 TTGCAGTTAAAACGCTCGTAGTCGGATTTCGGGGCGGGCCGACCGGTCTGCCGATGGGTA

RCC1249 TTGCAGTTAAAACGCTCGTAGTCGGATTTCGGGGCGGGCCGACCGGTCTGCCGATGGGTA

RCC1250 TTGCAGTTAAAACGCTCGTAGTCGGATTTCGGGGCGGGCCGACCGGTCTGCCGATGGGTA

RCC1251 TTGCAGTTAAAACGCTCGTAGTCGGATTTCGGGGCGGGCCGACCGGTCTGCCGATGGGTA

RCC1254 TTGCAGTTAAAACGCTCGTAGTCGGATTTCGGGGCGGGCCGACCGGTCTGCCGATGGGTA

RCC1257 TTGCAGTTAAAACGCTCGTAGTCGGATTTCGGGGCGGGCCGACCGGTCTGCCGATGGGTA

RCC1258 TTGCAGTTAAAACGCTCGTAGTCGGATTTCGGGGCGGGCCGACCGGTCTGCCGATGGGTA

RCC1322 TTGCAGTTAAAACGCTCGTAGTCGGATTTCGGGGCGGGCCGACCGGTCTGCCGATGGGTA

ESP7414 TTGCAGTTAAAACGCTCGTAGTCGGATTTCGGGGCGGGCCGACCGGTCTGCCGATGGGTA

************************************************************

CCMP374 TGCACTGGCCGGCGCGTCCTTCCACCCGGAGACCGCGCCTACTCTTAACTGAGCGGGCGC

920PML TGCACTGGCCGGCGCGTCCTTCCACCCGGAGACCGCGCCTACTCTTAACTGAGCGGGCGC

Ch25_90 TGCACTGGCCGGCGCGTCCTTCCACCCGGAGACCGCGCCTACTCTTAACTGAGCGGGCGC

CCAP920_9 TGCACTGGCCGGCGCGTCCTTCCACCCGGAGACCGCGCCTACTCTTAACTGAGCGGGCGC

UNC1419 TGCACTGGCCGGCGCGTCCTTCCACCCGGAGACCGCGCCTACTCTTAACTGAGCGGGCGC

RCC6856 TGCACTGGCCGGCGCGTCCTTCCACCCGGAGACCGCGCCTACTCTTAACTGAGCGGGCGC

RCC909 TGCACTGGCCGGCGCGTCCTTCCACCCGGAGACCGCGCCTACTCTTAACTGAGCGGGCGC

KMMCC_H-18 TGCACTGGCCGGCGCGTCCTTCCACCCGGAGACCGCGCCTACTCTTAACTGAGCGGGCGC

RCC1208 TGCACTGGCCGGCGCGTCCTTCCACCCGGAGACCGCGCCTACTCTTAACTGAGCGGGCGC

RCC1210 TGCACTGGCCGGCGCGTCCTTCCACCCGGAGACCGCGCCTACTCTTAACTGAGCGGGCGC

RCC1213 TGCACTGGCCGGCGCGTCCTTCCACCCGGAGACCGCGCCTACTCTTAACTGAGCGGGCGC

RCC1214 TGCACTGGCCGGCGCGTCCTTCCACCCGGAGACCGCGCCTACTCTTAACTGAGCGGGCGC

RCC1218 TGCACTGGCCGGCGCGTCCTTCCACCCGGAGACCGCGCCTACTCTTAACTGAGCGGGCGC

RCC1219 TGCACTGGCCGGCGCGTCCTTCCACCCGGAGACCGCGCCTACTCTTAACTGAGCGGGCGC

RCC1221 TGCACTGGCCGGCGCGTCCTTCCACCCGGAGACCGCGCCTACTCTTAACTGAGCGGGCGC

RCC1225 TGCACTGGCCGGCGCGTCCTTCCACCCGGAGACCGCGCCTACTCTTAACTGAGCGGGCGC

RCC1227 TGCACTGGCCGGCGCGTCCTTCCACCCGGAGACCGCGCCTACTCTTAACTGAGCGGGCGC

RCC1228 TGCACTGGCCGGCGCGTCCTTCCACCCGGAGACCGCGCCTACTCTTAACTGAGCGGGCGC

RCC1229 TGCACTGGCCGGCGCGTCCTTCCACCCGGAGACCGCGCCTACTCTTAACTGAGCGGGCGC

RCC1245 TGCACTGGCCGGCGCGTCCTTCCACCCGGAGACCGCGCCTACTCTTAACTGAGCGGGCGC

RCC1246 TGCACTGGCCGGCGCGTCCTTCCACCCGGAGACCGCGCCTACTCTTAACTGAGCGGGCGC

RCC1247 TGCACTGGCCGGCGCGTCCTTCCACCCGGAGACCGCGCCTACTCTTAACTGAGCGGGCGC

RCC1249 TGCACTGGCCGGCGCGTCCTTCCACCCGGAGACCGCGCCTACTCTTAACTGAGCGGGCGC

RCC1250 TGCACTGGCCGGCGCGTCCTTCCACCCGGAGACCGCGCCTACTCTTAACTGAGCGGGCGC

RCC1251 TGCACTGGCCGGCGCGTCCTTCCACCCGGAGACCGCGCCTACTCTTAACTGAGCGGGCGC

RCC1254 TGCACTGGCCGGCGCGTCCTTCCACCCGGAGACCGCGCCTACTCTTAACTGAGCGGGCGC

RCC1257 TGCACTGGCCGGCGCGTCCTTCCACCCGGAGACCGCGCCTACTCTTAACTGAGCGGGCGC

RCC1258 TGCACTGGCCGGCGCGTCCTTCCACCCGGAGACCGCGCCTACTCTTAACTGAGCGGGCGC

RCC1322 TGCACTGGCCGGCGCGTCCTTCCACCCGGAGACCGCGCCTACTCTTAACTGAGCGGGCGC

ESP7414 TGCACTGGCCGGCGCGTCCTTCCACCCGGAGACCGCGCCTACTCTTAACTGAGCGGGCGC

************************************************************

CCMP374 GGGAGACGGGTCTTTTACTTTGAAAAAATCAGAGTGTTTCAAGCAGGCAGTCGCTCTTGC

920PML GGGAGACGGGTCTTTTACTTTGAAAAAATCAGAGTGTTTCAAGCAGGCAGTCGCTCTTGC

Ch25_90 GGGAGACGGGTCTTTTACTTTGAAAAAATCAGAGTGTTTCAAGCAGGCAGTCGCTCTTGC

CCAP920_9 GGGAGACGGGTCTTTTACTTTGAAAAAATCAGAGTGTTTCAAGCAGGCAGTCGCTCTTGC

UNC1419 GGGAGACGGGTCTTTTACTTTGAAAAAATCAGAGTGTTTCAAGCAGGCAGTCGCTCTTGC

RCC6856 GGGAGACGGGTCTTTTACTTTGAAAAAATCAGAGTGTTTCAAGCAGGCAGTCGCTCTTGC

RCC909 GGGAGACGGGTCTTTTACTTTGAAAAAATCAGAGTGTTTCAAGCAGGCAGTCGCTCTTGC

KMMCC_H-18 GGGAGACGGGTCTTTTACTTTGAAAAAATCAGAGTGTTTCAAGCAGGCAGTCGCTCTTGC

RCC1208 GGGAGACGGGTCTTTTACTTTGAAAAAATCAGAGTGTTTCAAGCAGGCAGTCGCTCTTGC

RCC1210 GGGAGACGGGTCTTTTACTTTGAAAAAATCAGAGTGTTTCAAGCAGGCAGTCGCTCTTGC

RCC1213 GGGAGACGGGTCTTTTACTTTGAAAAAATCAGAGTGTTTCAAGCAGGCAGTCGCTCTTGC

RCC1214 GGGAGACGGGTCTTTTACTTTGAAAAAATCAGAGTGTTTCAAGCAGGCAGTCGCTCTTGC

RCC1218 GGGAGACGGGTCTTTTACTTTGAAAAAATCAGAGTGTTTCAAGCAGGCAGTCGCTCTTGC

RCC1219 GGGAGACGGGTCTTTTACTTTGAAAAAATCAGAGTGTTTCAAGCAGGCAGTCGCTCTTGC

RCC1221 GGGAGACGGGTCTTTTACTTTGAAAAAATCAGAGTGTTTCAAGCAGGCAGTCGCTCTTGC

RCC1225 GGGAGACGGGTCTTTTACTTTGAAAAAATCAGAGTGTTTCAAGCAGGCAGTCGCTCTTGC

RCC1227 GGGAGACGGGTCTTTTACTTTGAAAAAATCAGAGTGTTTCAAGCAGGCAGTCGCTCTTGC

RCC1228 GGGAGACGGGTCTTTTACTTTGAAAAAATCAGAGTGTTTCAAGCAGGCAGTCGCTCTTGC

RCC1229 GGGAGACGGGTCTTTTACTTTGAAAAAATCAGAGTGTTTCAAGCAGGCAGTCGCTCTTGC

RCC1245 GGGAGACGGGTCTTTTACTTTGAAAAAATCAGAGTGTTTCAAGCAGGCAGTCGCTCTTGC

RCC1246 GGGAGACGGGTCTTTTACTTTGAAAAAATCAGAGTGTTTCAAGCAGGCAGTCGCTCTTGC

RCC1247 GGGAGACGGGTCTTTTACTTTGAAAAAATCAGAGTGTTTCAAGCAGGCAGTCGCTCTTGC

RCC1249 GGGAGACGGGTCTTTTACTTTGAAAAAATCAGAGTGTTTCAAGCAGGCAGTCGCTCTTGC

RCC1250 GGGAGACGGGTCTTTTACTTTGAAAAAATCAGAGTGTTTCAAGCAGGCAGTCGCTCTTGC

RCC1251 GGGAGACGGGTCTTTTACTTTGAAAAAATCAGAGTGTTTCAAGCAGGCAGTCGCTCTTGC

RCC1254 GGGAGACGGGTCTTTTACTTTGAAAAAATCAGAGTGTTTCAAGCAGGCAGTCGCTCTTGC

RCC1257 GGGAGACGGGTCTTTTACTTTGAAAAAATCAGAGTGTTTCAAGCAGGCAGTCGCTCTTGC

RCC1258 GGGAGACGGGTCTTTTACTTTGAAAAAATCAGAGTGTTTCAAGCAGGCAGTCGCTCTTGC

RCC1322 GGGAGACGGGTCTTTTACTTTGAAAAAATCAGAGTGTTTCAAGCAGGCAGTCGCTCTTGC

ESP7414 GGGAGACGGGTCTTTTACTTTGAAAAAATCAGAGTGTTTCAAGCAGGCAGTCGCTCTTGC

************************************************************

CCMP374 ATGGATTAGCATGGGATAATGAAATAGGACTCTGGTGCTATTTTGTTGGTTTCGAACACC

920PML ATGGATTAGCATGGGATAATGAAATAGGACTCTGGTGCTATTTTGTTGGTTTCGAACACC

Ch25_90 ATGGATTAGCATGGGATAATGAAATAGGACTCTGGTGCTATTTTGTTGGTTTCGAACACC

CCAP920_9 ATGGATTAGCATGGGATAATGAAATAGGACTCTGGTGCTATTTTGTTGGTTTCGAACACC

UNC1419 ATGGATTAGCATGGGATAATGAAATAGGACTCTGGTGCTATTTTGTTGGTTTCGAACACC

RCC6856 ATGGATTAGCATGGGATAATGAAATAGGACTCTGGTGCTATTTTGTTGGTTTCGAACACC

RCC909 ATGGATTAGCATGGGATAATGAAATAGGACTCTGGTGCTATTTTGTTGGTTTCGAACACC

KMMCC_H-18 ATGGATTAGCATGGGATAATGAAATAGGACTCTGGTGCTATTTTGTTGGTTTCGAACACC

RCC1208 ATGGATTAGCATGGGATAATGAAATAGGACTCTGGTGCTATTTTGTTGGTTTCGAACACC

RCC1210 ATGGATTAGCATGGGATAATGAAATAGGACTCTGGTGCTATTTTGTTGGTTTCGAACACC

RCC1213 ATGGATTAGCATGGGATAATGAAATAGGACTCTGGTGCTATTTTGTTGGTTTCGAACACC

RCC1214 ATGGATTAGCATGGGATAATGAAATAGGACTCTGGTGCTATTTTGTTGGTTTCGAACACC

RCC1218 ATGGATTAGCATGGGATAATGAAATAGGACTCTGGTGCTATTTTGTTGGTTTCGAACACC

RCC1219 ATGGATTAGCATGGGATAATGAAATAGGACTCTGGTGCTATTTTGTTGGTTTCGAACACC

RCC1221 ATGGATTAGCATGGGATAATGAAATAGGACTCTGGTGCTATTTTGTTGGTTTCGAACACC

RCC1225 ATGGATTAGCATGGGATAATGAAATAGGACTCTGGTGCTATTTTGTTGGTTTCGAACACC

RCC1227 ATGGATTAGCATGGGATAATGAAATAGGACTCTGGTGCTATTTTGTTGGTTTCGAACACC

RCC1228 ATGGATTAGCATGGGATAATGAAATAGGACTCTGGTGCTATTTTGTTGGTTTCGAACACC

RCC1229 ATGGATTAGCATGGGATAATGAAATAGGACTCTGGTGCTATTTTGTTGGTTTCGAACACC

RCC1245 ATGGATTAGCATGGGATAATGAAATAGGACTCTGGTGCTATTTTGTTGGTTTCGAACACC

RCC1246 ATGGATTAGCATGGGATAATGAAATAGGACTCTGGTGCTATTTTGTTGGTTTCGAACACC

RCC1247 ATGGATTAGCATGGGATAATGAAATAGGACTCTGGTGCTATTTTGTTGGTTTCGAACACC

RCC1249 ATGGATTAGCATGGGATAATGAAATAGGACTCTGGTGCTATTTTGTTGGTTTCGAACACC

RCC1250 ATGGATTAGCATGGGATAATGAAATAGGACTCTGGTGCTATTTTGTTGGTTTCGAACACC

RCC1251 ATGGATTAGCATGGGATAATGAAATAGGACTCTGGTGCTATTTTGTTGGTTTCGAACACC

RCC1254 ATGGATTAGCATGGGATAATGAAATAGGACTCTGGTGCTATTTTGTTGGTTTCGAACACC

RCC1257 ATGGATTAGCATGGGATAATGAAATAGGACTCTGGTGCTATTTTGTTGGTTTCGAACACC

RCC1258 ATGGATTAGCATGGGATAATGAAATAGGACTCTGGTGCTATTTTGTTGGTTTCGAACACC

RCC1322 ATGGATTAGCATGGGATAATGAAATAGGACTCTGGTGCTATTTTGTTGGTTTCGAACACC

ESP7414 ATGGATTAGCATGGGATAATGAAATAGGACTCTGGTGCTATTTTGTTGGTTTCGAACACC

************************************************************

CCMP374 GGAGTAATGATTAACAGGGACAGTCAGGGGCACTCGTATTCCGCCGAGAGAGGTGAAATT

920PML GGAGTAATGATTAACAGGGACAGTCAGGGGCACTCGTATTCCGCCGAGAGAGGTGAAATT

Ch25_90 GGAGTAATGATTAACAGGGACAGTCAGGGGCACTCGTATTCCGCCGAGAGAGGTGAAATT

CCAP920_9 GGAGTAATGATTAACAGGGACAGTCAGGGGCACTCGTATTCCGCCGAGAGAGGTGAAATT

UNC1419 GGAGTAATGATTAACAGGGACAGTCAGGGGCACTCGTATTCCGCCGAGAGAGGTGAAATT

RCC6856 GGAGTAATGATTAACAGGGACAGTCAGGGGCACTCGTATTCCGCCGAGAGAGGTGAAATT

RCC909 GGAGTAATGATTAACAGGGACAGTCAGGGGCACTCGTATTCCGCCGAGAGAGGTGAAATT

KMMCC_H-18 GGAGTAATGATTAACAGGGACAGTCAGGGGCACTCGTATTCCGCCGAGAGAGGTGAAATT

RCC1208 GGAGTAATGATTAACAGGGACAGTCAGGGGCACTCGTATTCCGCCGAGAGAGGTGAAATT

RCC1210 GGAGTAATGATTAACAGGGACAGTCAGGGGCACTCGTATTCCGCCGAGAGAGGTGAAATT

RCC1213 GGAGTAATGATTAACAGGGACAGTCAGGGGCACTCGTATTCCGCCGAGAGAGGTGAAATT

RCC1214 GGAGTAATGATTAACAGGGACAGTCAGGGGCACTCGTATTCCGCCGAGAGAGGTGAAATT

RCC1218 GGAGTAATGATTAACAGGGACAGTCAGGGGCACTCGTATTCCGCCGAGAGAGGTGAAATT

RCC1219 GGAGTAATGATTAACAGGGACAGTCAGGGGCACTCGTATTCCGCCGAGAGAGGTGAAATT

RCC1221 GGAGTAATGATTAACAGGGACAGTCAGGGGCACTCGTATTCCGCCGAGAGAGGTGAAATT

RCC1225 GGAGTAATGATTAACAGGGACAGTCAGGGGCACTCGTATTCCGCCGAGAGAGGTGAAATT

RCC1227 GGAGTAATGATTAACAGGGACAGTCAGGGGCACTCGTATTCCGCCGAGAGAGGTGAAATT

RCC1228 GGAGTAATGATTAACAGGGACAGTCAGGGGCACTCGTATTCCGCCGAGAGAGGTGAAATT

RCC1229 GGAGTAATGATTAACAGGGACAGTCAGGGGCACTCGTATTCCGCCGAGAGAGGTGAAATT

RCC1245 GGAGTAATGATTAACAGGGACAGTCAGGGGCACTCGTATTCCGCCGAGAGAGGTGAAATT

RCC1246 GGAGTAATGATTAACAGGGACAGTCAGGGGCACTCGTATTCCGCCGAGAGAGGTGAAATT

RCC1247 GGAGTAATGATTAACAGGGACAGTCAGGGGCACTCGTATTCCGCCGAGAGAGGTGAAATT

RCC1249 GGAGTAATGATTAACAGGGACAGTCAGGGGCACTCGTATTCCGCCGAGAGAGGTGAAATT

RCC1250 GGAGTAATGATTAACAGGGACAGTCAGGGGCACTCGTATTCCGCCGAGAGAGGTGAAATT

RCC1251 GGAGTAATGATTAACAGGGACAGTCAGGGGCACTCGTATTCCGCCGAGAGAGGTGAAATT

RCC1254 GGAGTAATGATTAACAGGGACAGTCAGGGGCACTCGTATTCCGCCGAGAGAGGTGAAATT

RCC1257 GGAGTAATGATTAACAGGGACAGTCAGGGGCACTCGTATTCCGCCGAGAGAGGTGAAATT

RCC1258 GGAGTAATGATTAACAGGGACAGTCAGGGGCACTCGTATTCCGCCGAGAGAGGTGAAATT

RCC1322 GGAGTAATGATTAACAGGGACAGTCAGGGGCACTCGTATTCCGCCGAGAGAGGTGAAATT

ESP7414 GGAGTAATGATTAACAGGGACAGTCAGGGGCACTCGTATTCCGCCGAGAGAGGTGAAATT

************************************************************

CCMP374 CTCAGACCAGCGGAAGACGAACCACTGCGAAAGCATTTGCCAGGGATGTTTTCACTGATC

920PML CTCAGACCAGCGGAAGACGAACCACTGCGAAAGCATTTGCCAGGGATGTTTTCACTGATC

Ch25_90 CTCAGACCAGCGGAAGACGAACCACTGCGAAAGCATTTGCCAGGGATGTTTTCACTGATC

CCAP920_9 CTCAGACCAGCGGAAGACGAACCACTGCGAAAGCATTTGCCAGGGATGTTTTCACTGATC

UNC1419 CTCAGACCAGCGGAAGACGAACCACTGCGAAAGCATTTGCCAGGGATGTTTTCACTGATC

RCC6856 CTCAGACCAGCGGAAGACGAACCACTGCGAAAGCATTTGCCAGGGATGTTTTCACTGATC

RCC909 CTCAGACCAGCGGAAGACGAACCACTGCGAAAGCATTTGCCAGGGATGTTTTCACTGATC

KMMCC_H-18 CTCAGACCAGCGGAAGACGAACCACTGCGAAAGCATTTGCCAGGGATGTTTTCACTGATC

RCC1208 CTCAGACCAGCGGAAGACGAACCACTGCGAAAGCATTTGCCAGGGATGTTTTCACTGATC

RCC1210 CTCAGACCAGCGGAAGACGAACCACTGCGAAAGCATTTGCCAGGGATGTTTTCACTGATC

RCC1213 CTCAGACCAGCGGAAGACGAACCACTGCGAAAGCATTTGCCAGGGATGTTTTCACTGATC

RCC1214 CTCAGACCAGCGGAAGACGAACCACTGCGAAAGCATTTGCCAGGGATGTTTTCACTGATC

RCC1218 CTCAGACCAGCGGAAGACGAACCACTGCGAAAGCATTTGCCAGGGATGTTTTCACTGATC

RCC1219 CTCAGACCAGCGGAAGACGAACCACTGCGAAAGCATTTGCCAGGGATGTTTTCACTGATC

RCC1221 CTCAGACCAGCGGAAGACGAACCACTGCGAAAGCATTTGCCAGGGATGTTTTCACTGATC

RCC1225 CTCAGACCAGCGGAAGACGAACCACTGCGAAAGCATTTGCCAGGGATGTTTTCACTGATC

RCC1227 CTCAGACCAGCGGAAGACGAACCACTGCGAAAGCATTTGCCAGGGATGTTTTCACTGATC

RCC1228 CTCAGACCAGCGGAAGACGAACCACTGCGAAAGCATTTGCCAGGGATGTTTTCACTGATC

RCC1229 CTCAGACCAGCGGAAGACGAACCACTGCGAAAGCATTTGCCAGGGATGTTTTCACTGATC

RCC1245 CTCAGACCAGCGGAAGACGAACCACTGCGAAAGCATTTGCCAGGGATGTTTTCACTGATC

RCC1246 CTCAGACCAGCGGAAGACGAACCACTGCGAAAGCATTTGCCAGGGATGTTTTCACTGATC

RCC1247 CTCAGACCAGCGGAAGACGAACCACTGCGAAAGCATTTGCCAGGGATGTTTTCACTGATC

RCC1249 CTCAGACCAGCGGAAGACGAACCACTGCGAAAGCATTTGCCAGGGATGTTTTCACTGATC

RCC1250 CTCAGACCAGCGGAAGACGAACCACTGCGAAAGCATTTGCCAGGGATGTTTTCACTGATC

RCC1251 CTCAGACCAGCGGAAGACGAACCACTGCGAAAGCATTTGCCAGGGATGTTTTCACTGATC

RCC1254 CTCAGACCAGCGGAAGACGAACCACTGCGAAAGCATTTGCCAGGGATGTTTTCACTGATC

RCC1257 CTCAGACCAGCGGAAGACGAACCACTGCGAAAGCATTTGCCAGGGATGTTTTCACTGATC

RCC1258 CTCAGACCAGCGGAAGACGAACCACTGCGAAAGCATTTGCCAGGGATGTTTTCACTGATC

RCC1322 CTCAGACCAGCGGAAGACGAACCACTGCGAAAGCATTTGCCAGGGATGTTTTCACTGATC

ESP7414 CTCAGACCAGCGGAAGACGAACCACTGCGAAAGCATTTGCCAGGGATGTTTTCACTGATC

************************************************************

CCMP374 AAGAACGAAAGTTAGGGGATCGAAGACGATCAGATACCGTCGTAGTCTTAACCATAAACC

920PML AAGAACGAAAGTTAGGGGATCGAAGACGATCAGATACCGTCGTAGTCTTAACCATAAACC

Ch25_90 AAGAACGAAAGTTAGGGGATCGAAGACGATCAGATACCGTCGTAGTCTTAACCATAAACC

CCAP920_9 AAGAACGAAAGTTAGGGGATCGAAGACGATCAGATACCGTCGTAGTCTTAACCATAAACC

UNC1419 AAGAACGAAAGTTAGGGGATCGAAGACGATCAGATACCGTCGTAGTCTTAACCATAAACC

RCC6856 AAGAACGAAAGTTAGGGGATCGAAGACGATCAGATACCGTCGTAGTCTTAACCATAAACC

RCC909 AAGAACGAAAGTTAGGGGATCGAAGACGATCAGATACCGTCGTAGTCTTAACCATAAACC

KMMCC_H-18 AAGAACGAAAGTTAGGGGATCGAAGACGATCAGATACCGTCGTAGTCTTAACCATAAACC

RCC1208 AAGAACGAAAGTTAGGGGATCGAAGACGATCAGATACCGTCGTAGTCTTAACCATAAACC

RCC1210 AAGAACGAAAGTTAGGGGATCGAAGACGATCAGATACCGTCGTAGTCTTAACCATAAACC

RCC1213 AAGAACGAAAGTTAGGGGATCGAAGACGATCAGATACCGTCGTAGTCTTAACCATAAACC

RCC1214 AAGAACGAAAGTTAGGGGATCGAAGACGATCAGATACCGTCGTAGTCTTAACCATAAACC

RCC1218 AAGAACGAAAGTTAGGGGATCGAAGACGATCAGATACCGTCGTAGTCTTAACCATAAACC

RCC1219 AAGAACGAAAGTTAGGGGATCGAAGACGATCAGATACCGTCGTAGTCTTAACCATAAACC

RCC1221 AAGAACGAAAGTTAGGGGATCGAAGACGATCAGATACCGTCGTAGTCTTAACCATAAACC

RCC1225 AAGAACGAAAGTTAGGGGATCGAAGACGATCAGATACCGTCGTAGTCTTAACCATAAACC

RCC1227 AAGAACGAAAGTTAGGGGATCGAAGACGATCAGATACCGTCGTAGTCTTAACCATAAACC

RCC1228 AAGAACGAAAGTTAGGGGATCGAAGACGATCAGATACCGTCGTAGTCTTAACCATAAACC

RCC1229 AAGAACGAAAGTTAGGGGATCGAAGACGATCAGATACCGTCGTAGTCTTAACCATAAACC

RCC1245 AAGAACGAAAGTTAGGGGATCGAAGACGATCAGATACCGTCGTAGTCTTAACCATAAACC

RCC1246 AAGAACGAAAGTTAGGGGATCGAAGACGATCAGATACCGTCGTAGTCTTAACCATAAACC

RCC1247 AAGAACGAAAGTTAGGGGATCGAAGACGATCAGATACCGTCGTAGTCTTAACCATAAACC

RCC1249 AAGAACGAAAGTTAGGGGATCGAAGACGATCAGATACCGTCGTAGTCTTAACCATAAACC

RCC1250 AAGAACGAAAGTTAGGGGATCGAAGACGATCAGATACCGTCGTAGTCTTAACCATAAACC

RCC1251 AAGAACGAAAGTTAGGGGATCGAAGACGATCAGATACCGTCGTAGTCTTAACCATAAACC

RCC1254 AAGAACGAAAGTTAGGGGATCGAAGACGATCAGATACCGTCGTAGTCTTAACCATAAACC

RCC1257 AAGAACGAAAGTTAGGGGATCGAAGACGATCAGATACCGTCGTAGTCTTAACCATAAACC

RCC1258 AAGAACGAAAGTTAGGGGATCGAAGACGATCAGATACCGTCGTAGTCTTAACCATAAACC

RCC1322 AAGAACGAAAGTTAGGGGATCGAAGACGATCAGATACCGTCGTAGTCTTAACCATAAACC

ESP7414 AAGAACGAAAGTTAGGGGATCGAAGACGATCAGATACCGTCGTAGTCTTAACCATAAACC

************************************************************

CCMP374 ATGCCGACTAGGGATTGGAGGATGTTCCATTTGTGACTCCTTCAGCACCTTTCGGGAAAC

920PML ATGCCGACTAGGGATTGGAGGATGTTCCATTTGTGACTCCTTCAGCACCTTTCGGGAAAC

Ch25_90 ATGCCGACTAGGGATTGGAGGATGTTCCATTTGTGACTCCTTCAGCACCTTTCGGGAAAC

CCAP920_9 ATGCCGACTAGGGATTGGAGGATGTTCCATTTGTGACTCCTTCAGCACCTTTCGGGAAAC

UNC1419 ATGCCGACTAGGGATTGGAGGATGTTCCATTTGTGACTCCTTCAGCACCTTTCGGGAAAC

RCC6856 ATGCCGACTAGGGATTGGAGGATGTTCCATTTGTGACTCCTTCAGCACCTTTCGGGAAAC

RCC909 ATGCCGACTAGGGATTGGAGGATGTTCCATTTGTGACTCCTTCAGCACCTTTCGGGAAAC

KMMCC_H-18 ATGCCGACTAGGGATTGGAGGATGTTCCATTTGTGACTCCTTCAGCACCTTTCGGGAAAC

RCC1208 ATGCCGACTAGGGATTGGAGGATGTTCCATTTGTGACTCCTTCAGCACCTTTCGGGAAAC

RCC1210 ATGCCGACTAGGGATTGGAGGATGTTCCATTTGTGACTCCTTCAGCACCTTTCGGGAAAC

RCC1213 ATGCCGACTAGGGATTGGAGGATGTTCCATTTGTGACTCCTTCAGCACCTTTCGGGAAAC

RCC1214 ATGCCGACTAGGGATTGGAGGATGTTCCATTTGTGACTCCTTCAGCACCTTTCGGGAAAC

RCC1218 ATGCCGACTAGGGATTGGAGGATGTTCCATTTGTGACTCCTTCAGCACCTTTCGGGAAAC

RCC1219 ATGCCGACTAGGGATTGGAGGATGTTCCATTTGTGACTCCTTCAGCACCTTTCGGGAAAC

RCC1221 ATGCCGACTAGGGATTGGAGGATGTTCCATTTGTGACTCCTTCAGCACCTTTCGGGAAAC

RCC1225 ATGCCGACTAGGGATTGGAGGATGTTCCATTTGTGACTCCTTCAGCACCTTTCGGGAAAC

RCC1227 ATGCCGACTAGGGATTGGAGGATGTTCCATTTGTGACTCCTTCAGCACCTTTCGGGAAAC

RCC1228 ATGCCGACTAGGGATTGGAGGATGTTCCATTTGTGACTCCTTCAGCACCTTTCGGGAAAC

RCC1229 ATGCCGACTAGGGATTGGAGGATGTTCCATTTGTGACTCCTTCAGCACCTTTCGGGAAAC

RCC1245 ATGCCGACTAGGGATTGGAGGATGTTCCATTTGTGACTCCTTCAGCACCTTTCGGGAAAC

RCC1246 ATGCCGACTAGGGATTGGAGGATGTTCCATTTGTGACTCCTTCAGCACCTTTCGGGAAAC

RCC1247 ATGCCGACTAGGGATTGGAGGATGTTCCATTTGTGACTCCTTCAGCACCTTTCGGGAAAC

RCC1249 ATGCCGACTAGGGATTGGAGGATGTTCCATTTGTGACTCCTTCAGCACCTTTCGGGAAAC

RCC1250 ATGCCGACTAGGGATTGGAGGATGTTCCATTTGTGACTCCTTCAGCACCTTTCGGGAAAC

RCC1251 ATGCCGACTAGGGATTGGAGGATGTTCCATTTGTGACTCCTTCAGCACCTTTCGGGAAAC

RCC1254 ATGCCGACTAGGGATTGGAGGATGTTCCATTTGTGACTCCTTCAGCACCTTTCGGGAAAC

RCC1257 ATGCCGACTAGGGATTGGAGGATGTTCCATTTGTGACTCCTTCAGCACCTTTCGGGAAAC

RCC1258 ATGCCGACTAGGGATTGGAGGATGTTCCATTTGTGACTCCTTCAGCACCTTTCGGGAAAC

RCC1322 ATGCCGACTAGGGATTGGAGGATGTTCCATTTGTGACTCCTTCAGCACCTTTCGGGAAAC

ESP7414 ATGCCGACTAGGGATTGGAGGATGTTCCATTTGTGACTCCTTCAGCACCTTTCGGGAAAC

************************************************************

CCMP374 TAAAGTCTTTGGGTTCCGGGGGGAGTATGGTCGCAAGGCTGAAACTTAAAGGAATTGACG

920PML TAAAGTCTTTGGGTTCCGGGGGGAGTATGGTCGCAAGGCTGAAACTTAAAGGAATTGACG

Ch25_90 TAAAGTCTTTGGGTTCCGGGGGGAGTATGGTCGCAAGGCTGAAACTTAAAGGAATTGACG

CCAP920_9 TAAAGTCTTTGGGTTCCGGGGGGAGTATGGTCGCAAGGCTGAAACTTAAAGGAATTGACG

UNC1419 TAAAGTCTTTGGGTTCCGGGGGGAGTATGGTCGCAAGGCTGAAACTTAAAGGAATTGACG

RCC6856 TAAAGTCTTTGGGTTCCGGGGGGAGTATGGTCGCAAGGCTGAAACTTAAAGGAATTGACG

RCC909 TAAAGTCTTTGGGTTCCGGGGGGAGTATGGTCGCAAGGCTGAAACTTAAAGGAATTGACG

KMMCC_H-18 TAAAGTCTTTGGGTTCCGGGGGGAGTATGGTCGCAAGGCTGAAACTTAAAGGAATTGACG

RCC1208 TAAAGTCTTTGGGTTCCGGGGGGAGTATGGTCGCAAGGCTGAAACTTAAAGGAATTGACG

RCC1210 TAAAGTCTTTGGGTTCCGGGGGGAGTATGGTCGCAAGGCTGAAACTTAAAGGAATTGACG

RCC1213 TAAAGTCTTTGGGTTCCGGGGGGAGTATGGTCGCAAGGCTGAAACTTAAAGGAATTGACG

RCC1214 TAAAGTCTTTGGGTTCCGGGGGGAGTATGGTCGCAAGGCTGAAACTTAAAGGAATTGACG

RCC1218 TAAAGTCTTTGGGTTCCGGGGGGAGTATGGTCGCAAGGCTGAAACTTAAAGGAATTGACG

RCC1219 TAAAGTCTTTGGGTTCCGGGGGGAGTATGGTCGCAAGGCTGAAACTTAAAGGAATTGACG

RCC1221 TAAAGTCTTTGGGTTCCGGGGGGAGTATGGTCGCAAGGCTGAAACTTAAAGGAATTGACG

RCC1225 TAAAGTCTTTGGGTTCCGGGGGGAGTATGGTCGCAAGGCTGAAACTTAAAGGAATTGACG

RCC1227 TAAAGTCTTTGGGTTCCGGGGGGAGTATGGTCGCAAGGCTGAAACTTAAAGGAATTGACG

RCC1228 TAAAGTCTTTGGGTTCCGGGGGGAGTATGGTCGCAAGGCTGAAACTTAAAGGAATTGACG

RCC1229 TAAAGTCTTTGGGTTCCGGGGGGAGTATGGTCGCAAGGCTGAAACTTAAAGGAATTGACG

RCC1245 TAAAGTCTTTGGGTTCCGGGGGGAGTATGGTCGCAAGGCTGAAACTTAAAGGAATTGACG

RCC1246 TAAAGTCTTTGGGTTCCGGGGGGAGTATGGTCGCAAGGCTGAAACTTAAAGGAATTGACG

RCC1247 TAAAGTCTTTGGGTTCCGGGGGGAGTATGGTCGCAAGGCTGAAACTTAAAGGAATTGACG

RCC1249 TAAAGTCTTTGGGTTCCGGGGGGAGTATGGTCGCAAGGCTGAAACTTAAAGGAATTGACG

RCC1250 TAAAGTCTTTGGGTTCCGGGGGGAGTATGGTCGCAAGGCTGAAACTTAAAGGAATTGACG

RCC1251 TAAAGTCTTTGGGTTCCGGGGGGAGTATGGTCGCAAGGCTGAAACTTAAAGGAATTGACG

RCC1254 TAAAGTCTTTGGGTTCCGGGGGGAGTATGGTCGCAAGGCTGAAACTTAAAGGAATTGACG

RCC1257 TAAAGTCTTTGGGTTCCGGGGGGAGTATGGTCGCAAGGCTGAAACTTAAAGGAATTGACG

RCC1258 TAAAGTCTTTGGGTTCCGGGGGGAGTATGGTCGCAAGGCTGAAACTTAAAGGAATTGACG

RCC1322 TAAAGTCTTTGGGTTCCGGGGGGAGTATGGTCGCAAGGCTGAAACTTAAAGGAATTGACG

ESP7414 TAAAGTCTTTGGGTTCCGGGGGGAGTATGGTCGCAAGGCTGAAACTTAAAGGAATTGACG

************************************************************

CCMP374 GAAGGGCACCACCAGGAGTGGAGCCTGCGGCTTAATTTGACTCAACACGGGGAAACTTAC

920PML GAAGGGCACCACCAGGAGTGGAGCCTGCGGCTTAATTTGACTCAACACGGGGAAACTTAC

Ch25_90 GAAGGGCACCACCAGGAGTGGAGCCTGCGGCTTAATTTGACTCAACACGGGGAAACTTAC

CCAP920_9 GAAGGGCACCACCAGGAGTGGAGCCTGCGGCTTAATTTGACTCAACACGGGGAAACTTAC

UNC1419 GAAGGGCACCACCAGGAGTGGAGCCTGCGGCTTAATTTGACTCAACACGGGGAAACTTAC

RCC6856 GAAGGGCACCACCAGGAGTGGAGCCTGCGGCTTAATTTGACTCAACACGGGGAAACTTAC

RCC909 GAAGGGCACCACCAGGAGTGGAGCCTGCGGCTTAATTTGACTCAACACGGGGAAACTTAC

KMMCC_H-18 GAAGGGCACCACCAGGAGTGGAGCCTGCGGCTTAATTTGACTCAACACGGGGAAACTTAC

RCC1208 GAAGGGCACCACCAGGAGTGGAGCCTGCGGCTTAATTTGACTCAACACGGGGAAACTTAC

RCC1210 GAAGGGCACCACCAGGAGTGGAGCCTGCGGCTTAATTTGACTCAACACGGGGAAACTTAC

RCC1213 GAAGGGCACCACCAGGAGTGGAGCCTGCGGCTTAATTTGACTCAACACGGGGAAACTTAC

RCC1214 GAAGGGCACCACCAGGAGTGGAGCCTGCGGCTTAATTTGACTCAACACGGGGAAACTTAC

RCC1218 GAAGGGCACCACCAGGAGTGGAGCCTGCGGCTTAATTTGACTCAACACGGGGAAACTTAC

RCC1219 GAAGGGCACCACCAGGAGTGGAGCCTGCGGCTTAATTTGACTCAACACGGGGAAACTTAC

RCC1221 GAAGGGCACCACCAGGAGTGGAGCCTGCGGCTTAATTTGACTCAACACGGGGAAACTTAC

RCC1225 GAAGGGCACCACCAGGAGTGGAGCCTGCGGCTTAATTTGACTCAACACGGGGAAACTTAC

RCC1227 GAAGGGCACCACCAGGAGTGGAGCCTGCGGCTTAATTTGACTCAACACGGGGAAACTTAC

RCC1228 GAAGGGCACCACCAGGAGTGGAGCCTGCGGCTTAATTTGACTCAACACGGGGAAACTTAC

RCC1229 GAAGGGCACCACCAGGAGTGGAGCCTGCGGCTTAATTTGACTCAACACGGGGAAACTTAC

RCC1245 GAAGGGCACCACCAGGAGTGGAGCCTGCGGCTTAATTTGACTCAACACGGGGAAACTTAC

RCC1246 GAAGGGCACCACCAGGAGTGGAGCCTGCGGCTTAATTTGACTCAACACGGGGAAACTTAC

RCC1247 GAAGGGCACCACCAGGAGTGGAGCCTGCGGCTTAATTTGACTCAACACGGGGAAACTTAC

RCC1249 GAAGGGCACCACCAGGAGTGGAGCCTGCGGCTTAATTTGACTCAACACGGGGAAACTTAC

RCC1250 GAAGGGCACCACCAGGAGTGGAGCCTGCGGCTTAATTTGACTCAACACGGGGAAACTTAC

RCC1251 GAAGGGCACCACCAGGAGTGGAGCCTGCGGCTTAATTTGACTCAACACGGGGAAACTTAC

RCC1254 GAAGGGCACCACCAGGAGTGGAGCCTGCGGCTTAATTTGACTCAACACGGGGAAACTTAC

RCC1257 GAAGGGCACCACCAGGAGTGGAGCCTGCGGCTTAATTTGACTCAACACGGGGAAACTTAC

RCC1258 GAAGGGCACCACCAGGAGTGGAGCCTGCGGCTTAATTTGACTCAACACGGGGAAACTTAC

RCC1322 GAAGGGCACCACCAGGAGTGGAGCCTGCGGCTTAATTTGACTCAACACGGGGAAACTTAC

ESP7414 GAAGGGCACCACCAGGAGTGGAGCCTGCGGCTTAATTTGACTCAACACGGGGAAACTTAC

************************************************************

CCMP374 CAGGTCCAG-ACATTGTGAGGATTGACAGATTGAGAGCTCTTTCTTGATTCGATGGGTGG

920PML CAGGTCCAG-ACATTGTGAGGATTGACAGATTGAGAGCTCTTTCTTGATTCGATGGGTGG

Ch25_90 CAGGTCCAG-ACATTGTGAGGATTGACAGATTGAGAGCTCTTTCTTGATTCGATGGGTGG

CCAP920_9 CAGGTCCAG-ACATTGTGAGGATTGACAGATTGAGAGCTCTTTCTTGATTCGATGGGTGG

UNC1419 CAGGTCCAG-ACATTGTGAGGATTGACAGATTGAGAGCTCTTTCTTGATTCGATGGGTGG

RCC6856 CAGGTCCAG-ACATTGTGAGGATTGACAGATTGAGAGCTCTTTCTTGATTCGATGGGTGG

RCC909 CAGGTCCAG-ACATTGTGAGGATTGACAGATTGAGAGCTCTTTCTTGATTCGATGGGTGG

KMMCC_H-18 CAGGTCCAG-ACATTGTGAGGATTGACAGATTGAGAGCTCTTTCTTGATTCGATGGGTGG

RCC1208 CAGGTCCAGCACATTGTGAGGATTGACAGATTGAGAGCTCTTTCTTGATTCGATGGGTGG

RCC1210 CAGGTCCAGCACATTGTGAGGATTGACAGATTGAGAGCTCTTTCTTGATTCGATGGGTGG

RCC1213 CAGGTCCAGCACATTGTGAGGATTGACAGATTGAGAGCTCTTTCTTGATTCGATGGGTGG

RCC1214 CAGGTCCAGCACATTGTGAGGATTGACAGATTGAGAGCTCTTTCTTGATTCGATGGGTGG

RCC1218 CAGGTCCAGCACATTGTGAGGATTGACAGATTGAGAGCTCTTTCTTGATTCGATGGGTGG

RCC1219 CAGGTCCAGCACATTGTGAGGATTGACAGATTGAGAGCTCTTTCTTGATTCGATGGGTGG

RCC1221 CAGGTCCAGCACATTGTGAGGATTGACAGATTGAGAGCTCTTTCTTGATTCGATGGGTGG

RCC1225 CAGGTCCAGCACATTGTGAGGATTGACAGATTGAGAGCTCTTTCTTGATTCGATGGGTGG

RCC1227 CAGGTCCAGCACATTGTGAGGATTGACAGATTGAGAGCTCTTTCTTGATTCGATGGGTGG

RCC1228 CAGGTCCAGCACATTGTGAGGATTGACAGATTGAGAGCTCTTTCTTGATTCGATGGGTGG

RCC1229 CAGGTCCAGCACATTGTGAGGATTGACAGATTGAGAGCTCTTTCTTGATTCGATGGGTGG

RCC1245 CAGGTCCAGCACATTGTGAGGATTGACAGATTGAGAGCTCTTTCTTGATTCGATGGGTGG

RCC1246 CAGGTCCAGCACATTGTGAGGATTGACAGATTGAGAGCTCTTTCTTGATTCGATGGGTGG

RCC1247 CAGGTCCAGCACATTGTGAGGATTGACAGATTGAGAGCTCTTTCTTGATTCGATGGGTGG

RCC1249 CAGGTCCAGCACATTGTGAGGATTGACAGATTGAGAGCTCTTTCTTGATTCGATGGGTGG

RCC1250 CAGGTCCAGCACATTGTGAGGATTGACAGATTGAGAGCTCTTTCTTGATTCGATGGGTGG

RCC1251 CAGGTCCAGCACATTGTGAGGATTGACAGATTGAGAGCTCTTTCTTGATTCGATGGGTGG

RCC1254 CAGGTCCAGCACATTGTGAGGATTGACAGATTGAGAGCTCTTTCTTGATTCGATGGGTGG

RCC1257 CAGGTCCAGCACATTGTGAGGATTGACAGATTGAGAGCTCTTTCTTGATTCGATGGGTGG

RCC1258 CAGGTCCAGCACATTGTGAGGATTGACAGATTGAGAGCTCTTTCTTGATTCGATGGGTGG

RCC1322 CAGGTCCAGCACATTGTGAGGATTGACAGATTGAGAGCTCTTTCTTGATTCGATGGGTGG

ESP7414 CAGGTCCAGCACATTGTGAGGATTGACAGATTGAGAGCTCTTTCTTGATTCGATGGGTGG

********* **************************************************

CCMP374 TGGTGCATGGCCGTTCTTAGTTGGTGGAGTGATTTGTCTGGTTAATTCCGTTAACGAACG

920PML TGGTGCATGGCCGTTCTTAGTTGGTGGAGTGATTTGTCTGGTTAATTCCGTTAACGAACG

Ch25_90 TGGTGCATGGCCGTTCTTAGTTGGTGGAGTGATTTGTCTGGTTAATTCCGTTAACGAACG

CCAP920_9 TGGTGCATGGCCGTTCTTAGTTGGTGGAGTGATTTGTCTGGTTAATTCCGTTAACGAACG

UNC1419 TGGTGCATGGCCGTTCTTAGTTGGTGGAGTGATTTGTCTGGTTAATTCCGTTAACGAACG

RCC6856 TGGTGCATGGCCGTTCTTAGTTGGTGGAGTGATTTGTCTGGTTAATTCCGTTAACGAACG

RCC909 TGGTGCATGGCCGTTCTTAGTTGGTGGAGTGATTTGTCTGGTTAATTCCGTTAACGAACG

KMMCC_H-18 TGGTGCATGGCCGTTCTTAGTTGGTGGAGTGATTTGTCTGGTTAATTCCGTTAACGAACG

RCC1208 TGGTGCATGGCCGTTCTTAGTTGGTGGAGTGATTTGTCTGGTTAATTCCGTTAACGAACG

RCC1210 TGGTGCATGGCCGTTCTTAGTTGGTGGAGTGATTTGTCTGGTTAATTCCGTTAACGAACG

RCC1213 TGGTGCATGGCCGTTCTTAGTTGGTGGAGTGATTTGTCTGGTTAATTCCGTTAACGAACG

RCC1214 TGGTGCATGGCCGTTCTTAGTTGGTGGAGTGATTTGTCTGGTTAATTCCGTTAACGAACG

RCC1218 TGGTGCATGGCCGTTCTTAGTTGGTGGAGTGATTTGTCTGGTTAATTCCGTTAACGAACG

RCC1219 TGGTGCATGGCCGTTCTTAGTTGGTGGAGTGATTTGTCTGGTTAATTCCGTTAACGAACG

RCC1221 TGGTGCATGGCCGTTCTTAGTTGGTGGAGTGATTTGTCTGGTTAATTCCGTTAACGAACG

RCC1225 TGGTGCATGGCCGTTCTTAGTTGGTGGAGTGATTTGTCTGGTTAATTCCGTTAACGAACG

RCC1227 TGGTGCATGGCCGTTCTTAGTTGGTGGAGTGATTTGTCTGGTTAATTCCGTTAACGAACG

RCC1228 TGGTGCATGGCCGTTCTTAGTTGGTGGAGTGATTTGTCTGGTTAATTCCGTTAACGAACG

RCC1229 TGGTGCATGGCCGTTCTTAGTTGGTGGAGTGATTTGTCTGGTTAATTCCGTTAACGAACG

RCC1245 TGGTGCATGGCCGTTCTTAGTTGGTGGAGTGATTTGTCTGGTTAATTCCGTTAACGAACG

RCC1246 TGGTGCATGGCCGTTCTTAGTTGGTGGAGTGATTTGTCTGGTTAATTCCGTTAACGAACG

RCC1247 TGGTGCATGGCCGTTCTTAGTTGGTGGAGTGATTTGTCTGGTTAATTCCGTTAACGAACG

RCC1249 TGGTGCATGGCCGTTCTTAGTTGGTGGAGTGATTTGTCTGGTTAATTCCGTTAACGAACG

RCC1250 TGGTGCATGGCCGTTCTTAGTTGGTGGAGTGATTTGTCTGGTTAATTCCGTTAACGAACG

RCC1251 TGGTGCATGGCCGTTCTTAGTTGGTGGAGTGATTTGTCTGGTTAATTCCGTTAACGAACG

RCC1254 TGGTGCATGGCCGTTCTTAGTTGGTGGAGTGATTTGTCTGGTTAATTCCGTTAACGAACG

RCC1257 TGGTGCATGGCCGTTCTTAGTTGGTGGAGTGATTTGTCTGGTTAATTCCGTTAACGAACG

RCC1258 TGGTGCATGGCCGTTCTTAGTTGGTGGAGTGATTTGTCTGGTTAATTCCGTTAACGAACG

RCC1322 TGGTGCATGGCCGTTCTTAGTTGGTGGAGTGATTTGTCTGGTTAATTCCGTTAACGAACG

ESP7414 TGGTGCATGGCCGTTCTTAGTTGGTGGAGTGATTTGTCTGGTTAATTCCGTTAACGAACG

************************************************************

CCMP374 AGACCGCAGCCTGCTAAATAGCGACGCGAACCCTCCGTTCGCTGGAGCTTCTTAGAGGGA

920PML AGACCGCAGCCTGCTAAATAGCGACGCGAACCCTCCGTTCGCTGGAGCTTCTTAGAGGGA

Ch25_90 AGACCGCAGCCTGCTAAATAGCGACGCGAACCCTCCGTTCGCTGGAGCTTCTTAGAGGGA

CCAP920_9 AGACCGCAGCCTGCTAAATAGCGACGCGAACCCTCCGTTCGCTGGAGCTTCTTAGAGGGA

UNC1419 AGACCGCAGCCTGCTAAATAGCGACGCGAACCCTCCGTTCGCTGGAGCTTCTTAGAGGGA

RCC6856 AGACCGCAGCCTGCTAAATAGCGACGCGAACCCTCCGTTCGCTGGAGCTTCTTAGAGGGA

RCC909 AGACCGCAGCCTGCTAAATAGCGACGCGAACCCTCCGTTCGCTGGAGCTTCTTAGAGGGA

KMMCC_H-18 AGACCGCAGCCTGCTAAATAGCGACGCGAACCCTCCGTTCGCTGGAGCTTCTTAGAGGGA

RCC1208 AGACCGCAGCCTGCTAAATAGCGACGCGAACCCTCCGTTCGCTGGAGCTTCTTAGAGGGA

RCC1210 AGACCGCAGCCTGCTAAATAGCGACGCGAACCCTCCGTTCGCTGGAGCTTCTTAGAGGGA

RCC1213 AGACCGCAGCCTGCTAAATAGCGACGCGAACCCTCCGTTCGCTGGAGCTTCTTAGAGGGA

RCC1214 AGACCGCAGCCTGCTAAATAGCGACGCGAACCCTCCGTTCGCTGGAGCTTCTTAGAGGGA

RCC1218 AGACCGCAGCCTGCTAAATAGCGACGCGAACCCTCCGTTCGCTGGAGCTTCTTAGAGGGA

RCC1219 AGACCGCAGCCTGCTAAATAGCGACGCGAACCCTCCGTTCGCTGGAGCTTCTTAGAGGGA

RCC1221 AGACCGCAGCCTGCTAAATAGCGACGCGAACCCTCCGTTCGCTGGAGCTTCTTAGAGGGA

RCC1225 AGACCGCAGCCTGCTAAATAGCGACGCGAACCCTCCGTTCGCTGGAGCTTCTTAGAGGGA

RCC1227 AGACCGCAGCCTGCTAAATAGCGACGCGAACCCTCCGTTCGCTGGAGCTTCTTAGAGGGA

RCC1228 AGACCGCAGCCTGCTAAATAGCGACGCGAACCCTCCGTTCGCTGGAGCTTCTTAGAGGGA

RCC1229 AGACCGCAGCCTGCTAAATAGCGACGCGAACCCTCCGTTCGCTGGAGCTTCTTAGAGGGA

RCC1245 AGACCGCAGCCTGCTAAATAGCGACGCGAACCCTCCGTTCGCTGGAGCTTCTTAGAGGGA

RCC1246 AGACCGCAGCCTGCTAAATAGCGACGCGAACCCTCCGTTCGCTGGAGCTTCTTAGAGGGA

RCC1247 AGACCGCAGCCTGCTAAATAGCGACGCGAACCCTCCGTTCGCTGGAGCTTCTTAGAGGGA

RCC1249 AGACCGCAGCCTGCTAAATAGCGACGCGAACCCTCCGTTCGCTGGAGCTTCTTAGAGGGA

RCC1250 AGACCGCAGCCTGCTAAATAGCGACGCGAACCCTCCGTTCGCTGGAGCTTCTTAGAGGGA

RCC1251 AGACCGCAGCCTGCTAAATAGCGACGCGAACCCTCCGTTCGCTGGAGCTTCTTAGAGGGA

RCC1254 AGACCGCAGCCTGCTAAATAGCGACGCGAACCCTCCGTTCGCTGGAGCTTCTTAGAGGGA

RCC1257 AGACCGCAGCCTGCTAAATAGCGACGCGAACCCTCCGTTCGCTGGAGCTTCTTAGAGGGA

RCC1258 AGACCGCAGCCTGCTAAATAGCGACGCGAACCCTCCGTTCGCTGGAGCTTCTTAGAGGGA

RCC1322 AGACCGCAGCCTGCTAAATAGCGACGCGAACCCTCCGTTCGCTGGAGCTTCTTAGAGGGA

ESP7414 AGACCGCAGCCTGCTAAATAGCGACGCGAACCCTCCGTTCGCTGGAGCTTCTTAGAGGGA

************************************************************

CCMP374 CAACTTGTCTTCAACAAGTGGAAGTTCGCGGCAATAACAGGTCTGTGATGCCCTTAGATG

920PML CAACTTGTCTTCAACAAGTGGAAGTTCGCGGCAATAACAGGTCTGTGATGCCCTTAGATG

Ch25_90 CAACTTGTCTTCAACAAGTGGAAGTTCGCGGCAATAACAGGTCTGTGATGCCCTTAGATG

CCAP920_9 CAACTTGTCTTCAACAAGTGGAAGTTCGCGGCAATAACAGGTCTGTGATGCCCTTAGATG

UNC1419 CAACTTGTCTTCAACAAGTGGAAGTTCGCGGCAATAACAGGTCTGTGATGCCCTTAGATG

RCC6856 CAACTTGTCTTCAACAAGTGGAAGTTCGCGGCAATAACAGGTCTGTGATGCCCTTAGATG

RCC909 CAACTTGTCTTCAACAAGTGGAAGTTCGCGGCAATAACAGGTCTGTGATGCCCTTAGATG

KMMCC_H-18 CAACTTGTCTTCAACAAGTGGAAGTTCGCGGCAATAACAGGTCTGTGATGCCCTTAGATG

RCC1208 CAACTTGTCTTCAACAAGTGGAAGTTCGCGGCAATAACAGGTCTGTGATGCCCTTAGATG

RCC1210 CAACTTGTCTTCAACAAGTGGAAGTTCGCGGCAATAACAGGTCTGTGATGCCCTTAGATG

RCC1213 CAACTTGTCTTCAACAAGTGGAAGTTCGCGGCAATAACAGGTCTGTGATGCCCTTAGATG

RCC1214 CAACTTGTCTTCAACAAGTGGAAGTTCGCGGCAATAACAGGTCTGTGATGCCCTTAGATG

RCC1218 CAACTTGTCTTCAACAAGTGGAAGTTCGCGGCAATAACAGGTCTGTGATGCCCTTAGATG

RCC1219 CAACTTGTCTTCAACAAGTGGAAGTTCGCGGCAATAACAGGTCTGTGATGCCCTTAGATG

RCC1221 CAACTTGTCTTCAACAAGTGGAAGTTCGCGGCAATAACAGGTCTGTGATGCCCTTAGATG

RCC1225 CAACTTGTCTTCAACAAGTGGAAGTTCGCGGCAATAACAGGTCTGTGATGCCCTTAGATG

RCC1227 CAACTTGTCTTCAACAAGTGGAAGTTCGCGGCAATAACAGGTCTGTGATGCCCTTAGATG

RCC1228 CAACTTGTCTTCAACAAGTGGAAGTTCGCGGCAATAACAGGTCTGTGATGCCCTTAGATG

RCC1229 CAACTTGTCTTCAACAAGTGGAAGTTCGCGGCAATAACAGGTCTGTGATGCCCTTAGATG

RCC1245 CAACTTGTCTTCAACAAGTGGAAGTTCGCGGCAATAACAGGTCTGTGATGCCCTTAGATG

RCC1246 CAACTTGTCTTCAACAAGTGGAAGTTCGCGGCAATAACAGGTCTGTGATGCCCTTAGATG

RCC1247 CAACTTGTCTTCAACAAGTGGAAGTTCGCGGCAATAACAGGTCTGTGATGCCCTTAGATG

RCC1249 CAACTTGTCTTCAACAAGTGGAAGTTCGCGGCAATAACAGGTCTGTGATGCCCTTAGATG

RCC1250 CAACTTGTCTTCAACAAGTGGAAGTTCGCGGCAATAACAGGTCTGTGATGCCCTTAGATG

RCC1251 CAACTTGTCTTCAACAAGTGGAAGTTCGCGGCAATAACAGGTCTGTGATGCCCTTAGATG

RCC1254 CAACTTGTCTTCAACAAGTGGAAGTTCGCGGCAATAACAGGTCTGTGATGCCCTTAGATG

RCC1257 CAACTTGTCTTCAACAAGTGGAAGTTCGCGGCAATAACAGGTCTGTGATGCCCTTAGATG

RCC1258 CAACTTGTCTTCAACAAGTGGAAGTTCGCGGCAATAACAGGTCTGTGATGCCCTTAGATG

RCC1322 CAACTTGTCTTCAACAAGTGGAAGTTCGCGGCAATAACAGGTCTGTGATGCCCTTAGATG

ESP7414 CAACTTGTCTTCAACAAGTGGAAGTTCGCGGCAATAACAGGTCTGTGATGCCCTTAGATG

************************************************************

CCMP374 TTCTGGGCCGCACGCGCGCTACACTGATGCACTCAACGAGTCTATCACCTTGACCGAGAG

920PML TTCTGGGCCGCACGCGCGCTACACTGATGCACTCAACGAGTCTATCACCTTGACCGAGAG

Ch25_90 TTCTGGGCCGCACGCGCGCTACACTGATGCACTCAACGAGTCTATCACCTTGACCGAGAG

CCAP920_9 TTCTGGGCCGCACGCGCGCTACACTGATGCACTCAACGAGTCTATCACCTTGACCGAGAG

UNC1419 TTCTGGGCCGCACGCGCGCTACACTGATGCACTCAACGAGTCTATCACCTTGACCGAGAG

RCC6856 TTCTGGGCCGCACGCGCGCTACACTGATGCACTCAACGAGTCTATCACCTTGACCGAGAG

RCC909 TTCTGGGCCGCACGCGCGCTACACTGATGCACTCAACGAGTCTATCACCTTGACCGAGAG

KMMCC_H-18 TTCTGGGCCGCACGCGCGCTACACTGATGCACTCAACGAGTCTATCACCTTGACCGAGAG

RCC1208 TTCTGGGCCGCACGCGCGCTACACTGATGCACTCAACGAGTCTATCACCTTGACCGAGAG

RCC1210 TTCTGGGCCGCACGCGCGCTACACTGATGCACTCAACGAGTCTATCACCTTGACCGAGAG

RCC1213 TTCTGGGCCGCACGCGCGCTACACTGATGCACTCAACGAGTCTATCACCTTGACCGAGAG

RCC1214 TTCTGGGCCGCACGCGCGCTACACTGATGCACTCAACGAGTCTATCACCTTGACCGAGAG

RCC1218 TTCTGGGCCGCACGCGCGCTACACTGATGCACTCAACGAGTCTATCACCTTGACCGAGAG

RCC1219 TTCTGGGCCGCACGCGCGCTACACTGATGCACTCAACGAGTCTATCACCTTGACCGAGAG

RCC1221 TTCTGGGCCGCACGCGCGCTACACTGATGCACTCAACGAGTCTATCACCTTGACCGAGAG

RCC1225 TTCTGGGCCGCACGCGCGCTACACTGATGCACTCAACGAGTCTATCACCTTGACCGAGAG

RCC1227 TTCTGGGCCGCACGCGCGCTACACTGATGCACTCAACGAGTCTATCACCTTGACCGAGAG

RCC1228 TTCTGGGCCGCACGCGCGCTACACTGATGCACTCAACGAGTCTATCACCTTGACCGAGAG

RCC1229 TTCTGGGCCGCACGCGCGCTACACTGATGCACTCAACGAGTCTATCACCTTGACCGAGAG

RCC1245 TTCTGGGCCGCACGCGCGCTACACTGATGCACTCAACGAGTCTATCACCTTGACCGAGAG

RCC1246 TTCTGGGCCGCACGCGCGCTACACTGATGCACTCAACGAGTCTATCACCTTGACCGAGAG

RCC1247 TTCTGGGCCGCACGCGCGCTACACTGATGCACTCAACGAGTCTATCACCTTGACCGAGAG

RCC1249 TTCTGGGCCGCACGCGCGCTACACTGATGCACTCAACGAGTCTATCACCTTGACCGAGAG

RCC1250 TTCTGGGCCGCACGCGCGCTACACTGATGCACTCAACGAGTCTATCACCTTGACCGAGAG

RCC1251 TTCTGGGCCGCACGCGCGCTACACTGATGCACTCAACGAGTCTATCACCTTGACCGAGAG

RCC1254 TTCTGGGCCGCACGCGCGCTACACTGATGCACTCAACGAGTCTATCACCTTGACCGAGAG

RCC1257 TTCTGGGCCGCACGCGCGCTACACTGATGCACTCAACGAGTCTATCACCTTGACCGAGAG

RCC1258 TTCTGGGCCGCACGCGCGCTACACTGATGCACTCAACGAGTCTATCACCTTGACCGAGAG

RCC1322 TTCTGGGCCGCACGCGCGCTACACTGATGCACTCAACGAGTCTATCACCTTGACCGAGAG

ESP7414 TTCTGGGCCGCACGCGCGCTACACTGATGCACTCAACGAGTCTATCACCTTGACCGAGAG

************************************************************

CCMP374 GTCCGGGTAATCTTTTGAAATTGCATCGTGATGGGGATAGATTATTGCAACTATTAATCT

920PML GTCCGGGTAATCTTTTGAAATTGCATCGTGATGGGGATAGATTATTGCAACTATTAATCT

Ch25_90 GTCCGGGTAATCTTTTGAAATTGCATCGTGATGGGGATAGATTATTGCAACTATTAATCT

CCAP920_9 GTCCGGGTAATCTTTTGAAATTGCATCGTGATGGGGATAGATTATTGCAACTATTAATCT

UNC1419 GTCCGGGTAATCTTTTGAAATTGCATCGTGATGGGGATAGATTATTGCAACTATTAATCT

RCC6856 GTCCGGGTAATCTTTTGAAATTGCATCGTGATGGGGATAGATTATTGCAACTATTAATCT

RCC909 GTCCGGGTAATCTTTTGAAATTGCATCGTGATGGGGATAGATTATTGCAACTATTAATCT

KMMCC_H-18 GTCCGGGTAATCTTTTGAAATTGCATCGTGATGGGGATAGATTATTGCAACTATTAATCT

RCC1208 GTCCGGGTAATCTTTTGAAATTGCATCGTGATGGGGATAGATTATTGCAACTATTAATCT

RCC1210 GTCCGGGTAATCTTTTGAAATTGCATCGTGATGGGGATAGATTATTGCAACTATTAATCT

RCC1213 GTCCGGGTAATCTTTTGAAATTGCATCGTGATGGGGATAGATTATTGCAACTATTAATCT

RCC1214 GTCCGGGTAATCTTTTGAAATTGCATCGTGATGGGGATAGATTATTGCAACTATTAATCT

RCC1218 GTCCGGGTAATCTTTTGAAATTGCATCGTGATGGGGATAGATTATTGCAACTATTAATCT

RCC1219 GTCCGGGTAATCTTTTGAAATTGCATCGTGATGGGGATAGATTATTGCAACTATTAATCT

RCC1221 GTCCGGGTAATCTTTTGAAATTGCATCGTGATGGGGATAGATTATTGCAACTATTAATCT

RCC1225 GTCCGGGTAATCTTTTGAAATTGCATCGTGATGGGGATAGATTATTGCAACTATTAATCT

RCC1227 GTCCGGGTAATCTTTTGAAATTGCATCGTGATGGGGATAGATTATTGCAACTATTAATCT

RCC1228 GTCCGGGTAATCTTTTGAAATTGCATCGTGATGGGGATAGATTATTGCAACTATTAATCT

RCC1229 GTCCGGGTAATCTTTTGAAATTGCATCGTGATGGGGATAGATTATTGCAACTATTAATCT

RCC1245 GTCCGGGTAATCTTTTGAAATTGCATCGTGATGGGGATAGATTATTGCAACTATTAATCT

RCC1246 GTCCGGGTAATCTTTTGAAATTGCATCGTGATGGGGATAGATTATTGCAACTATTAATCT

RCC1247 GTCCGGGTAATCTTTTGAAATTGCATCGTGATGGGGATAGATTATTGCAACTATTAATCT

RCC1249 GTCCGGGTAATCTTTTGAAATTGCATCGTGATGGGGATAGATTATTGCAACTATTAATCT

RCC1250 GTCCGGGTAATCTTTTGAAATTGCATCGTGATGGGGATAGATTATTGCAACTATTAATCT

RCC1251 GTCCGGGTAATCTTTTGAAATTGCATCGTGATGGGGATAGATTATTGCAACTATTAATCT

RCC1254 GTCCGGGTAATCTTTTGAAATTGCATCGTGATGGGGATAGATTATTGCAACTATTAATCT

RCC1257 GTCCGGGTAATCTTTTGAAATTGCATCGTGATGGGGATAGATTATTGCAACTATTAATCT

RCC1258 GTCCGGGTAATCTTTTGAAATTGCATCGTGATGGGGATAGATTATTGCAACTATTAATCT

RCC1322 GTCCGGGTAATCTTTTGAAATTGCATCGTGATGGGGATAGATTATTGCAACTATTAATCT

ESP7414 GTCCGGGTAATCTTTTGAAATTGCATCGTGATGGGGATAGATTATTGCAACTATTAATCT

************************************************************

CCMP374 TCAACGAGGAATTCCTAGTAAGCGTGTGTCATCAGCGCACGTTGATTACGTCCCTGCCCT

920PML TCAACGAGGAATTCCTAGTAAGCGTGTGTCATCAGCGCACGTTGATTACGTCCCTGCCCT

Ch25_90 TCAACGAGGAATTCCTAGTAAGCGTGTGTCATCAGCGCACGTTGATTACGTCCCTGCCCT

CCAP920_9 TCAACGAGGAATTCCTAGTAAGCGTGTGTCATCAGCGCACGTTGATTACGTCCCTGCCCT

UNC1419 TCAACGAGGAATTCCTAGTAAGCGTGTGTCATCAGCGCACGTTGATTACGTCCCTGCCCT

RCC6856 TCAACGAGGAATTCCTAGTAAGCGTGTGTCATCAGCGCACGTTGATTACGTCCCTGCCCT

RCC909 TCAACGAGGAATTCCTAGTAAGCGTGTGTCATCAGCGCACGTTGATTACGTCCCTGCCCT

KMMCC_H-18 TCAACGAGGAATTCCTAGTAAGCGTGTGTCATCAGCGCACGTTGATTACGTCCCTGCCCT

RCC1208 TCAACGAGGAATTCCTAGTAAGCGTGTGTCATCAGCGCACGTTGATTACGTCCCTGCCCT

RCC1210 TCAACGAGGAATTCCTAGTAAGCGTGTGTCATCAGCGCACGTTGATTACGTCCCTGCCCT

RCC1213 TCAACGAGGAATTCCTAGTAAGCGTGTGTCATCAGCGCACGTTGATTACGTCCCTGCCCT

RCC1214 TCAACGAGGAATTCCTAGTAAGCGTGTGTCATCAGCGCACGTTGATTACGTCCCTGCCCT

RCC1218 TCAACGAGGAATTCCTAGTAAGCGTGTGTCATCAGCGCACGTTGATTACGTCCCTGCCCT

RCC1219 TCAACGAGGAATTCCTAGTAAGCGTGTGTCATCAGCGCACGTTGATTACGTCCCTGCCCT

RCC1221 TCAACGAGGAATTCCTAGTAAGCGTGTGTCATCAGCGCACGTTGATTACGTCCCTGCCCT

RCC1225 TCAACGAGGAATTCCTAGTAAGCGTGTGTCATCAGCGCACGTTGATTACGTCCCTGCCCT

RCC1227 TCAACGAGGAATTCCTAGTAAGCGTGTGTCATCAGCGCACGTTGATTACGTCCCTGCCCT

RCC1228 TCAACGAGGAATTCCTAGTAAGCGTGTGTCATCAGCGCACGTTGATTACGTCCCTGCCCT

RCC1229 TCAACGAGGAATTCCTAGTAAGCGTGTGTCATCAGCGCACGTTGATTACGTCCCTGCCCT

RCC1245 TCAACGAGGAATTCCTAGTAAGCGTGTGTCATCAGCGCACGTTGATTACGTCCCTGCCCT

RCC1246 TCAACGAGGAATTCCTAGTAAGCGTGTGTCATCAGCGCACGTTGATTACGTCCCTGCCCT

RCC1247 TCAACGAGGAATTCCTAGTAAGCGTGTGTCATCAGCGCACGTTGATTACGTCCCTGCCCT

RCC1249 TCAACGAGGAATTCCTAGTAAGCGTGTGTCATCAGCGCACGTTGATTACGTCCCTGCCCT

RCC1250 TCAACGAGGAATTCCTAGTAAGCGTGTGTCATCAGCGCACGTTGATTACGTCCCTGCCCT

RCC1251 TCAACGAGGAATTCCTAGTAAGCGTGTGTCATCAGCGCACGTTGATTACGTCCCTGCCCT

RCC1254 TCAACGAGGAATTCCTAGTAAGCGTGTGTCATCAGCGCACGTTGATTACGTCCCTGCCCT

RCC1257 TCAACGAGGAATTCCTAGTAAGCGTGTGTCATCAGCGCACGTTGATTACGTCCCTGCCCT

RCC1258 TCAACGAGGAATTCCTAGTAAGCGTGTGTCATCAGCGCACGTTGATTACGTCCCTGCCCT

RCC1322 TCAACGAGGAATTCCTAGTAAGCGTGTGTCATCAGCGCACGTTGATTACGTCCCTGCCCT

ESP7414 TCAACGAGGAATTCCTAGTAAGCGTGTGTCATCAGCGCACGTTGATTACGTCCCTGCCCT

************************************************************

CCMP374 TTGTACACACCGCCCGTCGCTCCTACCGATTGAATGATCCGGTGAGGCCCCCGGACTGCG

920PML TTGTACACACCGCCCGTCGCTCCTACCGATTGAATGATCCGGTGAGGCCCCCGGACTGCG

Ch25_90 TTGTACACACCGCCCGTCGCTCCTACCGATTGAATGATCCGGTGAGGCCCCCGGACTGCG

CCAP920_9 TTGTACACACCGCCCGTCGCTCCTACCGATTGAATGATCCGGTGAGGCCCCCGGACTGCG

UNC1419 TTGTACACACCGCCCGTCGCTCCTACCGATTGAATGATCCGGTGAGGCCCCCGGACTGCG

RCC6856 TTGTACACACCGCCCGTCGCTCCTACCGATTGAATGATCCGGTGAGGCCCCCGGACTGCG

RCC909 TTGTACACACCGCCCGTCGCTCCTACCGATTGAATGATCCGGTGAGGCCCCCGGACTGCG

KMMCC_H-18 TTGTACACACCGCCCGTCGCTCCTACCGATTGAATGATCCGGTGAGGCCCCCGGACTGCG

RCC1208 TTGTACACACCGCCCGTCGCTCCTACCGATTGAATGATCCGGTGAGGCCCCCGGACTGCG

RCC1210 TTGTACACACCGCCCGTCGCTCCTACCGATTGAATGATCCGGTGAGGCCCCCGGACTGCG

RCC1213 TTGTACACACCGCCCGTCGCTCCTACCGATTGAATGATCCGGTGAGGCCCCCGGACTGCG

RCC1214 TTGTACACACCGCCCGTCGCTCCTACCGATTGAATGATCCGGTGAGGCCCCCGGACTGCG

RCC1218 TTGTACACACCGCCCGTCGCTCCTACCGATTGAATGATCCGGTGAGGCCCCCGGACTGCG

RCC1219 TTGTACACACCGCCCGTCGCTCCTACCGATTGAATGATCCGGTGAGGCCCCCGGACTGCG

RCC1221 TTGTACACACCGCCCGTCGCTCCTACCGATTGAATGATCCGGTGAGGCCCCCGGACTGCG

RCC1225 TTGTACACACCGCCCGTCGCTCCTACCGATTGAATGATCCGGTGAGGCCCCCGGACTGCG

RCC1227 TTGTACACACCGCCCGTCGCTCCTACCGATTGAATGATCCGGTGAGGCCCCCGGACTGCG

RCC1228 TTGTACACACCGCCCGTCGCTCCTACCGATTGAATGATCCGGTGAGGCCCCCGGACTGCG

RCC1229 TTGTACACACCGCCCGTCGCTCCTACCGATTGAATGATCCGGTGAGGCCCCCGGACTGCG

RCC1245 TTGTACACACCGCCCGTCGCTCCTACCGATTGAATGATCCGGTGAGGCCCCCGGACTGCG

RCC1246 TTGTACACACCGCCCGTCGCTCCTACCGATTGAATGATCCGGTGAGGCCCCCGGACTGCG

RCC1247 TTGTACACACCGCCCGTCGCTCCTACCGATTGAATGATCCGGTGAGGCCCCCGGACTGCG

RCC1249 TTGTACACACCGCCCGTCGCTCCTACCGATTGAATGATCCGGTGAGGCCCCCGGACTGCG

RCC1250 TTGTACACACCGCCCGTCGCTCCTACCGATTGAATGATCCGGTGAGGCCCCCGGACTGCG

RCC1251 TTGTACACACCGCCCGTCGCTCCTACCGATTGAATGATCCGGTGAGGCCCCCGGACTGCG

RCC1254 TTGTACACACCGCCCGTCGCTCCTACCGATTGAATGATCCGGTGAGGCCCCCGGACTGCG

RCC1257 TTGTACACACCGCCCGTCGCTCCTACCGATTGAATGATCCGGTGAGGCCCCCGGACTGCG

RCC1258 TTGTACACACCGCCCGTCGCTCCTACCGATTGAATGATCCGGTGAGGCCCCCGGACTGCG

RCC1322 TTGTACACACCGCCCGTCGCTCCTACCGATTGAATGATCCGGTGAGGCCCCCGGACTGCG

ESP7414 TTGTACACACCGCCCGTCGCTCCTACCGATTGAATGATCCGGTGAGGCCCCCGGACTGCG

************************************************************

CCMP374 GCGCCGCAGCTGGTTCTCCAGCCGCGACGCCGCGGGAAGCTGTCCGAACCTTATCA----

920PML GCGCCGCAGCTGGTTCTCCAGCCGCGACGCCGCGGGAAGCTGTCCGAACCTTATCA----

Ch25_90 GCGCCGCAGCTGGTTCTCCAGCCGCGACGCCGCGGGAAGCTGTCCGAACCTTATCA----

CCAP920_9 GC----------------------------------------------------------

UNC1419 GCGCCGCAGCTGGTTCTCCAGCCGCGACGCCGCGGGAAGCTGTCCGAACCTTATCATTTA

RCC6856 GCGCCGCAGCTGGTTCTCCAGCCGCGACGCCGC---------------------------

RCC909 GCGCCGCAGCTGGTTCTCCAGCCGCGACGCCGCGGGAAGC--------------------

KMMCC_H-18 GCGCCGCAGCTGGTTCTCCAGTCGCGACGCCGCGGGAAGCTGTCCGAACCTTATCATTTA

RCC1208 GCGCCGCAGCTGGTTCTCCAGCCGCGACGCCGCGGGAAGCTGTCCGAACCTTATCATTTA

RCC1210 GCGCCGCAGCTGGTTCTCCAGCCGCGACGCCGCGGGAAGCTGTCCGAACCTTATCATTTA

RCC1213 GCGCCGCAGCTGGTTCTCCAGCCGCGACGCCGCGGGAAGCTGTCCGAACCTTATCATTTA

RCC1214 GCGCCGCAGCTGGTTCTCCAGCCGCGACGCCGCGGGAAGCTGTCCGAACCTTATCATTTA

RCC1218 GCGCCGCAGCTGGTTCTCCAGCCGCGACGCCGCGGGAAGCTGTCCGAACCTTATCATTTA

RCC1219 GCGCCGCAGCTGGTTCTCCAGCCGCGACGCCGCGGGAAGCTGTCCGAACCTTATCATTTA

RCC1221 GCGCCGCAGCTGGTTCTCCAGCCGCGACGCCGCGGGAAGCTGTCCGAACCTTATCATTTA

RCC1225 GCGCCGCAGCTGGTTCTCCAGCCGCGACGCCGCGGGAAGCTGTCCGAACCTTATCATTTA

RCC1227 GCGCCGCAGCTGGTTCTCCAGCCGCGACGCCGCGGGAAGCTGTCCGAACCTTATCATTTA

RCC1228 GCGCCGCAGCTGGTTCTCCAGCCGCGACGCCGCGGGAAGCTGTCCGAACCTTATCATTTA

RCC1229 GCGCCGCAGCTGGTTCTCCAGCCGCGACGCCGCGGGAAGCTGTCCGAACCTTATCATTTA

RCC1245 GCGCCGCAGCTGGTTCTCCAGCCGCGACGCCGCGGGAAGCTGTCCGAACCTTATCATTTA

RCC1246 GCGCCGCAGCTGGTTCTCCAGCCGCGACGCCGCGGGAAGCTGTCCGAACCTTATCATTTA

RCC1247 GCGCCGCAGCTGGTTCTCCAGCCGCGACGCCGCGGGAAGCTGTCCGAACCTTATCATTTA

RCC1249 GCGCCGCAGCTGGTTCTCCAGCCGCGACGCCGCGGGAAGCTGTCCGAACCTTATCATTTA

RCC1250 GCGCCGCAGCTGGTTCTCCAGCCGCGACGCCGCGGGAAGCTGTCCGAACCTTATCATTTA

RCC1251 GCGCCGCAGCTGGTTCTCCAGCCGCGACGCCGCGGGAAGCTGTCCGAACCTTATCATTTA

RCC1254 GCGCCGCAGCTGGTTCTCCAGCCGCGACGCCGCGGGAAGCTGTCCGAACCTTATCATTTA

RCC1257 GCGCCGCAGCTGGTTCTCCAGCCGCGACGCCGCGGGAAGCTGTCCGAACCTTATCATTTA

RCC1258 GCGCCGCAGCTGGTTCTCCAGCCGCGACGCCGCGGGAAGCTGTCCGAACCTTATCATTTA

RCC1322 GCGCCGCAGCTGGTTCTCCAGCCGCGACGCCGCGGGAAGCTGTCCGAACCTTATCATTTA

ESP7414 GCGCCGCAGCTGGTTCTCCAGCCGCGACGCCGCGGGAAGCTGTCCGAACCTTATCATTTA

**

CCMP374 -----------------------------

920PML -----------------------------

Ch25_90 -----------------------------

CCAP920_9 -----------------------------

UNC1419 GAGGAAGGAGAAGTCGTAACAAGGTTTCC

RCC6856 -----------------------------

RCC909 -----------------------------

KMMCC_H-18 GAGGAAGGAGAAATCGTAACAAGG-----

RCC1208 GAGGAAGGAGAAGTCGTAACAAGGTTTCC

RCC1210 GAGGAAGGAGAAGTCGTAACAAGGTTTCC

RCC1213 GAGGAAGGAGAAGTCGTAACAAGGTTTCC

RCC1214 GAGGAAGGAGAAGTCGTAACAAGGTTTCC

RCC1218 GAGGAAGGAGAAGTCGTAACAAGGTTTCC

RCC1219 GAGGAAGGAGAAGTCGTAACAAGGTTTCC

RCC1221 GAGGAAGGAGAAGTCGTAACAAGGTTTCC

RCC1225 GAGGAAGGAGAAGTCGTAACAAGGTTTCC

RCC1227 GAGGAAGGAGAAGTCGTAACAAGGTTTCC

RCC1228 GAGGAAGGAGAAGTCGTAACAAGGTTTCC

RCC1229 GAGGAAGGAGAAGTCGTAACAAGGTTTCC

RCC1245 GAGGAAGGAGAAGTCGTAACAAGGTTTCC

RCC1246 GAGGAAGGAGAAGTCGTAACAAGGTTTCC

RCC1247 GAGGAAGGAGAAGTCGTAACAAGGTTTCC

RCC1249 GAGGAAGGAGAAGTCGTAACAAGGTTTCC

RCC1250 GAGGAAGGAGAAGTCGTAACAAGGTTTCC

RCC1251 GAGGAAGGAGAAGTCGTAACAAGGTTTCC

RCC1254 GAGGAAGGAGAAGTCGTAACAAGGTTTCC

RCC1257 GAGGAAGGAGAAGTCGTAACAAGGTTTCC

RCC1258 GAGGAAGGAGAAGTCGTAACAAGGTTTCC

RCC1322 GAGGAAGGAGAAGTCGTAACAAGGTTTCC

ESP7414 GAGGAAGGAGAAGTCGTAACAAGGTTTCC

Supplementary Fig S4. Alignment of 18S sequences in GenBank. Differences colored in red. Alignment was performed with ClustalW 2.1

Sequences used: CCMP374 L04957.2; 920PML M87327.2; Ch25_90 AF184167.1; CCAP920_9 MG022751.1; UNC1419 KX229688.1; RCC6856 MN824007.1; RCC909 KT861255.1; KMMCC_H-18 HQ877901.1; RCC1208 KC404120.1; RCC1210 KC404121.1; RCC1213 KC404122.1; RCC1214 KC404123.1; RCC1218 KC404124.1; RCC1219 KC404125.1; RCC1221 KC404126.1; RCC1225 KC404127.1; RCC1227 KC404128.1; RCC1228 KC404129.1; RCC1229 KC404130.1; RCC1245 KC404131.1; RCC1246 KC404132.1; RCC1247 KC404133.1; RCC1249 KC404134.1; RCC1250 KC404135.1; RCC1251 KC404136.1; RCC1254 KC404137.1; RCC1257 KC404138.1; RCC1258 KC404139.1; RCC1322 KC404140.1; ESP7414 KC404141.1


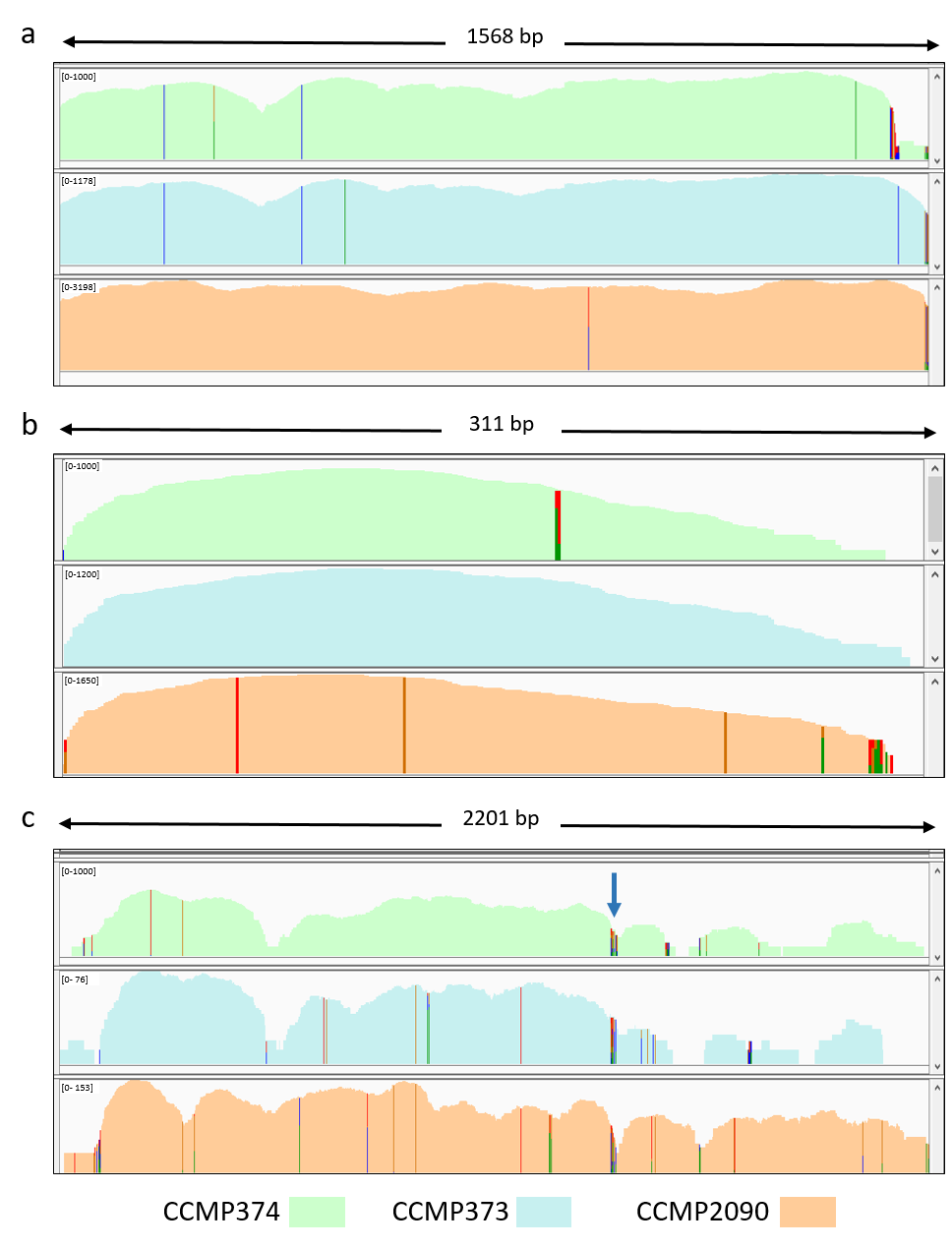


Supplementary Fig S5. Read coverage and SNPs of three genes in three strains. (a) The manually defined gene Spt1 (comp17050001_c0), and the automatically defined genes (b) comp92716_c0 and (c) comp91116_c0. The vertical lines represent mismatches to the defined transcripts. Most differences are between the strains. Note the blue arrow that indicates an area where the three strains differ from the defined transcript, probably representing a misassembly. Visualization of reads on the transcriptome was performed using the IGV browser (<http://www.broadinstitute.org/igv/>).


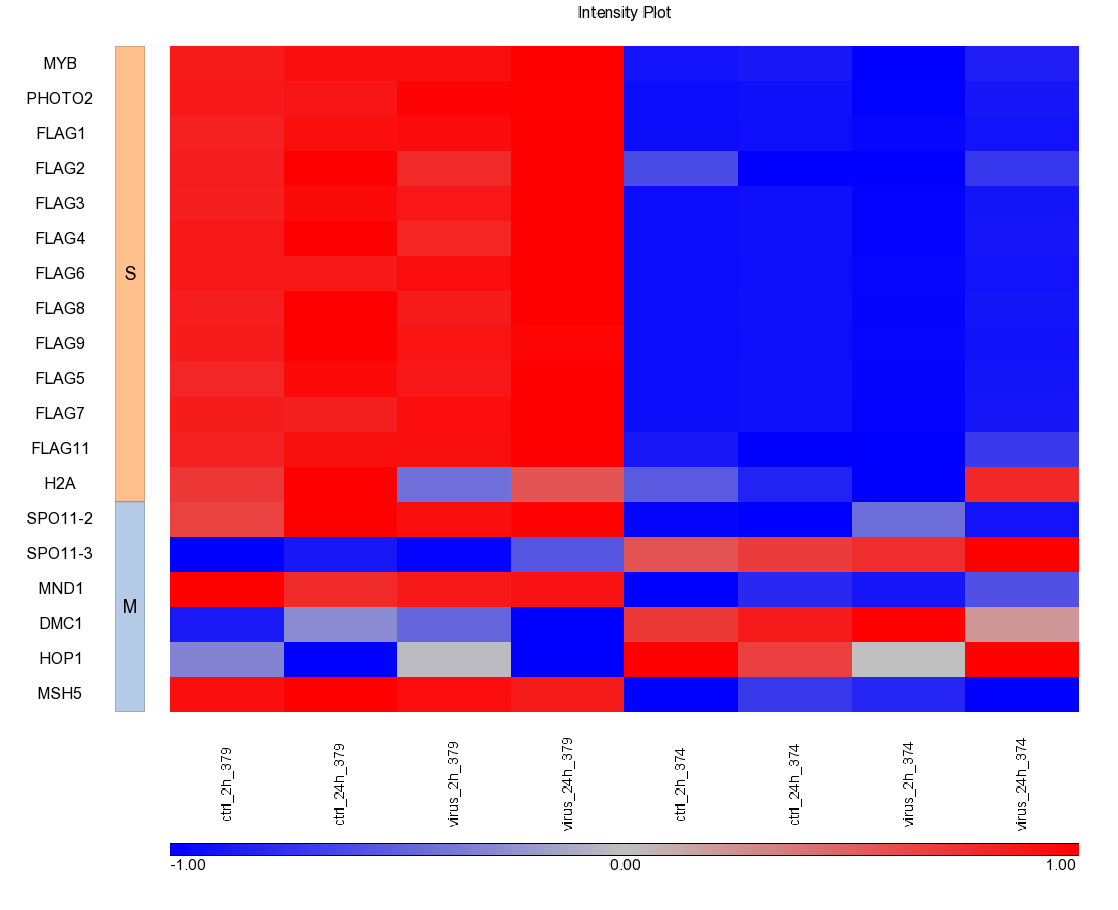


a


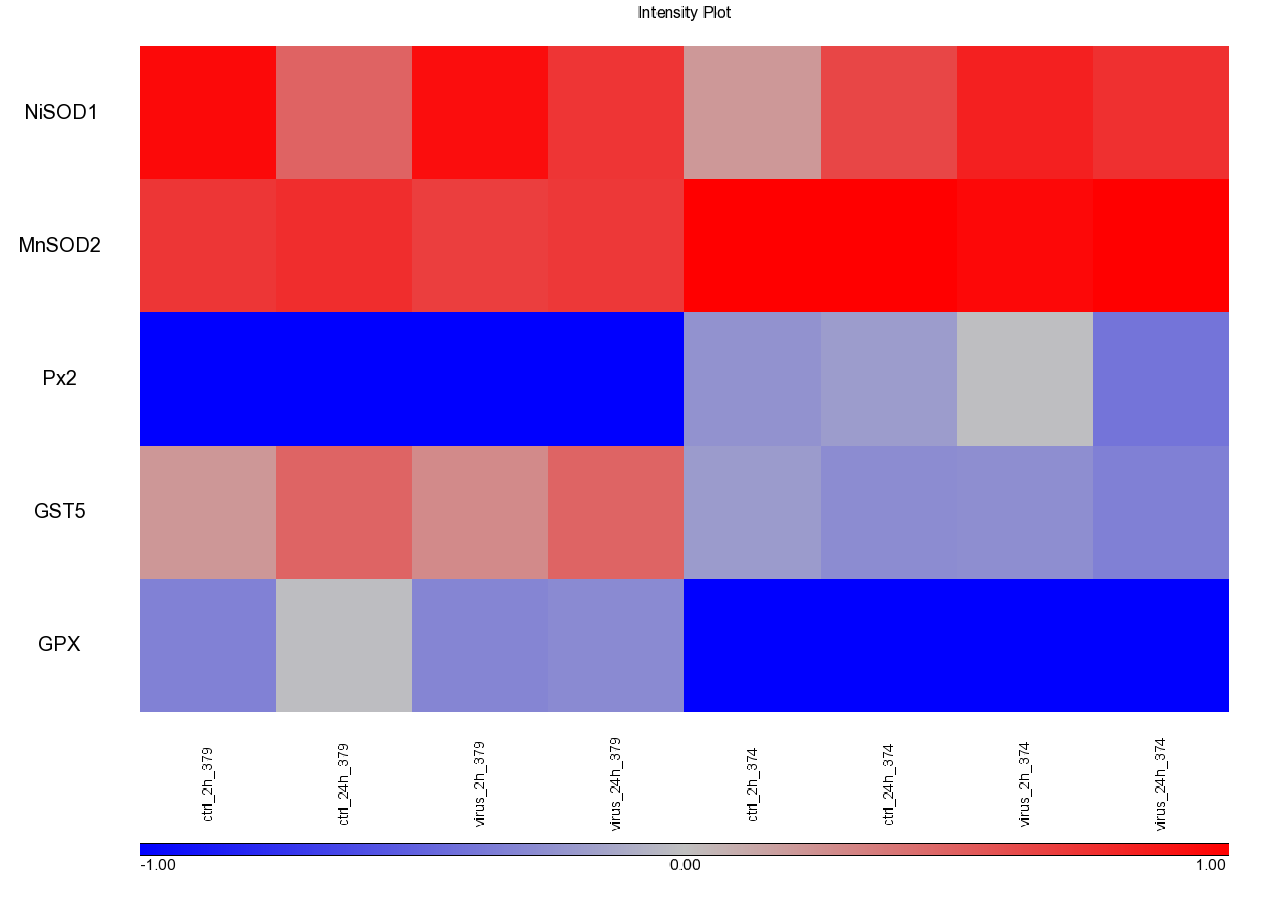


b


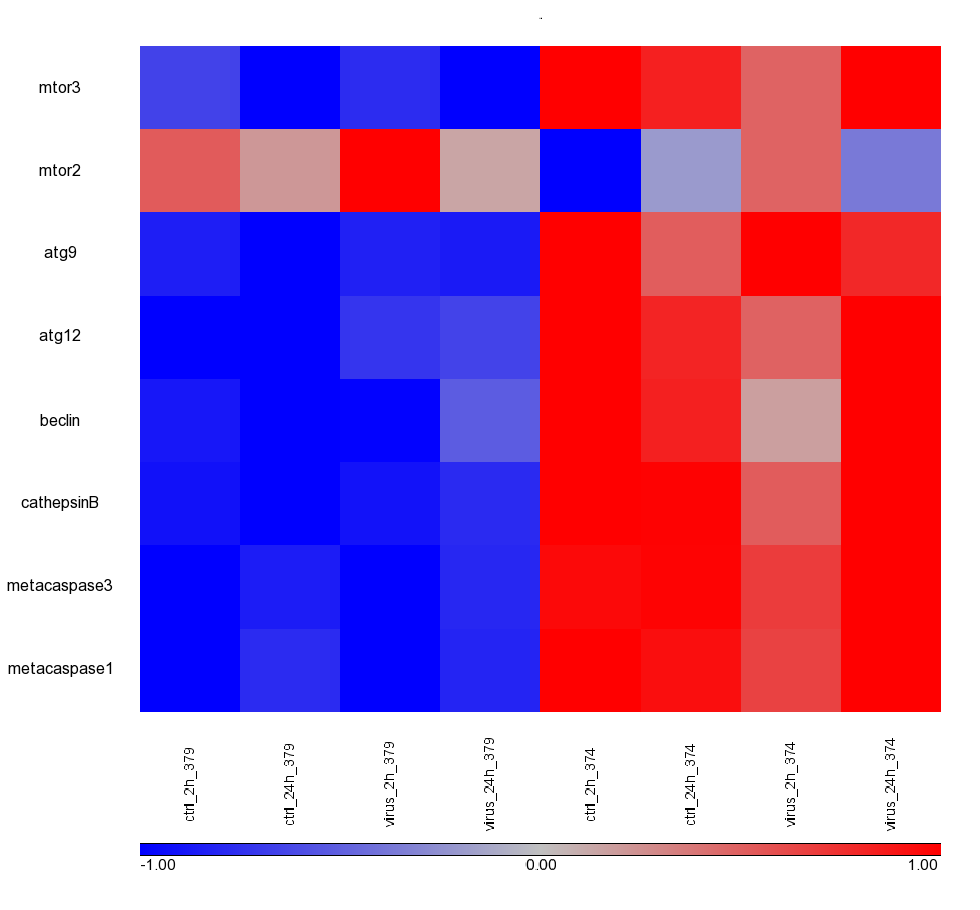


c

Supplementary Fig S6. Heatmaps of gene groups in CCMP379 (resistant) and CCMP374 (sensitive). For each strain there are four samples, 2 hr and 24 hr control, and 2 hr and 24 hr post viral (EhV86) infection. The gene groups are: a. S-cell genes (orange side bar) and meiosis genes (blue side bar) b. ROS related genes from cluster 5 c. cell death related genes from cluster 3. The color scale represents standardized rld (log_2_) expression values.
